# EpiMII: Structure-Aware Graph Neural Networks for MHC-II Epitope Generation

**DOI:** 10.34133/research.1311

**Published:** 2026-06-15

**Authors:** Jiayi Yuan, Xiaowei Xu, Ze-Yu Sun, Shan Zhu, Chunyu Wei, Tianjian Liang, Jingxuan Ge, Xiang-Qun Xie, Yan Chen, Zhiwei Feng, Tingjun Hou, Ying Xue

**Affiliations:** ^1^ Department of Pharmaceutical Sciences, Computational Chemical Genomics Screening Center, and Pharmacometrics & System Pharmacology PharmacoAnalytics, School of Pharmacy; National Center of Excellence for Computational Drug Abuse Research, University of Pittsburgh, Pittsburgh, PA 15261, USA.; ^2^ College of Pharmacology Sciences, Zhejiang University of Technology, Hangzhou 313099, Zhejiang, China.; ^3^ Faculty of Pharmaceutical Sciences, Shenzhen University of Advanced Technology, Shenzhen 518107, Guangdong, China.; ^4^ College of Pharmaceutical Sciences, Zhejiang University, Hangzhou 310058, Zhejiang, China.; ^5^ Department of Pharmacy; Center of Excellence for Intelligent Pharmacy and Precision Pharmacotherapy (CE-IPPP); Shanghai International Medical Innovation Center (SIMIC); National Medical Center, Zhongshan Hospital, Fudan University, Shanghai 200032, China.

## Abstract

Major histocompatibility complex class II (MHC-II) neoantigens play a critical role in immunotherapy, either as direct effectors or through their influence on CD8^+^ T cells. However, only a small fraction of tumor DNA mutations qualify as functional neoantigens, and current prediction tools suffer from limited accuracy, making the in vivo design of highly immunogenic neoantigens challenging. Here, we present EpiMII, a structure-aware graph neural network model for de novo design of MHC-II epitopes. By integrating 3-dimensional structural information through an inverse folding strategy, EpiMII generates mimotopes that preserve T cell specificity while enhancing MHC-II binding affinity and immunogenicity. The model was trained on a first-of-its-kind curated dataset of 142,934 homology-modeled MHC-II epitope structures, overcoming the scarcity of experimentally resolved MHC-II–peptide complexes. Benchmarking analyses illustrate that EpiMII achieves a sequence recovery rate of 66.7% on a held-out test set and 79.0% on crystallized epitopes, markedly outperforming existing tools like ProteinMPNN. In a hepatocellular carcinoma case study, all 5 EpiMII-designed epitopes could markedly activate CD4^+^ T cells in vitro and induce the secretion of interferon-γ and tumor necrosis factor-α. Notably, treatment with one epitope (P4) substantially reduced tumor volume in mice in vivo, comparable to a known positive control. These findings establish EpiMII as a powerful tool for structure-guided neoantigen discovery, with broad implications for cancer vaccine development and personalized immunotherapy.

## Introduction

Neoantigens, derived from nonsynonymous somatic mutations in tumor cells and absent in normal cells, are recognized as non-self-antigens, evoking immune responses unimpeded by central or peripheral tolerance mechanisms [1–3]. In cancer patients, neoantigen-specific T cells are frequently detected in the tumor microenvironment and peripheral circulation before and during immunotherapies, including immune checkpoint blockade and personalized cancer vaccines [4]. Early clinical trials of neoantigen-based therapeutic vaccines, administered as monotherapies or in combination with immune checkpoint inhibitors (ICIs), have demonstrated promising efficacy for standard cancer treatment [5–7].

Recent studies have highlighted the critical roles of major histocompatibility complex class II (MHC-II)-restricted CD4^+^ T cell responses to tumor neoantigens during immunotherapy [8], functioning either as direct effectors or through indirect influence on CD8^+^ T cells [4]. As the key to CD4^+^ T cell activation, MHC-II expression has been detected in multiple human tumor cells, including melanoma, ovarian cancer, and lung cancer [9]. Besides, even in the absence of intrinsic tumor cell MHC-II expression, CD4^+^ T cells have the capability to mediate antitumor effects [10], a phenomenon that contrasts with the MHC-I antigen presentation pathway, which is frequently compromised by immune escape mechanisms that reduce the efficacy of MHC-I epitopes. Accumulating evidence demonstrates that CD4^+^ T cell responses to MHC-II epitopes are indispensable not only for optimal priming of MHC-I-restricted CD8^+^ T cells and their mutation into cytotoxic T cells but also for robust responses to ICIs [8,11,12]. Regulatory T cells (Treg) are known to suppress antitumor response; however, CD4^+^ T cells can counteract Treg-mediated inhibition when vaccines contain both MHC-I neoantigens and low-dose MHC-II-restricted neoantigens, thereby promoting tumor rejection [13]. These findings collectively position MHC-II neoantigens as highly promising targets for cancer therapy. However, only a small fraction of DNA mutations in cancer cells qualify as functional neoantigen. The majority of mutations are patient-specific, with limited sharing across individuals [1], which restricts the development of MHC-II neoantigen-based cancer immunotherapy at the personal level but not at the population level.

Current computational strategies for MHC-II neoantigen discovery are primarily sequence-based approaches, which can be broadly categorized into 2 classes. The first class focuses on predicting peptide–MHC-II binding potential using sequence-derived features, as exemplified by NetMHCIIpan 4.3 [14,15] and MixMHC2pred 2 [16], which are trained on large-scale peptide-binding affinity datasets and ligandome mass-spectrometry data. The second class of models directly predicts peptide immunogenicity, estimating the likelihood of eliciting CD4^+^ T cell responses from sequence-based representations. Examples include FIONA [17], TLImmuno2 [18], DeepNeo-v2[19], and CD4EpiScore [20]. More recent efforts integrate both tasks within unified frameworks, such as ImmuScope [21], which incorporates antigen presentation, CD4+ T cell epitopes, and immunogenicity assessment; MARIA [22], which employs recurrent neural networks to jointly model binding and T cell activation; and Neo-intline [23], which combines peptide binding, TCR recognition, and downstream immunogenicity predictions. In parallel, physics-informed approaches, such as free energy perturbation-assisted machine learning (FEPaML) [24], have been developed to incorporate free-energy perturbation calculations with machine learning to identify energetically favorable peptide–MHC-II binding.

Despite notable progress, current sequence-based models remain fundamentally constrained. Although these approaches can achieve relatively high overall accuracy and recall for peptide–MHC-II binding prediction, they rely almost exclusively on static sequence-binding potential pairs and fail to incorporate explicit structural context. As a result, they struggle to distinguish truly immunogenic neoantigens from highly similar wild-type peptides, where subtle conformational determinants often govern T cell recognition. Furthermore, the scarcity of high-quality training data for many rare or underrepresented MHC-II alleles severely limits model generalizability, resulting in substantial performance drops when applied to unseen HLA types. Together, these limitations underscore the urgent need for integrative frameworks that leverage structural information to enhance neoantigen discovery.

Inverse folding algorithm offers a promising solution to overcome existing limitations by inverting the conventional “sequence-to-structure” paradigm. Instead of predicting structures from sequences, this approach begins with a fixed 3-dimensional (3D) backbone and explores sequence space to identify residues optimally compatible with the given geometry. Unlike sequence-based ranking methods, this strategy leverages atomic-level details, including hydrogen-bond patterns, side-chain packing, and electrostatics, to encode high-resolution constraints that sequence-only models cannot capture [25]. Recent graph neural network (GNN) implementations, such as ProteinMPNN, have demonstrated that conditioning on backbone structural context markedly improves sequence recovery and functional protein design across a broad range of scaffolds, highlighting the value of structural information in discriminating subtle mutation effects [26]. While critical for immuno-oncology applications, particularly in the direct engineering of mimotopes that improve antigen presentation without disrupting the T cell contact surface, no model has yet applied inverse folding algorithms to mimotope design.

To break the performance ceiling of sequence-only scoring models, we shift from sequence-ranking to structure-conditioned epitope design. Mimotopes are short peptides that reproduce the essential recognition features of a native epitope while diverging at the sequence level, were first identified in phage-display systems as structural surrogates of conformational B cell epitopes [27–29]. In the T cell setting, mimotopes can be generated by introducing targeted amino acid substitutions that preserve the TCR-contact geometry of an epitope yet adjust anchor or peripheral residues to modulate recognition [30,31]. This conceptual shift allows engineered mimotopes to retain antigen specificity while improving stability, binding, or functional potency [30,31]. Based on this framework, we introduce EpiMII (EpiMPNN-MHCII), an inverse folding GNN that designs mimotopes by introducing targeted amino acid mutations into the 3D backbone of the MHC-II epitope complex. These engineered mimotopes retain the T cell specificity of native epitope while maintaining MHC-II binding affinity and exhibiting comparable or enhanced CD4^+^ immunogenicity. We curated the first structure dataset of MHC-II epitopes, enabling the EpiMII model to learn geometric, chemical, and immunological features simultaneously rather than relying on sequences alone. EpiMII achieves 66.7% sequence recovery on a held-out test set from our structure database and 79.0% recovery for epitopes with experimentally resolved crystal structures. In a hepatocellular carcinoma (HCC) case study, the neoantigen mimotopes designed by EpiMII elicited robust interferon-γ (IFN-γ), tumor necrosis factor-α (TNF-α) and interleukin-4 (IL-4) responses in vitro and demonstrated therapeutic benefit in an HCC mouse model. These results establish EpiMII as the first structure-guided design framework for MHC-II mimotope engineering and demonstrate that combining high-quality structural data with a message-passing architecture substantially advances T cell-targeted therapeutic epitope design.

## Results

### Critical role of 3D structures of epitopes in MHC-II binding

**Fig. 1.**
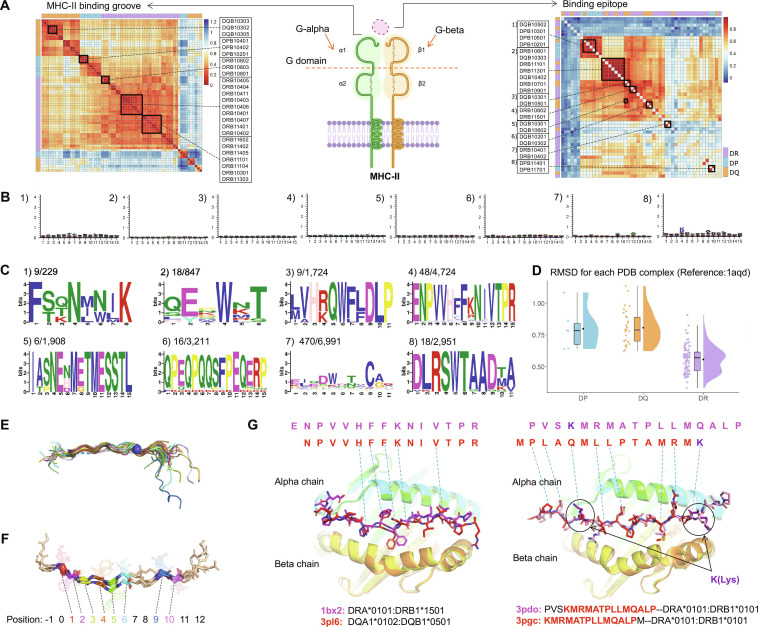
The overall sequence and structure analyses of MHC-II molecules and their epitopes. (A) The left panel shows the pairwise Euclidean distances of the G-domains. Alleles with distances <0.2 are highlighted in black squares, indicating clusters of genetically related alleles within the same HLA locus. The alleles’ names are shown on the right. The middle panel of (A) illustrates the structural architecture of MHC-II-epitope complexes, with the G-domain (composed of the α1 and β1 regions). The right panel shows the pairwise correlation coefficients for 20 amino acids’ frequencies at each position of epitopes bound to specific MHC-II alleles. Clusters with correlation >0.9 (black squares) reveal conserved positional amino acid frequency patterns, with allele names annotated on the left. (B) Sequence logo plots for the epitope sequences in the 8 groups selected in (A) the right panel. (C) Eight sequence logo plots of the most frequently shared motif for each epitope group from the right panel of (A) (created by MEME). The most frequently shared motif refers to the motif that is shared by the largest number of epitopes in this group. 9/229 means that 9 epitopes share this motif in all 229 epitopes. (D) RMSDs of 133 cocrystallized MHC-II-epitope complexes, grouped as 3 MHC-II types: DR (mean: 0.5581), DQ (mean: 0.8069), and DP (mean: 0.7985). (E) The structural alignment of 133 cocrystallized MHC-II epitopes (stick representations). (F) The structural alignment of 5 cocrystallized epitopes, including 1bx2 (DR), 1klg (DR), 2nna (DQ), 4p5k (DP), and 5ujt (DQ), with numbered positions. Eight highly overlapped epitope positions (1 to 6, 9, and 10) are highlighted as colorful sticks. (G) The left panel is the structural alignment of 1bx2 (magenta) and 3pl6 (red), and the right panel is the structural alignment of 3pdo (magenta) and 3pgc (red), with a K(Lys) residue circled, indicating the start of the identical sequences. Dashed lines indicate residue annotations. Panel (A) was created with BioRender.com.

One major challenge in identifying epitopes that participate in the MHC-II antigen presentation pathway is the extensive polymorphism of HLA class II alleles. We first collected the sequences of 66 HLA class II alleles that cover around 99% of the population worldwide, including DRB1, DPB1, DQB1, DRA, DPA1, and DQA1 HLA locus. Analysis of the pairwise Euclidean distances of the G-domains (MHC-II binding groove) across these 66 alleles demonstrate high intragroup similarities to alleles within the same HLA locus (Fig. 1A). This is consistent with the nomenclature of HLA alleles; alleles like HLA-DRB1*08 represent a group of homologous sequences encoding the same antigen [32]. Another challenge lies in the open-ended architecture of the MHC-II binding groove, which permits binding of variable-length epitopes. Pairwise correlation analysis of 15-mer epitopes bound to different MHC-II types illustrates low intragroup similarities but reveals 8 cross-group clusters with high sequence similarities (>0.9) (Fig. 1A). This suggests that the epitopes bound to an MHC-II group have similar amino acid frequency patterns. However, sequence logo analysis for each cluster shows no dominant residue at any position (Fig. 1B). We then identified the shared motifs within each epitope group using MEME [33], though the most prevalent motif in any group accounts for less than 6% of epitopes (Fig. 1C), indicating no common sequence pattern across diverse MHC-II-bound epitopes.

From a structural perspective, we conducted structural alignment of 133 x-ray crystalized pMHC-II complexes from the Protein Data Bank (PDB), using PDB:1aqd as the reference template to calculate root-mean-square deviations (RMSDs). All 3 groups (DR, DQ, and DP) exhibit low structural variability (Fig. 1D). Notably, DR–epitope complexes exhibit lower RMSDs (mean: 0.56 Å) compared to DQ/DP–epitope complexes (means: 0.80 Å) (Fig. 1D). The backbone structural alignment of all cocrystallized MHC-II epitopes indicates conserved conformational alignment in the central region, while the N- and C-termini display greater positional flexibility (Fig. 1E and F). Two representative structure alignments are shown in Fig. 1G, demonstrating that identical epitope sequences can either bind to different types of MHC-IIs or adopt reversed binding orientations on the same MHC-II (Fig. 1G), a phenomenon previously observed in DP complexes [16]. For further analysis, we defined the first highly superimposed residues as position 1, with positional numbering extending outward. The 8 most overlapped epitope positions bound to all MHC-II types are identified (Fig. 1F).

**Fig. 2.**
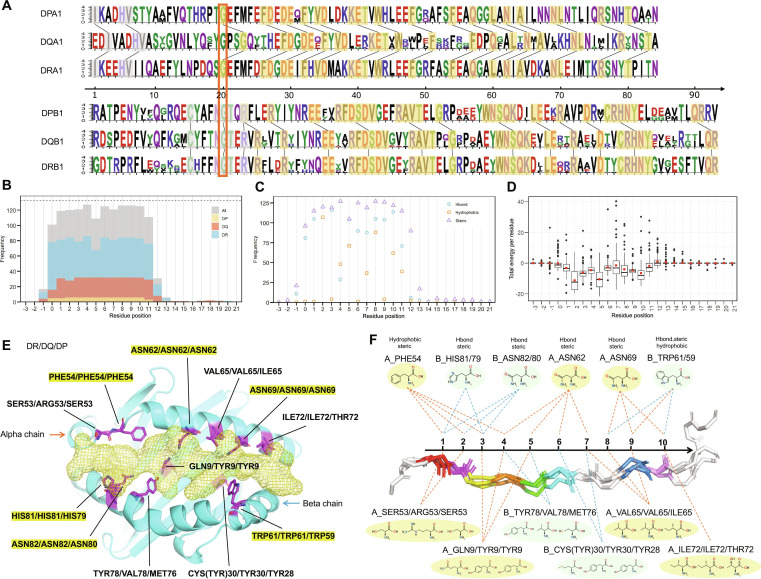
The detailed sequence and structure analyses of the key binding residues and positions on MHC-II and epitopes. (A) Sequence logo plot of 66 HLA class II alleles with the conserved patterns across different MHC-II types (highlighted in yellow and gray). The 66 alleles are divided into DPA1, DQA1, DRA1, DPB1, DQB1, and DRB1 groups. The *x*-axis shows the positions in the aligned G-domain sequences. A glycine at position 20 (marked with an orange rectangle) indicates a different alignment pattern preceding and succeeding this position. (B) and (C) show the MCCS scoring results of 133 cocrystallized epitopes using the position numbers in Fig. 1E. (B) The overlapping histograms of all 133 DR-bound, DQ-bound, and DP-bound epitopes indicate how many residues at each position have high-energy contributions (<−0.7 kcal/mol) to MHC-II binding. (C) The dots indicate how many residues substantially contribute (<−0.3 kcal/mol) to hydrogen bonds, hydrophobic interactions, or steric interactions at each position. (D) Each black dot represents the total energy for one residue at a specific position on the epitope sequences calculated by the Rosetta energy splitting algorithm. The lower, the better. (E) The overview of the aligned residues on the MHC-II binding groove of DR/DQ/DP that highly contribute to the epitope binding calculated using MCCS. Conserved residues shared by all MHC-II are highlighted in yellow. The others are nonconserved residues. (F) A schematic diagram of the MCCS results demonstrates which epitope positions interact with the conserved (above the *x*-axis) and nonconserved (below *x*-axis) residues on the MHC-II binding groove. Dashed lines show the positions of the residues that interact with the MHC-II binding groove. The residues are shown as DR/DQ/DP.

We also conducted sequence alignments of the G-domains of 66 HLA class II alleles, identifying several conserved motifs within their binding grooves (Fig. 2A). G-beta displayed a greater number of consecutive motifs compared to G-alpha. In G-alpha, alleles on the DRA1 locus have conserved motifs (e.g., “FD”) that shared with the DPA1 locus at the same position, while their corresponding positions are shifted 3 units to the right on DQA1 locus. In G-beta, the conserved motifs such as “RFDSDV” in DRB1 occupy identical positions to those in DQB1, but these motifs shift 2 units leftward in the DPB1 locus (Fig. 2A).

We then applied the molecular complex characterizing system (MCCS) scoring technique to the 133 crystalized MHC-II-epitope complexes (pMHC-IIs) to identify key residues in both the MHC-II binding groove and epitope sequence positions. Approximately 120 cocrystallized epitopes show high total energy contributions (<−0.7 kcal/mol) at positions 1 to 10 (Fig. 2B and C), consistent with the Rosetta energy decomposition analyses [34], which confirmed the core binding regions on MHC-II epitope sequences (Fig. 2D). Residues in this region can form steric interactions with MHC-II, while residues at positions 1, 3, 5, 8, 9, and 10 interact with MHC-II via hydrogen bonds with frequencies > 100 (Fig. 2C). Only position 2 has more than 100 residues forming hydrophobic interactions with MHC-II (Fig. 2C). The detailed residue interactions in the DR, DP, and DQ groups are presented in Fig. S1. For the MHC-II G-domain, the scoring results revealed 6 conserved and 6 nonconserved residues with high energy contributions to the binding across all 133 pMHC-IIs (Fig. 2E and Fig. S2). The conserved residues mainly interact with positions 1 to 10 via hydrogen bonds and steric interactions. A_PHE54 and B_TRP61/59 also form hydrophobic interactions (Fig. 2F). Six nonconserved residues at the corresponding positions influence binding at positions 1 to 10 (Fig. 2F), potentially determining MHC-II epitope binding specificity. The detailed interactions for both conserved and nonconserved residues of G-domain are illustrated in Fig. S3 and Table S1. The experimentally defined anchor region (P1 to P9 in PDB 1J8H) corresponds to positions 2 to 10 in our case (PDB: 1bx2), where conserved MHC-II residues impose hydrogen bonds to stabilize peptide binding [35,36]. In previous experimental structural analyses of pMHC-IIs, the interactions of anchor residues P1 (position 2), P4 (position 5), P6 (position 7), and P9 (position 10) received widespread attention [35,36]. Position 2 favors hydrophobic interactions (Fig. 2C). Positions 5 and 10 interact with negatively charged residues (Glu/Asp), consistent with the polar nature identified from the crystallography analysis of HLA-DQ2 (Table S1). Position 7 can form steric interactions and hydrogen bonds, for example, with positively charged residues Arg, Lys, and His (Table S1), further convincing us that the computational results match those observed in the experimental structures [35,36].

To summarize, while antigenic epitopes bound by MHC-II exhibit no distinct sequence-level patterns in amino acid composition, highly overlapping epitope backbones have been observed in the x-ray crystal structures of various pMHC-IIs. Notably, side chains at 8 positions (1 to 6, 9, and 10) demonstrate highly spatial superimposition. Although DR-, DQ-, and DP-encoded MHC-II share multiple conserved motifs in their G-domain sequences, these motifs show varying degrees of positional shifts across different MHC-II types. Despite these shifts, the conserved motifs demonstrate spatial overlap in aligning 3D structures. Among these, 12 conserved and nonconserved residues are identified as critical determinants of epitope binding within the MHC-II binding groove. Thus, we hypothesized that 3D structural features of epitopes, rather than primary sequence patterns, are essential for identifying antigenic epitopes capable of MHC-II binding and T cell activation. Since different amino acids may form similar interactions if they have similar functional groups, maintaining or optimizing T cell activation may be achievable by fixing the epitope’s 3D conformation and designing its sequence. However, a critical prerequisite is that designed epitope sequences remain homologous to their source antigens, ensuring the induce immune response target the same pathogenic site. This will be discussed later.

### A novel MHC-II epitope structure dataset

**Fig. 3.**
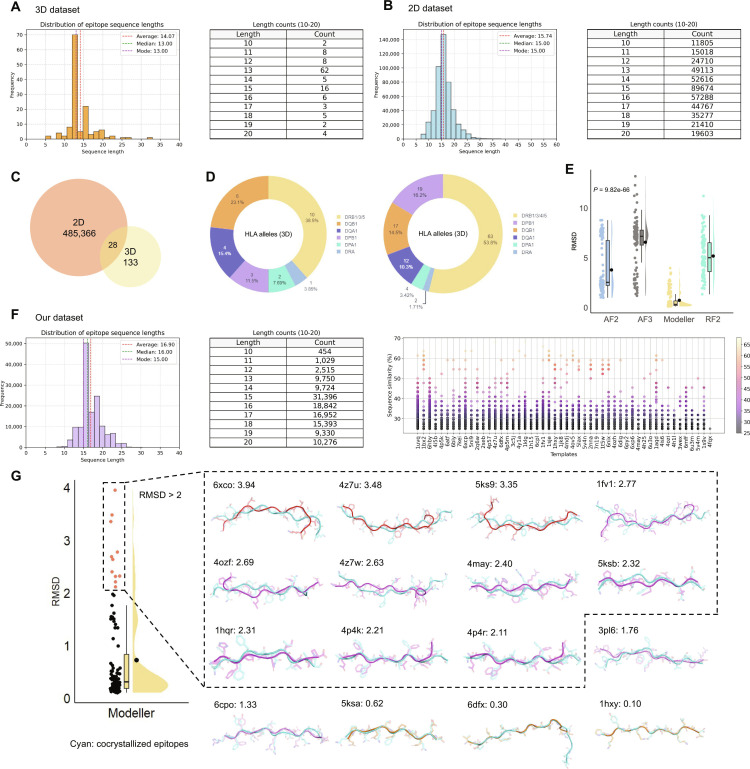
Comparison of the structure dataset, sequence dataset, and our dataset. (A) The distribution and counts of cocrystallized epitope sequence lengths of the structure dataset collected from PDB. (B) The distribution and counts of epitope sequence lengths of the sequence dataset downloaded from the Immune Epitope Database. (C) The total number of epitopes in the sequence and structure datasets. Twenty-eight cocrystallized epitopes in the structure dataset are also included in the sequence dataset. (D) The types of HLA class II alleles are covered in the structure and sequence datasets. For the sequence dataset, DRB has a total of 63 DRB variants, including DRB1 (53), DRB3 (4), DRB4 (3), and DRB5 (3). (E) The homology modeling performance of AlphaFold2 (AF2), AlphaFold3 (AF3), MODELLER, and RosettaFold2 (RF2). RMSD values are calculated between the modeled epitopes and the cocrystallized epitopes. RMSD: AF2 3.76 Å, AF3 6.55 Å, RF2 5.16 Å, MODELLER 0.71 Å. One-way ANOVA result: *P* = 9.82×10^−66^. (F) The distribution and counts of modeled epitope sequence lengths in our dataset, which has 142,934 modeled epitopes. The average length is 16.9, and the median length is 16.0. The right panel shows the scatter plot of the sequence similarities of 142,934 epitopes compared to their templates. MODELLER templates are listed in the *x*-axis. (G) The detailed modeling performance of MODELLER. The left panel shows the RMSDs between cocrystallized and MODELLER-modeled epitopes. The mean RMSD is 0.71 Å, and the median RMSD is 0.30 Å. The right panel shows the pairs of modeled/cocrystallized epitopes with different ranges of RMSD values. A total of 11 pairs have RMSD > 2 Å (magenta and red). Three pairs have RMSD > 3 Å (red). Example pairs with RMSD < 2 Å and > 1 Å (violet). Example pairs with RMSD < 1 Å (orange).

Currently, only 133 epitopes (organized as a structure dataset) that cover 35 types of antigens have ready-to-download 3D structures from PDB, which are insufficient to train a robust model (Fig. 3A). Hence, we expanded the dataset by using homology modeling techniques to model epitope sequences into 3D structures. From the Immune Epitope Database (IEDB), we obtained 485,366 epitope sequences, forming a sequence dataset covering ~20,031 antigen types (Fig. 3B). Twenty-eight cocrystallized epitopes in the structure dataset are also included in the sequence dataset (Fig. 3C). The sequence dataset encompassed a broader range of HLA class II allele types than the structure dataset (Fig. 3D). To evaluate structural modeling performance, we excluded the epitopes from the structure dataset with nonessential residues, such as citrulline (CIR), and the 1seb epitope whose sequence is composed of 13 alanine residues. From the remaining 102 cocrystallized epitopes, we evaluated 3 tools: RoseTTAFold2 (RF2) [37], AlphaFold2 (AF2) [38], and MODELLER [39]. MODELLER, a comparative (homology) protein structure modeling framework, constructs the 3D structures of a target protein using one or more experimentally determined homologous templates [40]. It applies spatial restraints derived from template-target alignments and refines models via a restrained objective function. Unlike ab initio deep learning approaches such as AlphaFold2/3 and RoseTTAFold2, which predict structures directly from sequence using neural networks trained on massive datasets, MODELLER requires high-quality templates and assumes that homologous proteins share similar folds. The RMSDs between the modeled and cocrystallized epitopes were calculated, and the results showed that epitopes modeled by MODELLER exhibit significantly lower RMSDs than those modeled by AF2/3 and RF2 (Fig. 3E and Table S2). The mean RMSD of MODELLER-Modeled epitopes is 0.71 Å, and the median RMSD is 0.30 Å, indicating an excellent homology modeling performance. Structures with RMSD <2 Å show excellent backbone/side-chain alignment (Fig. 3G), and only 11 modeled epitopes out of 102 have RMSDs > 2 Å but < 4 Å (Fig. 3G). Thus, we selected MODELLER for 3D structure prediction of epitope sequences.

To improve the dataset quality for protein design, we implemented the following sequence alignment and similarity filtering pipeline. The epitopes in the sequence dataset were aligned with the MODELLER templates (Table S3), and pairwise sequence similarities were computed. Each epitope was matched to its highest-similarity template, and we generated 3 temporary datasets by selecting epitopes with template-similarity thresholds >30%, >28%, or >25%, followed by deduplication to remove redundancy. This process aimed to identify the optimal similarity cutoff for reliable homology modeling. Three datasets of modeled epitopes were used to train our model to identify which sequence similarity of epitopes can achieve better training, validation, and test performance. The results revealed that the >25% similarity dataset achieved the lowest validation perplexity, highest validation accuracy, and less overfitting compared to the other 2 datasets (Fig. S4). We randomly selected 100 epitopes from the sequence dataset that were not included in the current dataset as a test set to calculate the sequence recovery, and found that the group with >25% sequence similarity showed markedly higher mean/median sequence recovery versus the other thresholds (Fig. S4). Based on these findings, we chose the dataset with >25% sequence similarity, which contains 142,934 nonredundant epitopes with paired sequences and MODELLER-generated 3D structures. These modeled structures exhibit >25% sequence similarity to cocrystallized reference epitopes, providing the optimal balance of structural accuracy and dataset diversity for training protein design model. This curated dataset will serve as the foundation for subsequent model development.

### EpiMII: A functional model for MHC-II epitope and neoantigen design

**Fig. 4.**
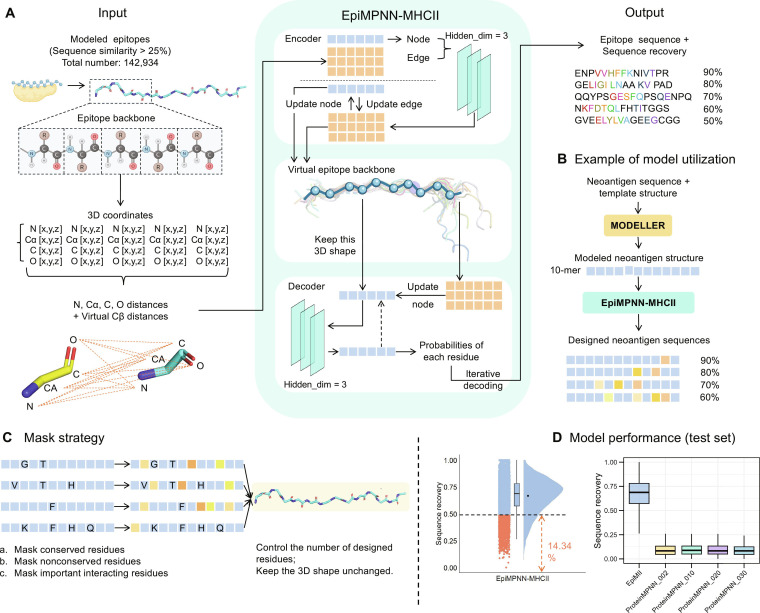
An overview of EpiMII (EpiMPNN-MHCII). (A) EpiMII takes modeled 3D epitopes as input, extracts the 3D coordinates and amino acid composition, and then calculates the distances between the same or different types of atoms, including N, Cα, C, O, and virtual Cβ. The distances are input as edges. The node is empty. The node and edges are updated through 3 hidden layers in the encoder layer. The updated node and edges form a virtual 3D epitope backbone shape in the latent space. In the decoder layer, nodes are updated based on the original amino acid composition via 3 hidden layers with a prerequisite of keeping the 3D shape. Each residue will be calculated using probabilities, and a weighted probability for the designed sequence will be generated. During the iterative decoding process, sequences are optimized to achieve better probabilities. EpiMII outputs several 2D sequences with different sequence recoveries. (B) The example of model utilization. The neoantigen will be matched with a 3D cocrystallized epitope as a template and go through homology modeling via MODELLER. EpiMII will input the modeled 3D neoantigen to design its sequence, generating designed 2D sequences in the same length with different sequence recoveries. (C) Three mask strategies to improve the low sequence recoveries of the designed peptides, including masking (non-)conserved residues and masking important interacting residues. (D) The model performance measured using a test set (14,294 modeled epitopes). The left panel shows our model’s performance. The mean sequence recovery is 0.667, and the median sequence recovery is 0.688. Red dots represent epitopes with lower than 50% sequence recoveries that only account for 14.34%. The left panel shows the performance compared with ProteinMPNN models using the test set. Panel (A) was created with BioRender.com.

EpiMPNN-MHCII (EpiMII) is constructed based on the message-passing graph neural network (MPNN), which propagates node features by exchanging information between neighboring nodes [41]. EpiMII accepts both 3D coordinates and sequences from our curated dataset as inputs and learns to generate the virtual 3D backbone shapes of epitopes by integrating 3D coordinates and amino acid composition, followed by iterative residue optimization to generate multiple sequences with varying sequence recoveries (Fig. 4A and B). To ensure that the generated model only changed a limited number of residues, we used 3 masking strategies to rescue epitope with low sequence recovery to achieve different purposes (Fig. 4C): (a) fixing conserved residues and modifying nonconserved residues to design hypervariable regions with the potential to change epitope binding specificity, (b) fixing nonconserved residues and modifying conserved residues to explore alternative core binding residues beyond inherent antigenic patterns to potentially adjust immunogenicity, and (c) masking important residues to customize the design positions based on crystallographic insights or literature-reported key residues. Modeling performance was evaluated using the test set, which contains 14,294 modeled epitopes (Fig. 4D). The mean sequence recovery is 66.7%. Among them, only 14.34% of epitopes in the test set have sequence recoveries lower than 0.5, indicating that most of the designed sequences have higher than 50% sequence identity compared to the original input sequence (Fig. 4D). Backbone perturbations (0 to 0.9 Å) degraded performance (↑perplexity, ↓accuracy to ~0.5) and induced overfitting; across 102 modeled epitopes, the noise-free model achieved the best sequence recovery (0.78), so we selected an early-stopped model (epoch 50, step 47,050; Fig. S8). We compared EpiMII to 4 ProteinMPNN models (v_48_002, v_48_010, v_48_020, and v_48_030) using an identical test set. The results demonstrated that our model consistently outperformed all ProteinMPNN variants in MHC-II epitope design (Fig. 4D). Across 102 matched epitopes, per-position log-probabilities (log*P*) from the EpiMII decoder were nearly identical between MODELLER-built and crystal structures (Pearson *r* = 0.9842), indicating robustness to minor conformational differences (Fig. S9).

**Fig. 5.**
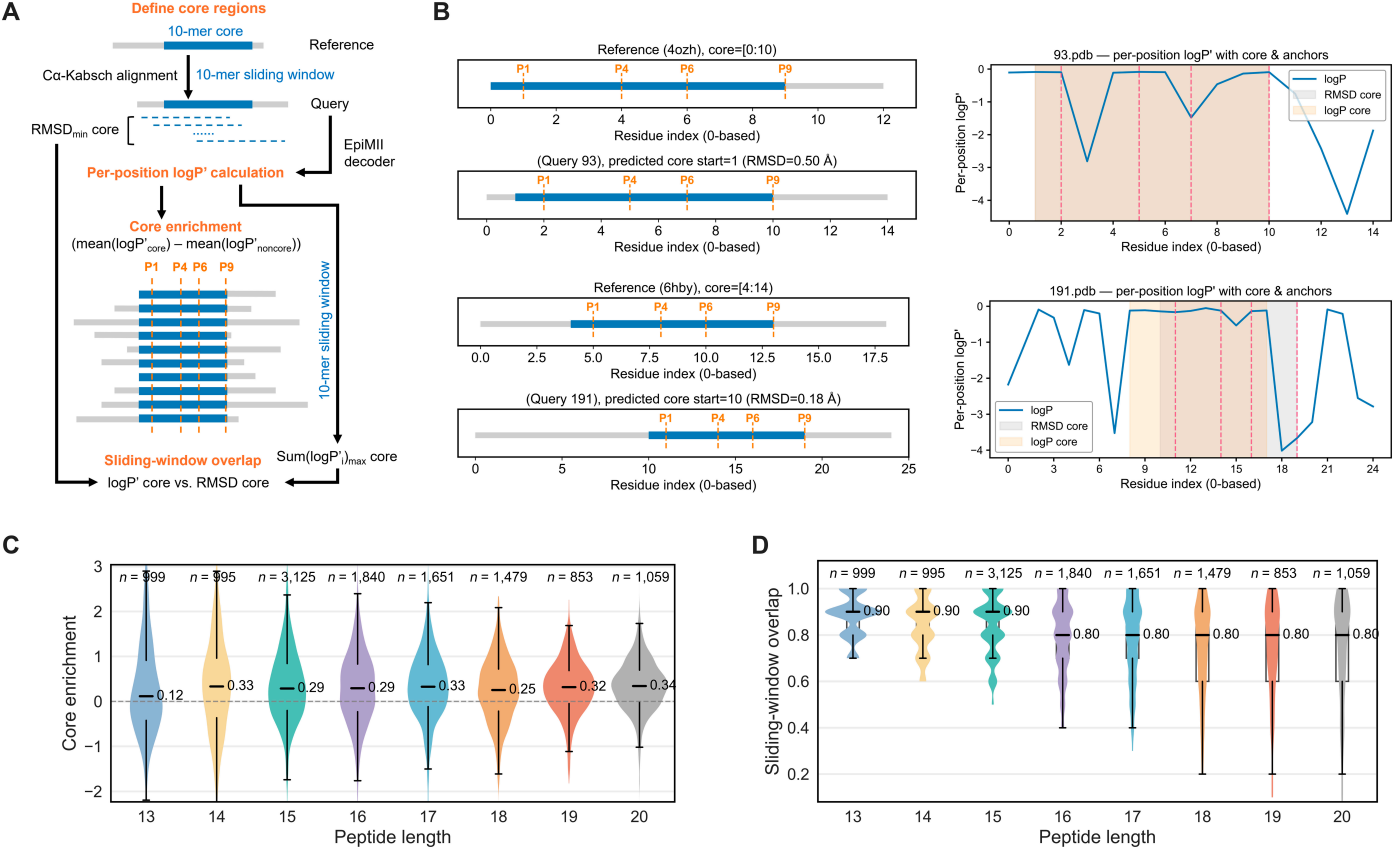
(A) The overview of the analysis workflow for panels (B) to (D). (B) Representative examples of epitopes 93 and 191. Left: Reference template and aligned core (blue). Right: Per-position log*P*′ profile of the Query epitope and the overlap between RMSD core and log*P*′ core. Red dashed lines indicate the corresponding anchor positions P1, 4, 6, and 9 from left to right putative from the log*P*′ core. (C) Distribution of core enrichment across peptide lengths; medians are annotated as the short solid lines in the middle of the violin-box plot. Violin plots show the full distribution with embedded boxplots. Sample sizes for each length group are annotated on the top. (D) Distribution of sliding-window overlaps between decoder-derived log*P*′ and RMSD-defined cores across peptide lengths; medians are annotated as the short solid lines in the middle of the violin-box plot.

To evaluate whether EpiMII assigns higher likelihoods to structurally defined binding cores identified in the crystal structures (Fig. 1F), we analyzed core enrichment and sliding-window overlap across 14,293 MHC-II epitope structures in the test set (Fig. 5A). For each query peptide, a reference crystal structure was matched to define a 10-mer binding core via Cα-based Kabsch alignment, which identifies the reference binding core (RMSD core) by minimizing the RMSD of corresponding Cα atoms through rigid-body transformation [42]. Per-position log*P*′ values (distinct from log*P*) were computed in test mode with masking and sampling, providing residue-level model confidence along the peptide. A 10-mer sliding window was applied to identify a log*P*′ core (Fig. 5A). Representative examples from queries 93 and 191 in the test set showed that peaks in per-position log*P*′ values occur within the structurally aligned core, covering most canonical anchor positions (P1, P4, P6, and P9), while flanking regions exhibit lower likelihoods. However, the overlap between RMSD core and log*P*′ core varied across peptides (Fig. 5B).

We quantified core enrichment as the difference between average log*P*′ values inside versus outside the 10-mer core. This metric was consistently positive across peptide lengths, with median enrichment values ranging from ~0.12 to ~0.34 log units, indicating that the model preferentially concentrates probability in regions interacting with the MHC-II binding groove (Fig. 5C). Further comparison of RMSD core with log*P*′ core, which was determined as the 10-mer segment with the highest summed log*P*′ values, revealed high overlap, with medians from ~0.80 to ~0.90 across peptide lengths. This supports strong concordance between decoder-derived likelihoods and structural alignment (Fig. 5D). Together, these results demonstrate that EpiMII learns residue-level likelihood patterns that accurately delineate the binding cores of epitopes that interact with the MHC-II binding grooves, capturing essential features of epitope residues involved in peptide–MHC-II interactions.

### EpiMII preserves MHC-II binding in a label-free setting

**Fig. 6.**
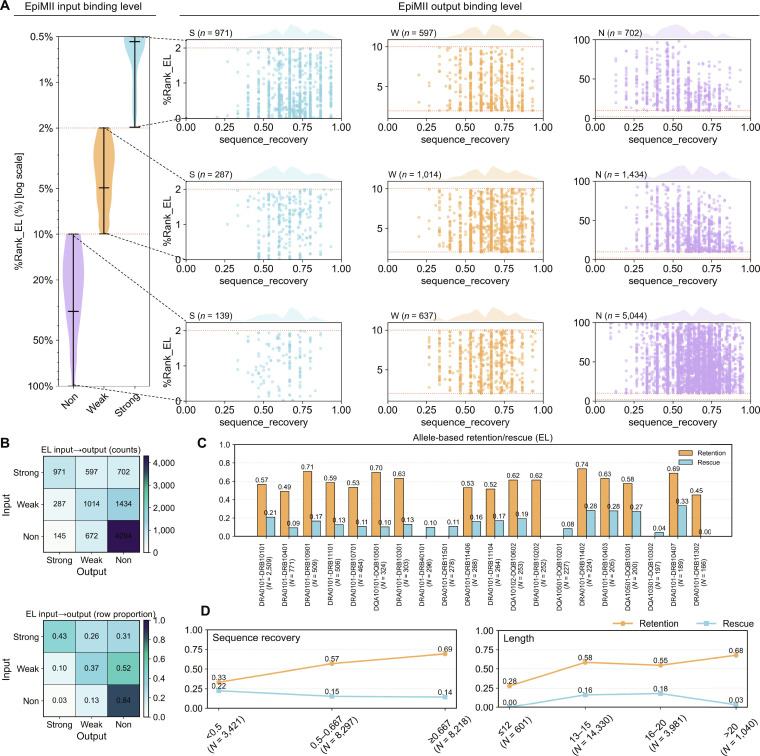
The evaluation of whether EpiMII preserves MHC-II binding using %Rank_EL as the primary endpoint. (A) The overview of Input→Output flows (counts). The left panel is the input bin, which contains 1,771 T cell-positive epitopes that are divided into strong binders (%Rank_EL < 2), weak binders (2 ≤ %Rank_EL < 10), and nonbinders (%Rank_EL ≤ 10) as predicted by NetMHCIIpan-4.3. The right panel shows the outputs from EpiMII with sequence recovery per sequence and NetMHCIIpan4.3-predicted %Rank_EL score. The NetMHCIIpan-4.3-predicted positive-binding epitopes in (A) and predicted negative-binding epitopes in the T cell-negative set are used for analysis in (B), (C), and (D). (B) The heatmap of the counts and row-normalized proportions. (C) Allele-level Retention and Rescue (top 20 MHC-II types by sample size, sample size *N* ≥ 30). The *y*-axis shows the proportion. Group means are at the top of each bar. (D) Left: Retention/Rescue versus sequence recovery of the designed output (bins: <0.5, 0.5 to 0.667, and ≥0.667). The *y*-axis shows the proportion. Right: Retention/Rescue versus peptide length (≤12, 13 to 15, 16 to 20, and >20 aa).

To evaluate whether EpiMII, trained without MHC-II allele labels, preserves peptide–MHC-II binding when transforming input sequences into outputs, we curated 1,771 T cell-positive and 1,739 T cell-negative epitopes from the IEDB that were absent from the training set. Each input sequence was processed to generate 8 outputs by EpiMII without masking, followed by deduplication. For every input/output–allele pair, binding was scored using NetMHCIIpan-4.3 [15], with %Rank_EL as the primary endpoint (lower is better; Strong <2%, Weak 2% to 10%, Non ≥10%). We quantified 2 probabilities from paired data: Retention = *P*(Output <10% | Input <10%) and Rescue = *P*(Output <10% | Input ≥10%). We also summarized input–output flows and examined how Retention/Rescue varies with sequence recovery and peptide length (Fig. 6). Across the entire dataset, inputs predicted as Strong binders were often maintained as binders: 43% remained Strong, 26% became Weak, and 31% became nonbinders. Weak binders were more likely to lose binding than improve (10% transitioned to Strong, 37% remained Weak, and 52% became Non). Nonbinders were rarely rescued (3% to Strong, 13% to Weak, and 84% remained Non) (Fig. 6A and B). Aggregating across EL pairs, overall binder Retention was 0.574 (2,869/5,005), decomposed as 0.691 for Strong inputs (1,568/2,270) and 0.476 for Weak inputs (1,301/2,735). Rescue from nonbinders was 0.160 (817/5,111). These results demonstrate that EpiMII more effectively preserves existing binding, particularly for Strong inputs, than converts nonbinders into binders, highlighting its capacity to maintain functional peptide–MHC-II interactions despite the absence of allele-specific training.

Retention exhibited variability across MHC-II alleles but typically ranged from 0.5 to 0.70, while Rescue values remained lower at 0.1 to 0.28, consistent with allele-specific anchor constraints (conserved residues on the MHC-II binding groove), which implicitly shapes the feasible design space even in the absence of explicit allele labels (Figs. 2C and 6C). Higher sequence recovery was associated with monotonic increases in Retention (from 0.33 at recovery <0.5 to 0.69 at ≥0.667) and a modest decline in Rescue (0.22 to 0.14), suggesting that conserving input features, particularly anchor residues, supports binding maintenance, whereas successful rescue typically requires larger sequence edits (Fig. 6D). However, larger edits usually come with the risk of disrupting T cell specificity. Peptide length analysis showed that 16- to 20-mers achieved high Retention with modest Rescue (0.18). Shorter peptides (≤12-mers) showed negligible Rescue, and longer peptides (>20-mers) retained binding well but were rarely rescued. The 13- to 15-mer range balanced Retention (0.58) and Rescue (0.16) with a relatively large number of samples (Fig. 6D).

Analyses with %Rank_BA recapitulated the EL findings: Weak inputs predominantly transitioned to Non; the overall Retention was 0.626 (0.699 for Strong, 0.535 for Weak) and Rescue reached 0.214; and the effects of sequence recovery and peptide length were similar (Fig. S5). Together, these results demonstrate that in a label-free regime, EpiMII reliably preserves strong binders, with sequence conservation and 13- to 15-mer lengths being critical to maintain MHC-II binding. Rescuing nonbinders remains inherently challenging, likely requiring targeted, larger sequence modification balanced against preserving T cell specificity.

### Designing a reported neoantigen for HCC using EpiMII

**Fig. 7.**
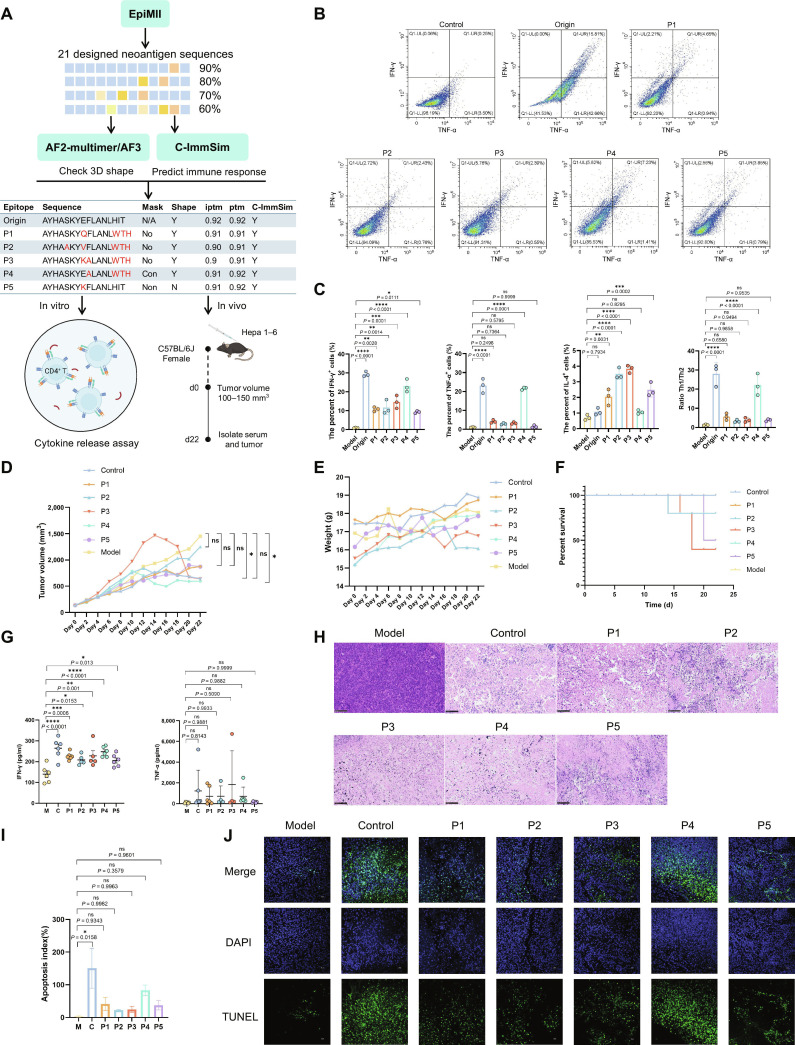
In vitro and in vivo experimental validations of designed HCC neoantigen candidates. (A) An overview of the whole process of candidate selection and experimental validations. “Y” means the peptide is predicted to trigger the CD4^+^ T cell immune response. (B) Trends of IFN-γ and TNF-α contents in each group. IFN-γ detected by PE; TNF-α detected by PC7. (C) IFN-γ, TNF-α, IL-4 contents (Fig. S6), and Th1/Th2 ratio in cells of each group. (D) Comparison of tumor volume size in mice in 0 to 22 days. C vs. M: *P* = 0.0376 (*). P1 vs. M: *P* = 0.1221 (ns). P2 vs. M: *P* = 0.9961 (ns). P3 vs. M: *P* = 0.9105 (ns). P4 vs. M: *P* = 0.0189 (*). P5 vs. M: *P* = 0.1560 (ns). (E) Body weight of mice from 0 to 22 days. (F) Survival status of mice in 0 to 22 days. The survival rate of P2 and P4 mice was 80%, that of P5 was 50%, that of P3 was 40%, and that of the other groups was 100%. (G) Comparison of IFN-γ and TNF-α levels in mouse serum. (H) HE staining of tumor sections in each group (original magnification: ×40). The tumor cells in the Model group had normal volume, appearing round or oval, and had clear cytoplasm staining, clear nucleoli and nuclear membrane staining, and clear staining resolution. The tumor cells of the Control and P1 to P5 groups showed marked shrinkage, low chromatin clarity, and apparent shrinkage of the nucleolus and nuclear membrane. The nuclear envelope details are blurred. The coloring hierarchy and contrast are not clear enough. (I) Quantitative analysis of TUNEL. (J) TUNEL staining of tumor sections in each group (400×). DAPI-stained nuclei were blue at 405 nm excitation wavelength, the TUNEL kit was labeled with green fluorescein, and positive apoptotic nuclei were green with 488 nm excitation wavelength. Panel (A) was created with BioRender.com.

HCC, which accounts for over 90% of primary liver tumor, is one of the most prevalent cancers globally [43]. The 5-year survival rate for HCC patients is only 18% [44,45]. In a real-world case study, we applied EpiMII to design AYHASKYEFLANLHIT, a reported HCC neoantigen derived from a single point mutation of the wild-type protein encoded by the gene DUSP5 [46]. Source from the Neoantigen database (Neodb: https://github.com/XSLiuLab/Neodb) and absent from our training dataset, this neoantigen was previously reported to bind to HLA-DRB10101-encoded MHC-II [47] . From the original neoantigen AYHASKYEFLANLHIT (denoted “Origin”), EpiMII generated 21 designed sequences with varying sequence recoveries using 4 masking strategies: no masking, masking conserved residues, masking nonconserved residues, and masking important interacting residues (Fig. 7A and Table S4). We then predicted the 3D structures of all designed sequences via the AF2-multimer and AlphaFold3 (AF3) to identify candidates with similar 3D shapes as we previously characterized. For all 6 candidate-G complexes (G-domain of DRA*01:01-DRB1*01:01), predicted TM-score (ptm) and interface predicted TM-score (iptm) scores showed minimal variation (Fig. 7A and Table S4). C-ImmSim was also applied to all designed sequences to computationally evaluate their ability to induce CD4^+^ T cell immune response [48]. Finally, 5 designed sequences that cover different masking strategies and sequence recovery levels were selected for in vitro and in vivo experimental validations (Fig. 7A and Table S4).

For in vitro validation, human peripheral blood mononuclear cells (PBMCs) were obtained from a healthy donor with the DRB10101 genotype. The immune response was stimulated by culturing PBMCs with HLA-DRA0101 and HLA-DRB10101 genotypes in vitro. The CD3^+^CD4^+^ T cells were first screened out by fluorescein isothiocyanate (FITC) and PerCP/Cyanine 5.5 labeling. Then, the intracellular factor fluorescence staining of PE, PC-7, and APC was detected. The results showed that Origin and P4 groups had the most potent ability to activate T cells, and the increasing trends of IFN-γ and TNF-α contents reached 15.81% and 7.23%, respectively (Fig. 7B). P1 and P5 showed moderate responses (4.65% and 3.85%), while P2 and P3 displayed minimal activation (2.43% and 2.39%). Notably, P4 induced the most marked immune activation, comparable to the Origin neoantigen in human CD4^+^ T cells. The secretion of IL-4 was almost negatively correlated with that of IFN-γ: Origin and P4 secreted the least IL-4 compared to the other groups, while P2, P3, and P5 had more IL-4 secretion compared with Origin, P1, and P4 (Fig. 7C and Fig. S6). IFN-γ is the surface marker of Th1 cells, and IL-4 is the surface marker of Th2 cells. According to the secretion of IFN-γ and IL-4, Th1/Th2 can be obtained. In all groups, only Origin and P4 showed excellent significant differences (Fig. 7C). These findings confirm that P4 effectively stimulates CD4^+^ T cells to secrete high levels of IFN-γ and TNF-α while promoting Th1 differentiation. It is preliminarily inferred that P4 can effectively activate CD4^+^ T cells, warranting further in vivo validation.

In vivo validation was conducted on C57BL/6 mice. Mice treated with Origin neoantigen were set as the positive control group (Control). After 0 to 22 days of epitope treatments, the peptides with the most significant tumor inhibition effect are Control and P4 groups with *P* < 0.5, as shown in Fig. 7D and Fig. S7. P3 also showed an inhibitory effect in the later stage, but this is likely due to the error caused by the increased mortality of this group of mice in the later stage. Compared with the Model group, P1 and P5 also slowed down tumor volume growth (Fig. 7D and Fig. S7). No marked weight loss occurred across treatment groups over 22 days (Fig. 7E), and survival rates are presented in Fig. 7F. Enzyme-linked immunosorbent assay (ELISA) measurements of mouse serum revealed that all treatment groups (Control and P1 to P5) had significantly higher IFN-γ concentrations than the Model group, with Control and P4 showing the highest levels (*P* < 0.0001) (Fig. 7G). The differences in the secretion of TNF-α are mainly reflected at the individual level: The TNF-α levels of some individuals in the Control and P3 groups have increased significantly; some individuals in P1, P2, and P4 have also increased to some extent, with P5 being the lowest (Fig. 7G). The tumor cells on the HE-stained sections of the treatment groups (C and P1 to P5) showed marked shrinkage, low chromatin clarity, and apparent shrinkage of the nucleolus and nuclear membrane compared to the tumor cells in the Model group. Control and P4 are the most apparent (Fig. 7H). Terminal deoxynucleotidyl transferase dUTP nick end labeling (TUNEL) staining is consistent with the results of HE staining, showing that the apoptosis of control and P4 tumor cells was the most serious, and there was no significant difference in other groups (Fig. 7I and J). This further confirmed the marked tumor-inhibitory effects of P4 compared to mice without any neoantigen treatment and the parallel effects compared to the positive control.

### Neoantigen-specific CD4^+^ T cell responses and TCR repertoire analysis

To evaluate the immunogenicity of the WT peptide (sequence: AYHASKCEFLANLHIT) and the “origin” neoantigen peptide (MUT peptide, sequence: AYHASKYEFLANLHIT), we measured the frequencies of IFN-γ^+^, TNF-α^+^, and IL-4^+^ CD4^+^ T cells after peptide stimulation. In the negative control group (no peptide restimulation, no PMA/BFA), background levels of IFN-γ^+^ and TNF-α^+^ cells were observed, consistent with IL-2-driven basal activation. IL-4^+^ cells were nearly undetectable (Fig. S10A). In the positive control group (WT peptide + PMA/BFA), the frequencies of IFN-γ^+^ and TNF-α^+^ CD4^+^ T cells were substantially increased compared to the negative control, with IL-4^+^ cells accounting for approximately 2.9% (Fig. S10B). Following WT peptide stimulation, 39.05% of CD4^+^ T cells were IFN-γ^+^ and 39.30% were TNF-α^+^, while IL-4^+^ cells remained negligible (Fig. S10C). Following MUT peptide stimulation, the frequencies of IFN-γ^+^ and TNF-α^+^ CD4^+^ T cells were 58.01% and 58.13%, respectively, indicating a stronger Th1-type response compared to WT peptide. IL-4^+^ cells were also barely detectable in this group (Fig. S10D). These results demonstrate that both WT peptide and MUT peptide are capable of inducing antigen-specific CD4^+^ T cell responses, with MUT peptide eliciting a more potent Th1-type cytokine profile.

Analysis of the cell clusterings in PBMCs revealed that after stimulation with WT peptide and MUT peptide, the PBMCs were mainly composed of CD3^+^ T cells, including CD4^+^ T cells and CD8^+^ T cells, as well as a small number of NK cells and macrophages (Fig. S11A and B). By analyzing the expression of relevant genes in the MUT-peptide-stimulated group and the WT-peptide-stimulated group, the activated CD4^+^ T cell clusters (defined as CD3E^+^, CD4^+^, CD8A^−^, and IFNG^+^) were identified among the CD4-positive cell populations: clusters 4 and 7 (Fig. S12A). Analysis results showed that clusters 4 and 7 expressed T cell markers and activation markers (activation markers: CD40L and TBX2; cytokines: IFNG, TNFa, and IL4; perforin: PRF1; granzyme B: GZMB) (Fig. S12B and C). Meanwhile, clusters 4 and 7 also expressed markers associated with T cell exhaustion caused by long-term culture (KLRF1 and PDCD1). Subsequently, TCR analysis was performed on the selected clusters 4 and 7.

After integrating the single-cell transcriptome sequencing (scRNA-seq)-defined activated CD4^+^ T cells with the TCR sequencing (scTCR-seq) data, the results are shown in Tables S8 and S9. In the context of tumor vaccines, peptides that activate a broader repertoire of TCR sequences (high diversity) are generally superior to those activating only a few TCR clones, as this provides more robust antitumor immune protection, reduces the risk of immune escape, and is associated with better clinical prognosis. Based on this, the present study screened and analyzed the TCR sequences corresponding to T cell clones shared after WT peptide and MUT peptide stimulation, aiming to identify T cell clone sequences capable of generating specific responses upon stimulation with both antigens. Two clones were identified through this screening, and the results are shown in Table S10.

The final target protein concentration was 0.38 mg/ml in a volume of 3.81 ml, yielding a total of 1.44 mg of protein from a 100-ml expression system. Reducing sodium dodecyl sulfate–polyacrylamide gel electrophoresis (SDS-PAGE) (Fig. S13A) showed a single band, indicating successful protein expression. Nonreducing SDS-PAGE revealed multiple bands, with a faint band corresponding to the target protein at 104 kDa, suggesting a low yield of correctly assembled protein. Size-exclusion chromatography high-performance liquid chromatography (SEC-HPLC) (Fig. S13B) analysis showed major peaks at retention times of 6.1 and 6.4 min, indicating that the protein predominantly existed in aggregated forms.

We selected the TCR sequence from Clone 1 in Table S10 for synthesis, expression, and purification. We then used surface plasmon resonance (SPR) technology to measure the affinity of the TCR–HLA–peptide complex. The TCR was coated onto a CM5 chip, and the HLA–peptide complex was used as the flowing analyte. The results showed that the HLA–WT peptide (sequence: AYHASKCEFLANLHIT) exhibited high levels of nonspecific binding, and its actual *K*_D_ value could not be fitted (Fig. S13C). Although the HLA–MUT peptide (sequence: AYHASKYEFLANLHIT) and HLA–peptide 3 (P4, sequence: AYHASKYEALANLWTH) complexes exhibited low nonspecific binding to the chip, the relative response (RU) remained negative, and the software was unable to fit the *K*_D_ values (Fig. S13D and E).

Based on these results, we performed single-cell sequencing of PBMCs following peptide stimulation once again. This time, we conducted stimulation in 3 groups simultaneously: the MUT peptide, WT peptide, and P4 peptide stimulation groups. By integrating scRNA-seq and scTCR-seq data, we compared the gene expression profiles and TCR clonal characteristics of activated CD4^+^ T cells across the MUT peptide, WT peptide, and P4 peptide stimulation groups. We compared the gene expression profiles and TCR clonal characteristics of activated CD4^+^ T cells across the MUT peptide, WT peptide, and P4 peptide stimulation groups. Sequence alignment results showed that Clone 1 was present in all 3 stimulation groups, and its single-cell TCR sequences (including α and β chains) were fully consistent with the previously identified Clone 1, confirming its cross-reactivity. Detailed TCR information for T cell clones obtained from the P4 peptide stimulation is shown in Table S11.

To verify the specific binding capacity of Clone 1 TCR to the target antigen, we are currently conducting in vitro binding assays between HLA–peptide complexes and the TCR. These assays will also determine the recognition specificity and binding affinity of this TCR clone toward the target HLA–peptide complex. The specific experimental approach is as follows: First, we will establish an in vitro assembly system for HLA and peptides (WT peptide, MUT peptide, and P4 peptide), prepare high-purity HLA–peptide complexes, and biotin-label the complexes to meet the requirements of subsequent binding assays; Second, we will perform in vitro expression and purification of Clone 1 TCR heterodimers to obtain sufficient quantities of highly active TCR protein; finally, we will use SPR in vitro binding assays to detect the binding of Clone 1 TCR to different HLA–peptide complexes (WT peptide–HLA, MUT peptide–HLA, and P4 peptide–HLA). By quantifying the binding signals, we will determine the specific recognition capacity of Clone 1 TCR toward target antigen peptides, further elucidate the mechanism of action of this TCR clone in antitumor immune responses, and compare the binding profiles of the P4 peptide with those of the native epitope peptides.

## Discussion

This study aimed to investigate the critical roles and potential utilizations of 3D structural features of antigenic MHC-II mimotopes during identification. Through the thorough structure- and sequence-based analysis of MHC-II and epitopes, we created the first novel 3D MHC-II epitope dataset using homology modeling techniques to overcome the structure data insufficiency, making the structural features available in the deep learning area. Our model EpiMII achieved a 4.2× improvement in the sequence recovery of the existing 102 MHC-II epitopes derived from PDB. Using a reported HCC neoantigen as the case study, EpiMII successfully generated 5 mimotopes demonstrating their ability to activate CD4^+^ T cells in vitro. One candidate, P4, outperformed the others regarding parallel immunogenicity and tumor shrinkage compared with the reported neoantigen in vivo.

These results indicate that the GNN-based model, EpiMII, can learn the relationship between the MHC-II epitope sequences and their 3D structural information to generate sequences that potentially keep the same function. Combining the experimental results with model outputs, we found that the sequences with fewer mutated residues did not necessarily exhibit better immunogenicity. In other words, higher sequence recovery does not guarantee superior immunogenicity. The position of the changing residue matters the most, as shown in Fig. 7A. P4 has a lower sequence recovery and more changed residues than P5, while P4 performed better than P5. We generated P4 using the “masking all conserved residues strategy” in EpiMII by masking the residues at positions 1 to 6, 9, and 10 (Table S4). Compared to “no mask residues” and “mask nonconserved residues” strategies, we speculate that masking conserved residues can keep the most critical interactions unchanged between MHC-II and epitope and keep the random-coil shape of the epitope backbone at the same time. This possibly guarantees that the designed epitope will target the same site. Similar findings also appear in some previous researches about MHC-I mimotope identification, which proposed that the mimotopes with amino acid substitutions on nonanchor positions but not anchor positions had considerably better response compared to their wild-type epitopes in clinical applications [49–51]. However, the conserved 3D shape of epitopes and designed mimotopes cannot be captured by AF2 or AF3 since the predicted structures have almost the same iptm and ptm scores as the input neoantigen (Fig. 7A). The results of MHC-II epitope analysis align with the reverse-binding mode of DP–epitope complexes identified by Racle et al. [16]. Still, our dataset did not show MHC-II binding motifs similar to those discovered by MoDec. This discrepancy may be due to differences in the types of antigens covered in the datasets and the strategies for finding the motif. We tried identifying the residue frequencies at every position for all epitopes that bind to the same MHC-II types. Racle et al. [16] discovered several binding motifs shared by most of the epitopes in the same group. The results do not conflict.

Rationally designed CD4^+^ T cell mimotopes can potentiate helper responses against weak tumors or pathogen epitopes, broaden recognition of antigenic variants, or modulate T cell function through altered peptide ligands [31,52]. In addition, MHC-II mimotopes may provide sensitive tools for epitope mapping and immune monitoring [53,54]. Thus, the ability of EpiMII to systematically design MHC-II mimotopes extends the utility of epitope prediction toward downstream vaccine and immunotherapy development. Our design model applies not only to MHC-II mimotopes, but its application also extends to cancer immunotherapy, particularly in therapeutic T cell cancer vaccine. EpiMII makes it possible to rescue or improve the epitopes that triggered insufficient T cell immune response by optimizing specific residues on the epitope’s sequence to adjust immunogenicity and respond to the same target, for example, the same tumor site. Utilizing the conserved and nonconserved residues discovered in 66 types of MHC-II, EpiMII may increase the number of neoantigen candidates targeting the same site with different binding specificities to advance MHC-II-neoantigen-based cancer immunotherapy from the personal level to the population level. Meanwhile, EpiMII is much more efficient in lead identification than the random mutation scheme. For example, P5 was designed with one residue changed under the masking strategy, and the immunogenicity was maintained. The random mutation scheme that only changes one residue requires up to 19^*N^ mutations to achieve the same effect (*N* is the number of nonconserved residues). Not to mention multisite mutations, the number of random mutation experiments increases exponentially (20^*N^), while EpiMII can efficiently complete it with just one design, such as P1 to P4, showing great potential to promote the MHC-II neoantigen identification process.

Several factors may have influenced model performance and interpretability, as discussed below. The limited availability of cocrystallized MHC-II epitopes in the PDB restricted the size and diversity of our structure dataset while maintaining <25% sequence similarity. Despite early stopping, EpiMII showed signs of overfitting, likely due to training exclusively on high-immunogenicity epitopes, overrepresentation of certain motif types, and mapping hundreds of thousands of sequences to only ~100 structures, leading to structural redundancy and potential data-induced bias. The underrepresentation of MHC-II peptide conformational flexibility in static crystal structures may further limit generalization. EpiMII’s current sequence reconstruction objective does not explicitly preserve or model immune response intensity, making its behavior with inputs of varying immunogenicity (e.g., strong vs. weak binders) difficult to interpret. This reflects the scarcity of well-characterized weak or nonimmunogenic epitopes in structural databases, which biases the learned representation toward strong binders. Future improvements could include incorporating balanced positive and negative examples, expanding the structural repertoire across diverse HLA class II alleles, applying data augmentation to reduce redundancy, and integrating quantitative immunogenicity data in a multitask learning framework to enhance interpretability. Broader case studies, including diverse disease contexts, and exploration of nonconserved MHC-II G-domain residues may further refine mimotope design, enabling the generation of mimotopes with enhanced CD4^+^ T cell immunogenicity.

In conclusion, this study highlights the essential role of 3D structural features in the MHC-II epitope identification process. It provides a novel 3D MHC-II epitope dataset that can train deep-learning models. This overcomes constraints posed by the limited 3D epitope data and addresses the epitope candidate’s lack of immunogenicity in vivo. EpiMII paved a new direction for designing MHC-II epitopes and could facilitate neoantigen identification, providing essential insights for precise epitope design and cancer vaccine development.

## Methods

### HLA class II alleles selection

We initially figured out the frequencies of HLA class II alleles (http://pypop.org/popdata/2008/byfreq-DP.php.html), which derived from a meta-analytic review of 497 population studies and represent approximately 66,800 individuals throughout the world as shown in Table S5 [55]. Then, we calculated the HLA class II alleles population frequencies using Hardy–Weinberg proportions for genotypes. The cumulative phenotypic frequency (CPF) of the alleles was calculated using: $\mathrm{CPF}=1-{\left(1-\sum_{i\in c}\mathrm{pi}\right)}^{2}$, where *pi* is the population frequency of the *i*th alleles within a subset of HLA-DR, HLA-DP, or HLA-DQ. The top 21 HLA-DRB1 alleles cover more than 95.54% (from DRB1*1501 to DRB1*1601), and the top 31 cover more than 99.14% of the population. The top 4 HLA-DQA1 alleles cover more than 94.9% (from DQA1*0501 to DQA1*0101), and the top 6 alleles cover more than 99.55% of the population. The top 7 HLA-DQB1 alleles cover more than 94.8% (from DQB1*0301 to DQB1*0402), and the top 16 alleles cover more than 98.5%. The top 2 HLA-DPA1 alleles cover more than 95.37%, and the top 3 alleles cover more than 99.7% of the population. The top 6 HLA-DPB1 alleles cover more than 96.0% (from DPB1*0401 to DPB1*0301), and the top 9 alleles cover more than 99.0% of the population. We chose the HLA class II alleles that cover approximately more than 99% of the global population worldwide as our samples to analyze the different types of MHC-II (plus the HLA-DRA allele), which in total are 66 alleles, including DRA (1), DRB1 (31), DQA1 (6), DQB1 (16), DPA1 (3), and DPB1 (9). Then, we collected the 66 HLA class II alleles’ sequences from HLA Nomenclature (https://hla.alleles.org/pages/nomenclature/naming_alleles/), accessed 2022 October 9.

### G-domain lengths determination

G-domain is the International ImMunoGeneTics Information System (IMGT) (http://www.imgt.org) unique numbering for the MHC binding grooves. For MHC-II, it is the peptide binding domain composed of the α1 and β1 regions of the chains. By searching IMGT using “PDB” as the IMGT entry type and “MH2’ as the IMGT receptor type, we found 205 entries containing MHC-II protein. We then analyzed 11 IMGT entries covering different kinds of MHC-II, including DR, DQ, and DP. We found that the lengths of the G-domain vary not only from the alpha and beta chains but also from different types of MHC-II, as shown in Table S6. For further study, we established the general length of the G-domain, with G-alpha spanning from sequence region 1 to 84 and G-beta from sequence region 1 to 93.

### Pairwise Euclidean distances and pairwise correlation

After we obtained the protein sequences of 66 HLA class II alleles, we selected 26 mutation points for the G-alpha sequence and 50 for the G-beta sequence to represent the MHC-II binding groove and then calculated the 10 Kidera Factors for them. The 10 Kidera Factors describe multiple properties of the protein sequence [56]. The 10 Kidera Factors include the following: KF1: Helix/bend preference; KF2: Side-chain size; KF3: Extended structure preference; KF4: Hydrophobicity; KF5: Double-bend preference; KF6: Partial specific volume; KF7: Flat extended preference; KF8: Occurrence in alpha region; KF9: pK-C; and KF10: Surrounding hydrophobicity [56]. We calculated the Euclidean distance of the 10 Kidera Factors for 66 G-alpha domains and 66 G-beta domains and finally generated a 132*132 correlation matrix, as shown in Fig. 1A.

We then searched for the reported 15-mer epitopes bound to different MHC-II types on the IEDB (https://www.iedb.org/). We chose “Human” as Host, and because in our 66 HLA alleles, the alleles encoded for the alpha chain are the minority compared to the other encoded for the beta chain, we selected the “MHC Restriction” to be the DxB alleles in our 66 HLA class II alleles. The detailed numbers of selected epitopes for each DxB allele are shown in Table S7. We then measured each DxB group’s Pearson correlation and generated a 51*51 matrix, as shown in Fig. 1A.

We collected the epitopes for HLA alleles with >0.9 sequence similarities and removed the repeated epitopes because some epitopes are reported to bind to several HLA alleles. The sequence logo plots were generated for the 8 HLA alleles groups. Then, we tried to discover the potential motifs in each allele group using MEME [33]. MEME is an online tool to discover novel, ungapped motifs in the sequences (https://meme-suite.org/meme/tools/meme). We chose the “Classic mode” and requested 10 motifs to find.

### MCCS scoring technique

MCCS is a novel characterization method for the protein–ligand complex, which includes scoring and docking techniques [57]. Here, we employed the scoring technique to characterize the binding features of by quantitating the pMHC-IIs energy contribution of each individual residue [57]. Our inputs were 133 pMHC-IIs derived from x-ray crystallography. Chimera (version 1.15) was first used to fix residues with an incomplete side chain in MHC-II PDB structures [58]. The uncompleted residues were revealed after Chimera scanned the complete protein structures. The Dunbrack rotamer library was used to replace the truncated side chains with a whole side chain of the same type of residue [59]. The polar hydrogens, Gasteiger charges, and Vina force field were added using VEGA. Finally, the PDB format of both MHC-II and epitope was converted to PDBQT format for further scoring function via jdock (version 2.2.3b, https://github.com/stcmz/jdock accessed on 2024 March 20).

Jdock is a core implementation of MCCS and is an extended variant of idock, which is used in scoring and docking functions [60]. It can generate a vector of residue-free energy from the conformation determined by the x-ray crystal structure in 5 terms: gauss1, gauss2, repulsion, hydrophobic, and h-bonding, which AutoDock Vina invented [61,62]. The scoring results were 9 binding recognition vectors: (a) Gauss (Gauss1 + Gauss2), (b) Gauss1, (c) Gauss2, (d) repulsion, (e) steric (Gauss1 + Gauss2 + repulsion), (f) hydrogen-bonding, (g) hydrophobic, (h) nonsteric (hydrogen-bonding + hydrophobic), and (i) residue energy contribution. We chose the “score only” mode for performing the scoring technique in which the scores of all receptor–ligand atom pairs were directly calculated and added to the overall score.

### Structure dataset

We searched the IMGT using “PDB” as the IMGT entry type and “MH2” as the IMGT receptor type, and we found 205 entries containing MHC-II protein. We then excluded the complexes that the bound peptide is a class II-associated invariant chain peptide (CLIP), which blocks the antigen binding region of the MHC-II before the epitope comes. We finally obtained 133 pMHC-II and downloaded them from the PDB. Their epitopes were organized as a structure dataset containing 29 modified epitopes and 23 mutant epitopes.

### Sequence dataset

We searched the IEDB using “Human” as Host, “Class II” as MHC Restriction, and “include the positive assays” and obtained around 500,000 epitopes with 2-dimensional (2D) sequences. After excluding the epitopes with 3-letter amino acids, numbers, modified residues such as CIR, and lengths smaller than 5-mer, we finally got 485,366 2D epitopes.

After obtaining the list of 485,366 epitopes in a “.txt” file, with the ID and sequence of each epitope stored on a single line and separated by commas, we used a script to convert the epitope information into individual “.ali” format files for each epitope sequence. The script reads a list of sequence IDs and sequences from the input file, generating corresponding “.ali” files in the output folder. Each “.ali” file is named after the sequence ID and includes a formatted header along with the associated sequence. To build the 3D structure of each epitope, we needed to identify the appropriate template(s) for each target. From our structure dataset, as shown in Table S3, we excluded the epitopes with nonessential amino acids, such as CIR, which are not considered templates. For epitopes that contain the same part of their sequences, we selected the longer one as a template. Thus, we have 55 diverse 3D structures of reported epitopes as templates set to conduct sequence alignments between each target epitope and the known structures. Essentially, the script automates the alignment of target epitope sequences (stored in “.ali” files) against a set of known 3D epitope structures using MODELLER [39,63]. During the sequence alignment, it classifies amino acids into specific categories and calculates sequence identity by comparing residue matches and similarities. The script reads the target sequences, loads the known structures as templates, and performs multiple sequence alignments to identify the best matching template for each epitope based on sequence identity. The results, including alignment files, the best template, and similarity score are saved, and all files are organized into a specified output directory. During this step, we created a dataset of 142,934 nonredundant epitope sequences, each sharing at least 25% sequence similarity, to build their 3D structures. Using the alignment files, we then employed an in-house script to build homology models for each target epitope. This script automates the homology modeling process using MODELLER (“automodel”), iterating through the target epitope sequences in “.ali” format, and identifying the corresponding template PDB structures from a predefined list. The script builds comparative models using the selected templates and target sequences, generating 3D structures of the epitopes. Each model is saved as a “.pdb” file, and all relevant alignment and model files are organized into specified output directories.

### Homology modeling techniques

We filtered sequences in the structure dataset to exclude 29 sequences with modification, like nonessential residues and CIR, and exclude the 1seb, whose sequence is composed of 13 alanines. Thus, 102 sequences of the cocrystallized epitopes that bind to MHC-II were selected from the structure dataset and employed to test the modeling performance of homology modeling techniques, including RF2, AF2, and MODELLER. These epitopes have no modifications such as CIR and acetyl group (ACE). AF2, developed by DeepMind, is a neural network-based model that can predict protein structures with atomic accuracy and even accuracy in circumstances where no similar protein structure is available [38]. We accessed AF2 via the Center for Research Computing (CRC) at the University of Pittsburgh and predicted structures for 102 epitope sequences using an A100 GPU (80GB SXM) with “db_preset” as “full_dbs” and “model_preset” as “monomer”. RF2 is another neural network-based protein structure prediction model that is more computationally efficient than AF2, and it has been reported to have parallel accuracy of AF2 on monomers [64]. We accessed RF2 on our server Hydra (Intel Core i9-13900K CPU, 24 cores, 128 GB) and utilized identical 102 sequences. MODELLER is used for homology or comparative modeling of protein 3D structures, which requires an alignment of a protein sequence to be modeled with the known related structures [39,63]. We performed homology modeling using MODELLER templates. Then, structural alignment was performed using PyMOL (TM) 2.5.7 between the modeled/predicted epitopes in 3 groups and their corresponding cocrystallized epitopes to calculate the RMSD values. The average RMSD values out of 102 pairs were identified as the criteria for comparing the modeling performances of 3 techniques. We employed analysis of variance (ANOVA) to evaluate our testing results, as shown in Fig. 3E.

### EpiMII architecture

The model architecture was encoder–decoder message passing neural networks (MPNNs) [26,65]. The overview is shown in Fig. 4A. The encoder layer had 3 hidden dimensions to update the node and edges. The decoder layer also had 3 hidden dimensions to iteratively decode the amino acid compositions to generate epitope sequences.

Graph representation (encoder processing). Peptides structures were depicted as graphs, where atoms served as nodes and interactions between atoms, such as bonds and distances, were depicted as edges. Each node (atom) was associated with a feature vector that encapsulated its atomic properties, including type, coordinates, charge, hydrophobicity, and any masked values. Similarly, each edge (interaction) was associated with a feature vector that encoded pairwise interactions between atoms, including bond type, distances (e.g., pairwise Euclidean distances), and potentially radial basis function (RBF) values. This initial representation converted the input structure into a graph format suitable for neural network processing.

Message passing layers (encoder processing). EpiMPNN-MHCII employed MPNN layers to propagate information between nodes (atoms) and update their representations based on interactions with neighboring nodes. This process involved iterative steps where each node aggregated information (messages) from its neighboring nodes and updated its representation accordingly.

$$h_{v}^{\left(l+1\right)}=\sigma \left(W\cdot h_{v}^{\left(l\right)}+\sum_{u\in N\left(v\right)}W_{\mathrm{euv}}\cdot h_{u}^{\left(l\right)}\right)$$ (1)

where $h_{v}^{\left(l\right)}$/$h_{u}^{\left(l\right)}$ is the hidden state of node v/u at layer l, σ is an activation function, W is a learnable weight matrix, and $W_{\mathrm{euv}}$ is a learnable edge-specific weight matrix. Typically, multiple message-passing layers were utilized to capture hierarchical and spatial features of the peptide structure.

Node and edge update functions (decoder processing). Within each message passing layer, node and edge update functions were applied to compute new node and edge representations based on aggregated information from neighboring nodes:

$$h_{v}^{\left(l+1\right)}=f_{\text{node}}\left(h_{v}^{\left(l\right)},\;\sum_{u\in N\left(v\right)}W_{\mathrm{euv}}\cdot h_{u}^{\left(l\right)}\right)$$ (2)

where $f_{\text{node}}$ is a learnable node update function.

Similarly, edge update functions process edge features and node embeddings to update edge representations, capturing spatial relationships and interactions within the peptide:

$$e_{\mathrm{uv}}^{\left(l+1\right)}=f_{\text{edge}}\left(e_{\mathrm{uv}}^{\left(l\right)},\;h_{v}^{\left(l\right)},\;h_{u}^{\left(l\right)}\right)$$ (3)

where $f_{\text{edge}}$ is the edge update function, $e_{\mathrm{uv}}^{\left(l\right)}$ represents the updated feature of the edge $\left(u,v\right)$ after layer l, $h_{v}^{\left(l\right)}$ is the feature of node v at layer l, and $h_{u}^{\left(l\right)}$ is the feature of node u at layer l.

Graph pooling (decoder processing). Following multiple message-passing layers, EpiMPNN-MHCII employed graph pooling operations to aggregate node representations and generate a global representation of the entire peptide structure.

$$z=\sum_{v\in V}W_{\text{pool}}*h_{v}^{\left(l\right)}$$ (4)

where z is the pooled representation, $W_{\text{pool}}$ is a learnable weight matrix, and l is the number of message-passing layers.

Training and optimization (decoder processing). EpiMII was trained using supervised learning techniques on large datasets of epitope structures with known properties, such as experimental structures from databases. During training, the model learned to minimize a loss function that measured the difference between predicted and actual epitope coordinates.

$$L=\sum_{i}{\left|x_{i}-x_{i}^{*}\right|}^{2}$$ (5)

where L is the loss function, $x_{i}$ is the predicted amino acid sequence*,* and $x_{i}^{*}$ is the actual peptide sequence.

Optimization methods, such as gradient descent, were employed to update the model’s parameters and improve its predictive performance over iterations:

$$\theta =\theta -\alpha \nabla_{\theta }L\left(\theta \right)$$ (6)

where θ is the set of learnable parameters, α is the learning rate, and $\nabla_{\theta }L\left(\theta \right)$ is the gradient of the loss function with respect to the parameters.

Sequence generation (decoder processing). Through an autoregressive approach, EpiMII meticulously predicted the amino acid sequence *x* given the underlying backbone structures, encapsulating the intricate relationship between structural elements and sequence composition:

$$p\left(x|s\right)=\prod_{i}p\left(x_{i}|s,\;x_{<i}\right)$$ (7)

Here, $p\left(x_{i}|s,\;x_{<i}\right)$ denoted the conditional probability of the amino acid $x_{i}$ at decoding step i, and $x_{<i}=\{x_{1},\ldots ,x_{i-1}$} referred to previously decoded residues. These probabilities were parameterized using 2 primary components: an encoder that computed node and edge embeddings from structural data, and a decoder that predicted the next decoded residue autoregressively based on preceding decoded letters and structural embeddings.

### EpiMII: Model training process

For the training dataset, we first tried to cluster the 485,366 MODELLER-modeled MHC-II epitopes by mmseqs2 with alignment mode 3 and the single-step clustering method based on the sequence identity: 30%, 25%, 20%, 15%, and 10% [66,67]. The dataset resulted in 274,353 clusters for sequence identity 30%, and 274,063 clusters for sequence identity 25%, 20%, 15%, and 10%. The models trained on these clusters all resulted in inadequate training and validation performance, in which validation perplexities were higher than 7, and accuracies were lower than 0.40. We then chose to clean the sequence dataset based on sequence similarity cutoffs of >30%, >28%, and >25%, while ensuring that each epitope in the dataset formed a single cluster. Sequence similarity was calculated as the percentage of similar residues in 2 sequences. The residues in 2 sequences that belong to the same biochemical groups, including nonpolar/hydrophobic, polar uncharged/hydrophilic, acidic/negatively charged, basic/positive charged, and others, were identified as similar residues. The dataset with larger than 30% sequence similarity had 20,022 epitopes. The dataset with larger than 28% sequence similarity had 44,019 epitopes. The dataset with a sequence similarity larger than 25% had 142,934 epitopes in total. We trained models on these 3 datasets and tested the sequence recovery using 102 modeled MHC-II epitopes, as shown in Fig. S4. Sequence recovery is a benchmark method of evaluating protein design tools, which passes the backbone of natural protein with the known amino-acid sequence as input. It measures the identity between the predicted and true sequences to evaluate the prediction accuracy [29]. We also trained the model with 485,366 clusters (each epitope in the sequence dataset was regarded as one individual cluster) without clustering on either sequence identity or sequence similarity. Although the training and validation performance of this model were parallel to the model trained on the dataset with sequence similarity >25%, the tested sequence recovery using 102 modeled epitopes (mean average: 0.6623, median average: 0.7308) was worse than the dataset with sequence similarity larger than 25% (mean average: 0.7865, median average: 0.8375). Thus, we determined to use 142,934 MODELLER-modeled nonredundant epitopes (sequence similarity > 25%) with their 3D structures and 2D sequences as our dataset in this study.

The dataset was then using mmseqs2 to cluster the data using the cutoff as sequence identity = 25%, then split into train, validation, and test sets in 8:1:1 to ensure that the epitopes in the test set do not have similar ones appearing in the training or validation set. Each modeled epitope was set up as one cluster. We trained the model with the following setup: “hidden_dim” as 128, the number of neighbors for the sparse graph as 48, epoch as 200, “batch_size” with 2,048 tokens, reload data every 4 epochs, save model every 2 epochs, and the “gradient norm” is −1. The dropout rate is 0.1, and the backbone noise is 0 Å. Models were trained using automatic mixed precision. The input features of the model were embedded edges without node features, which were the Euclidean space and distances between the residues in the primary sequence space with the same chain ID indicator, “_A”. The distances between 2 residues are encoded using the Gaussian RBFs equally spaced from 0 to 20 Å between atoms, N, Cα, C, O, and Cβ. The virtual Cβ coordinates were calculated as: $\begin{array}{c} \mathrm{C\beta}=-0.58273431*a+0.56802827* \end{array}$$\begin{array}{c} b-0.54067466*c+\mathrm{C\alpha};b=\mathrm{C\alpha}-N,c=C-\mathrm{C\alpha},a=\text{cross}\left(b,\;c\right) \end{array}$, which represents the ideal angle and bond length definitions. The training loss was defined by: $\begin{array}{c} \text{Loss average}=\mathrm{sum}\;\left(\text{loss}*\text{mask}\right)/2,000 \end{array}$; 2,000 was chosen empirically, loss (categorical cross entropy per token) and mask had shapes [batch, protein length]. The optimization was using Adam with beta1 = 0.9, beta2 = 0.98, epsilon = 10^−9^. The models were trained on CRC of the University of Pittsburgh, using pytorch 2.0.1, cuda 11.8 with an A100 GPU, and a memory of 40 GB. The training and validation losses (perplexities) converged around 150k to 200k optimization steps, about 200 epochs of the training data.

### EpiMPNN-MHCII: Model utilization

We used the early stopped model weights on epoch 50, step 47,050, as the final model to design the sequences of neoantigen samples. We modeled the sequences of neoantigens to the 3D structures via MODELLER and input into our model. The backbone noise was set to zero, and the number of sequences to be generated was set to 16. The “designed_chain” was the single chain of the neoantigen, and no chain was fixed (fixed_chains = []). The outputs were the original neoantigen sequence and 16 sequences of the designed neoantigen. Designed peptides and their sequence recoveries (seq_recovery) were recorded. As for the masking strategy, we first aligned the modeled neoantigen structure with the standard cocrystallized epitope extracted from “1bx2.pdb” to number the residue positions of the neoantigen sequence. Then, the positions to be masked on the neoantigen sequence can be set up through “fixed_positions_dict”. Our mask strategies are shown in Fig. 4C. We used “mask no residues”, “ mask conserved positions”, “mask non conserved positions”, and “mask the positions of important interacting residues” to generate a list of sequences with different sequence recoveries. Because the masked residues did not participate in the calculation of sequence recovery, we finally selected the sequences with no more than 5 residues changed compared to the original neoantigen sequence for further validation.

### Per-position log-probability (log*P* vs. log*P*′)

We computed residue-level log-probabilities from the EpiMII (ProteinMPNN-like) decoder in 2 modes that differ only in how inputs are featurized and whether masking/sampling is applied. Importantly, the design region is the full epitope chain in both cases; what changes is whether the decoder is asked to reconstruct masked residues (log*P*′) versus score the native sequence without perturbation (log*P*).

Per-position log*P* (evaluation mode; no masking/sampling) was calculated by: Inputs are prepared with featurize function, which builds tensors (X, S, mask, chain_M, residue_idx, and chain_encoding_all) for the peptide chain(s). In our scoring call, the decoder is run in model.eval() with no random noise and no application of positional design masks. Practically, this means the native sequence across the entire epitope is visible to the model, and the output log-probability at each position is the decoder’s confidence for the observed residue under full structural context. We keep only valid positions (mask==1). This metric is used when we want the model’s raw structural likelihood of the exact input structure/sequence pair (e.g., crystal vs. MODELLER comparisons).

Per-position log*P*′ (test mode; with masking/sampling) was calculated by: Inputs are prepared with tied_featurize function, which in addition to the basic tensors also returns chain_M_pos, omission masks, and optional PSSM/bias tensors. We supply a chain_id_dict so that the peptide chain is treated as designable (masked); the forward pass multiplies the design mask (chain_M * chain_M_pos) and injects a Gaussian tensor randn to mimic test-time stochasticity. The decoder thus reconstructs the peptide residues position-by-position from partial/perturbed context, and the per-position log*P*′ we report is the log-probability assigned to the true amino acid at each site under this masked, sampled setting. This test-style metric is used for downstream interpretability and consistency analyses, including core enrichment, anchor enrichment, and sliding-window overlap, because it better reflects predictive behavior on unseen sequences. The per-position log*P* quantifies how well the given input residue fits its structural environment, while log*P*′ measures the model’s confidence of placing any of the 20 amino acids at a given structural position. Thus, log*P* reflects sequence-to-structure compatibility, whereas log*P*′ captures structure-to-sequence designability.

### Core enrichment and sliding window overlap analysis

For each peptide–MHC II complex, a 10-mer structural binding core (“RMSD core”) was defined by aligning the query structure to a crystallized reference template using Cα-based Kabsch superposition. The crystallized reference template for each query was selected from 55 MODELLER-generated templates based on sequence similarity. Template cores were preannotated from the structure alignment of the crystallized peptide–MHC II complexes, and the core positions were numbered as shown in Fig. 1F. For each query structure, all contiguous 10-mer segments were aligned to the reference core, and the segment with the minimal RMSD was designated as the structural RMSD core.

After calculating the per-position log*P*′ for the inputs, we used a 10-mer sliding window on the per-position log*P*′ profile. The 10-mer segment with the maximal summed log*P*′ was identified as the log*P*′ core. Core enrichment was calculated as the difference in mean per-position log*P*′ between residues inside and outside the log*P*′ core. Sliding-window overlap quantified the consistency between structural and probabilistic core definitions by comparing the RMSD core with the log*P*′ core, calculated as the fraction of shared residues between the 2 10-mer segments. These analyses comprehensively evaluated whether EpiMII assigns high confidence to structurally defined binding regions and canonical anchor positions.

### AlphaFold2-multimer and AlphaFold3

AF2 is a neural network-based model that can predict protein structures with atomic accuracy [38]. AF2 was accessed via the CRC at the University of Pittsburgh and ran on an A100 GPU (80GB SXM). Because AF2 is more accurate in predicting structure when we input both the sequences of epitope and MHC-II compared to the epitope sequence only, we input the sequences of the designed neoantigen and the whole MHC-II that the original neoantigen binds to make the structural prediction. The complete sequences of the alpha and beta chain of the specific type of MHC-II were collected from the HLA Nomenclature (https://hla.alleles.org/pages/nomenclature/naming_alleles/). We then chose the epitope of 1bx2.pdb as the reference structure for the neoantigens that bind to HLA-DRB1 encoded MHC-II. We employed AF2 to predict the structures of designed neoantigens with “db_preset” as “full_dbs” and “model_preset” as “multimer”. We also use AF3 to perform complex structure prediction, using the sequences of designed epitope and G-domain. AF3 was accessed on our own server Laker (Intel Core i9-13900K CPU, 24 cores, 128 GB, A6000 GPU). We performed the structural alignment for the predicted structure of the designed epitope and the reference structure using PyMOL. RMSD was also calculated. Whether the shape of the designed neoantigen is similar to the cocrystallized reference structure as well as the RMSD were recorded.

### C-ImmSim

C-ImmSim is the C-language-based immune system simulator [48]. With a given agent, this online tool can predict the immune response it may trigger in both MHC-I and MHC-II antigen presentation pathways. C-ImmSim also predicts the MHC binding. We input the designed neoantigens into it to predict the potential immune response they can trigger. C-ImmSim is available at https://kraken.iac.rm.cnr.it/C-IMMSIM/index.php. We set the basic parameters as the default value: random seed as 12,345, simulation volume as 10, simulation steps as 100, and time step of injection as 1. The injecting agent is a vaccine with no lipopolysaccharide (LPS). The number of agents to inject is 1,000. We input the designed neoantigen sequences with the FASTA format and input one sequence at a time. We defined the “Y” symbols as a label for epitopes that can trigger CD4^+^ T cell immune response. The criteria for assigning “Y” are as follows: (a) the MHC-II presentation curve is active, and (b) the maximum IFN-γ value exceeds 6,000 predicted units.

### Synthesize linear peptides from the C-terminus to the N-terminus direction

The solid-phase peptide synthesis process involves sequential coupling of amino acids from the C-terminal to the N-terminal, adhering to a systematic protocol. Initially, RINK resin is soaked in dichloromethane and washed with *N*,*N*-dimethylformamide (DMF) to prepare it for coupling reactions. Fmoc protecting groups on the resin are removed using piperidine/DMF, confirmed by ninhydrin test. Coupling of each amino acid with 1-hydroxybenzotriazole (HOBT) and N,N′-diisopropylcarbodiimide (DIC) is conducted at 30 °C, followed by acetylation and washing steps. This cycle is repeated for each subsequent amino acid until the sequence is complete. The peptide is then cleaved from the resin using a cleavage solution and purified via HPLC under specified conditions. The process ensures high purity (≥95% purity) and accuracy in peptide synthesis.

### In vitro validation of peptides

Human PBMCs were obtained from a 32-year-old healthy Chinese man with the DRB10101 genotype at Shenzhen Second People’s Hospital. Separate PBMCs and coincubate with peptides (2 μM) and add 15% fetal bovine serum and IL-2 (1.7628 μg/ml) to RPMI 1640. After 11 days, the cells were placed in RPMI 1640 supplemented with 8% human serum and without any IL-2. On the 12th day, wash the cells with RPMI 1640, dilute with 2 × 10^6^/ml, and inoculate 2 × 10^5^ cells into each well of a 24-well plate. Then, add 100 μl of peptide (2 mM) to each plate and incubate the cells at 37 °C for 1 h. Add protein transport inhibitors and incubate the cells at 37 °C for an additional 4 h. Surface labeling staining was performed using PerCP/Cyanine5.5 Anti Human CD3 Antibody, FITC Anti Human CD4 Antibody, and CD4, while intracellular factor labeling staining was performed using PE Anti Human IFN-γ Antibody, APC Anti Human IL-4 Antibody, and HU TNF PE-CY7 MAB. Flow cytometry was used for final testing.

### Mouse models

All animal experiments were approved by the Animal Ethics Committee of Zhejiang University of Technology (Approval No. IACUC-MGS20241030021). Female C57BL/6J mice (4 to 6 weeks old) were purchased from Hangzhou Hans Biological Technology Co., Ltd., and housed in pathogen-free facilities at the Experimental Animal Center. The mice were fed a standard diet and provided with distilled water ad libitum. Sterilized cages with fresh bedding were supplied weekly. At the end of the experiment, the mice were anesthetized with sodium pentobarbital (60 mg/kg) and euthanized using sodium pentobarbital (100 mg/kg). Measures were taken to minimize animal suffering. C57BL/6J mice carrying 5 × 10^6^ subcutaneous (s.c.) Hepa 1 to 6 tumor cells were randomly assigned to treatment groups (6 mice per group), with an average tumor volume of 100 to 150 mm^3^ per group. Starting from day 0, MHC-II peptides were subcutaneously injected at a dose of 20 μg twice weekly. Every other day, use a digital caliper to measure the size of the tumor and observe the weight and survival rate, and tumor volume was calculated as length × width^2^ × 0.5. One mouse in the P2 and P3 groups died naturally during the experiment. In compliance with animal ethics rules, the data for mice with tumors that grew too fast were processed as humane endpoints before the end of the experiment.

### ELISA was used to determine the concentrations of IFN-γ and TNF-α in serum

The murine IFN-γ ELISA employs a double-antibody sandwich method to quantify IFN-γ levels in serum, plasma, or related fluids. Microplate wells are precoated with an anti-IFN-γ antibody, followed by the addition of standards or samples and a horseradish peroxidase (HRP)-labeled detection antibody to form an immune complex. After washing, 3,3′,5,5′-tetramethylbenzidine (TMB) substrate is added, producing a color change proportional to the IFN-γ concentration, which is measured at 450 nm. The concentration is determined using a standard curve, and appropriate dilutions are applied to calculate the actual sample values. The determination of TNF-α is the same.

### HE staining

Paraffin sections of tumor were deparaffinized by sequential immersion in Xylene I (8 min), Xylene II (8 min), Xylene III (8 min), Absolute Ethanol I (5 min), Absolute Ethanol II (5 min), 85% Ethanol (5 min), and 75% Ethanol (5 min), followed by rinsing in tap water for 2 min. Hematoxylin staining was performed for 6 min, followed by differentiation in hydrochloric acid ethanol for 2 s and rinsing with water. The sections were blued in ammonia solution for 15 to 30 s and washed again with water. Eosin staining was conducted by dehydrating the sections in 95% ethanol for 1 min, followed by staining in eosin solution for 10 to 30 s. For dehydration and mounting, the sections were sequentially immersed in Absolute Ethanol I (30 s), Absolute Ethanol II (2.5 min), Absolute Ethanol III (2.5 min), Xylene I (2.5 min), and Xylene II (2.5 min) for clearing, then mounted with neutral resin. Finally, the sections were examined and analyzed under a microscope with image acquisition.

### TUNEL staining

The procedure of TUNEL staining began with deparaffinization of tissue sections by sequential immersion in Xylene I (12 min), Xylene II (12 min), Absolute Ethanol I (6 min), 95% Ethanol (6 min), and 85% Ethanol (6 min), followed by rinsing in distilled water for 2 min. Antigen retrieval was performed using a microwave at medium power for 8 min with citrate antigen retrieval buffer (pH 6.0), followed by cooling at room temperature and triple rinsing with double-distilled water (5 min each). After air-drying, a hydrophobic barrier was drawn around the tissue using a histochemical pen, leaving a 3- to 4-mm margin, and washed 3 times with pure water (5 min each). The TUNEL working solution was prepared by mixing TdT enzyme (1 μl) with reaction buffer (50 μl), applied to the circled tissue, and incubated in a humidified chamber at 37 °C for 1.5 h (adjustable based on experimental results). The sections were washed in phosphate-buffered saline (PBS) (pH 7.4) 3 times (5 min each) on a shaker, excess PBS was removed, and 4′,6-diamidino-2-phenylindole (DAPI) staining solution was added for nuclear staining under light protection at room temperature for 8 min. After washing in PBS 3 more times, the sections were mounted with an antifluorescence quenching mounting medium. Finally, the slides were examined and imaged under a fluorescence microscope, using appropriate excitation and emission wavelengths for DAPI (UV excitation: 361 to 389 nm, emission: 420 nm, blue fluorescence) and FITC (excitation: 465 to 495 nm, emission: 515 to 555 nm, green fluorescence).

### TCR sequencing

Terminate the culture and collect the cells. The cells were washed with PBS to remove residual peptides and blockers, then resuspended to prepare a cell suspension at a concentration of 700 to 1,200 cells/μl. The single-cell suspension was loaded into the microfluidic chip of the Chip A Single Cell Kit v2.1 (MobiDrop, S050100301). Using the MobiNova-100 system (MobiDrop, A1A40001), the single-cell suspension, reverse transcription reagents, and MobiNova beads were combined to generate droplets. Each cell was isolated within an individual water-in-oil droplet. Subsequently, light cut was performed using the MobiNovaSP-100 (MobiDrop, A2A40001). Cell lysis was carried out within the droplets, and the released mRNA was captured by the sequences on the beads. The cell barcodes were linked to the cDNA products through reverse transcription. The droplets were then broken, and PCR amplification was performed using the cDNA as a template. Libraries were constructed using the High Throughput Single-Cell 3′ Transcriptome Kit v2.1 (MobiDrop, S050200301) and the 3′ Dual Index Kit (MobiDrop, S050300301). The constructed library was sequenced, and the resulting raw sequencing data were analyzed using MobiDrop’s analysis software for cell typing and differential analysis. Subsequently, functional enrichment analysis was performed on these differentially expressed genes to identify the functional characteristics of the subpopulations.

### Synthesis of TCR

The TCR sequence was cloned into an expression vector and transformed into competent cloning bacteria. Endotoxin-free, transfection-grade plasmid DNA was extracted. CHO-Express cells were transiently transfected with the plasmid and cultured in suspension at 37 °C with 5% CO₂ in serum-free expression medium. On day 7 posttransfection, the cell culture supernatant was collected by centrifugation and purified using an AmMag Ni Magnetic Beads affinity chromatography column. After washing and elution, TCR was obtained.

### Assembly of HLA–peptide complexes

The peptides were dissolved in appropriate solvents: MUT peptide (sequence: AYHASKYEFLANLHIT) and P4 peptide (sequence: AYHASKYEALANLWTH) in ddH₂O, and WT peptide (sequence: AYHASKCEFLANLHIT) in 5% formic acid + 15% acetonitrile + 80% H₂O, to a final concentration of approximately 532 μM. Then, 13 μl of each peptide solution was mixed with 0.2 ml of HLA protein (sinobiological, at a concentration of 1.3 mg/ml) and incubated at 4 °C in the dark for 36 h to obtain the HLA–peptide complexes.

### SPR affinity test of HLA–peptide complex and TCR

The TCR protein (ligand) was diluted to 30 μg/ml in acetate buffer (pH 4.5) and immobilized onto a CM5 sensor chip (Cytiva, BR-1005-30) at a flow rate of 30 μl/min. Flow cell 1 (Fc1) was used as the reference channel, and Flow cell 2 (Fc2) was used as the immobilization channel. The pMHCII complexes were diluted in 0.05% PBST to concentrations of 5, 2.5, 1.25, 0.625, and 0.3125 μM, and injected sequentially over the chip surface. The contact time was 90 s, and the dissociation time was 120 s. Sensorgram data were analyzed using Biacore 8K+ evaluation software, and the equilibrium dissociation constant (*K*_D_) was calculated by fitting the data to a 1:1 binding model.

### Statistics

We employed one-way ANOVA data analysis to compare the modeling performance among AF2, RF2, and MODELLER. The *F* value was 147.5248, and the factor degree of freedom was 2. The *P* value was 1.39e−45. The in vitro experiments of polypeptides were repeated 3 times for verification. All used ordinary one-way ANOVA with degrees of freedom of 6. The *F* value of IFN-γ analysis was 44.89, the *F* value of TNF-α analysis was 119.6, the *F* value of IL-4 analysis was 42.23, and the *F* value of Th1/Th2 ratio analysis was 33.12. In the in vivo validation, each group’s tumor volume histograms used SEM mean statistics. The pooled line graphs were analyzed using two-way ANOVA data analysis with multiple comparisons between individuals. The row degree of freedom was 6, the *F* value was 19.63, the column degree of freedom was 11, and the *F* value was 6.26. ELISA measured the content of IFN-γ and TNF-α in serum twice with duplicate wells. Both used ordinary one-way ANOVA. The degree of freedom of IFN-γ content was 6, and the *F* value was 9.833. The degree of freedom of TNF-α content was 6, and the *F* value was 0.8782. The apoptotic index measured by TUNEL 3 times was also analyzed by one-way ANOVA with a degree of freedom of 6, and the *F* value was 3.789. Each group’s apoptotic index histograms used SD mean statistics.

## Ethical Approval

All animal experiments were approved by the Animal Ethics Committee of Zhejiang University of Technology (Approval No. IACUC-MGS20241030021). Experimental animals were provided under quality certificate No. 20250219Abzz01009990139. All animals were housed in the Experimental Animal Center of Zhejiang University of Technology under specific pathogen-free (SPF) conditions, with a controlled temperature of 22 ± 1 °C, a relative humidity of 50%, and a 12-h light/dark cycle. Mice were maintained in polypropylene cages at a density of 6 animals per cage, fed standard pellet diets in accordance with GB13078 and GB14924.3-2010 standards, and provided sterilized water ad libitum. All experimental procedures were conducted in accordance with the relevant ethical guidelines and regulations.

## Data Availability

The dataset (PyTorch .pt format), the structure dataset containing 142,934 modeled MHC-II epitopes (Protein Data Bank .pdb format), and all model weights are available via Zenodo (https://doi.org/10.5281/zenodo.14767257). The details of 133 cocrystallized MHC-II epitopes collected from PDB can be found in Table S3. The structures of 133 cocrystallized epitopes, the original sequence dataset that contains around 480,000 MHC-II epitope sequences, and the T cell-positive/negative epitopes dataset collected from IEDB can be found in Github (https://github.com/JIY106/EpiMII.git). The raw data used in the experimental validations can be found in Zenodo (https://doi.org/10.5281/zenodo.14767107). All other data sources can be found in supplementary figures and tables. The source code of EpiMII is freely available on GitHub under Apache-2.0 license and can be downloaded on GitHub (https://github.com/JIY106/EpiMII.git). The code for running MCCS scoring techniques can be found in supplementary figures and tables.
