# Supplementary material for: EpiMII: Structure-Aware Graph Neural Networks for MHC-II Epitope Generation
Source: Research (Wash D C). 2026 Jun 15;9:1311. doi: 10.34133/research.1311 (PMC13266056; doi:10.34133/research.1311)
Supplement: Supplementary 1 — Figs. S1 to S13 Tables S1 to S11 [file research.1311.f1.docx]

**EpiMII: Structure-Aware Graph Neural Networks for MHC-II Epitope Generation**

Jiayi Yuan^1,#^, Xiaowei Xu^2,#^, Ze-Yu Sun^1^, Shan Zhu^3^, Chunyu Wei^3^, Tianjian Liang^1^, Jingxuan Ge^4^, Xiang-Qun Xie^1^, Yan Chen^2,*^, Zhiwei Feng^1,3,*^, Tingjun Hou^4,*^, Ying Xue^1,3,5,*^

^1^Department of Pharmaceutical Sciences, Computational Chemical Genomics Screening Center, and Pharmacometrics & System Pharmacology PharmacoAnalytics, School of Pharmacy; National Center of Excellence for Computational Drug Abuse Research, University of Pittsburgh, Pittsburgh, Pennsylvania 15261, United States

^2^College of Pharmacology Sciences, Zhejiang University of Technology, Huzhou 313099, Zhejiang, China

^3^Faculty of Pharmaceutical Sciences, Shenzhen University of Advanced Technology, Shenzhen 518107, Guandong, China

^4^College of Pharmaceutical Sciences, Zhejiang University, Hangzhou 310058, Zhejiang, China

^5^Department of Pharmacy, Zhongshan Hospital, Fudan University, Shanghai 200032, China

^*^To whom correspondence should be addressed.

**Supplementary Figures**


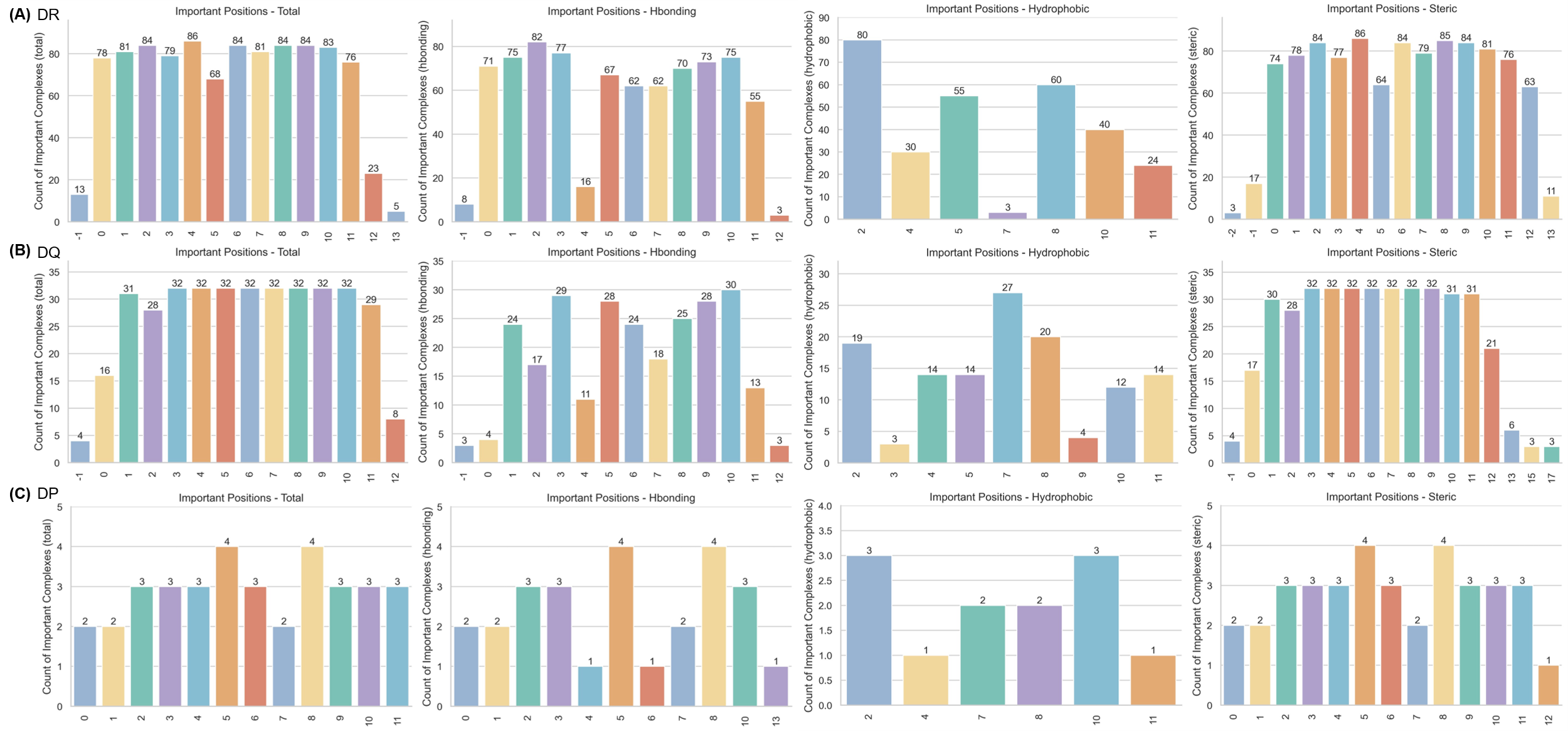


**Figure S1**. The MCCS scoring results of the 133 co-crystalized epitopes in (A) DR-epitope complexes (89), (B) DQ-epitope complexes (32), and (C) DP-epitope complexes (6), respectively, based on the previously defined position numbers in Figure 1(E). The results indicate how many residues in each position of epitopes highly contribute to the total binding to MHC-II (< -0.7 kcal/mol), to form hydrogen bonds (hbond) (< -0.3 kcal/mol), to form hydrophobic interactions (< -0.3 kcal/mol), and to form steric interactions (< -0.3 kcal/mol).


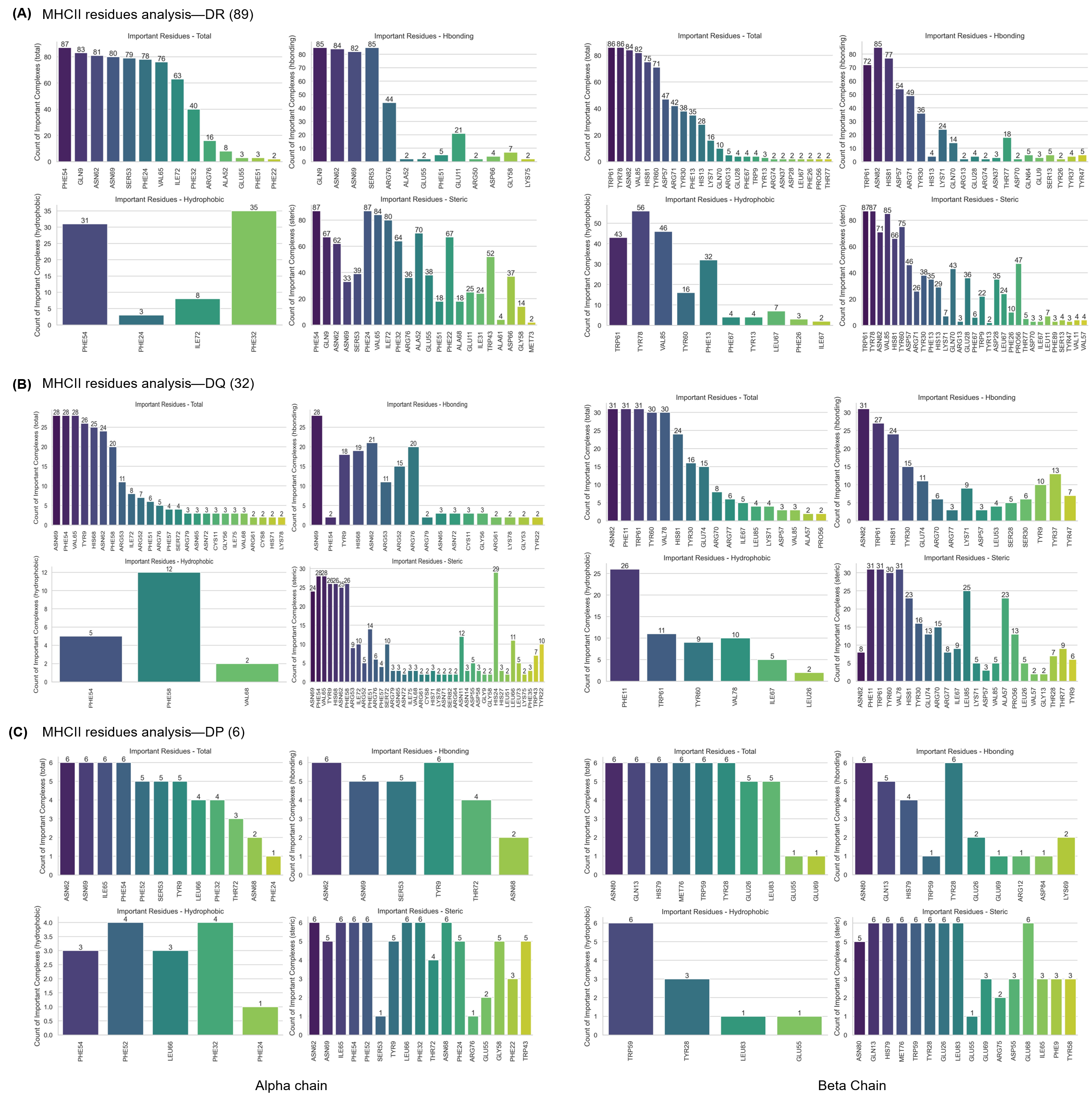


**Figure S2**. The MCCS scoring results of the 133 co-crystalized MHC-II G-domains in (A) DR-epitope complexes, (B) DQ-epitope complexes, and (C) DP-epitope complexes, respectively. The x-axis shows the residue's names. The y-axis is the count of complexes. The results indicate how many G-domain of the complexes have this residue that highly contributes to the total binding to its binding epitope (< -0.7 kcal/mol), to form hydrogen bonds (hbond) (< -0.3 kcal/mol), to form hydrophobic interactions (< -0.3 kcal/mol), and to form steric interactions (< -0.3 kcal/mol).


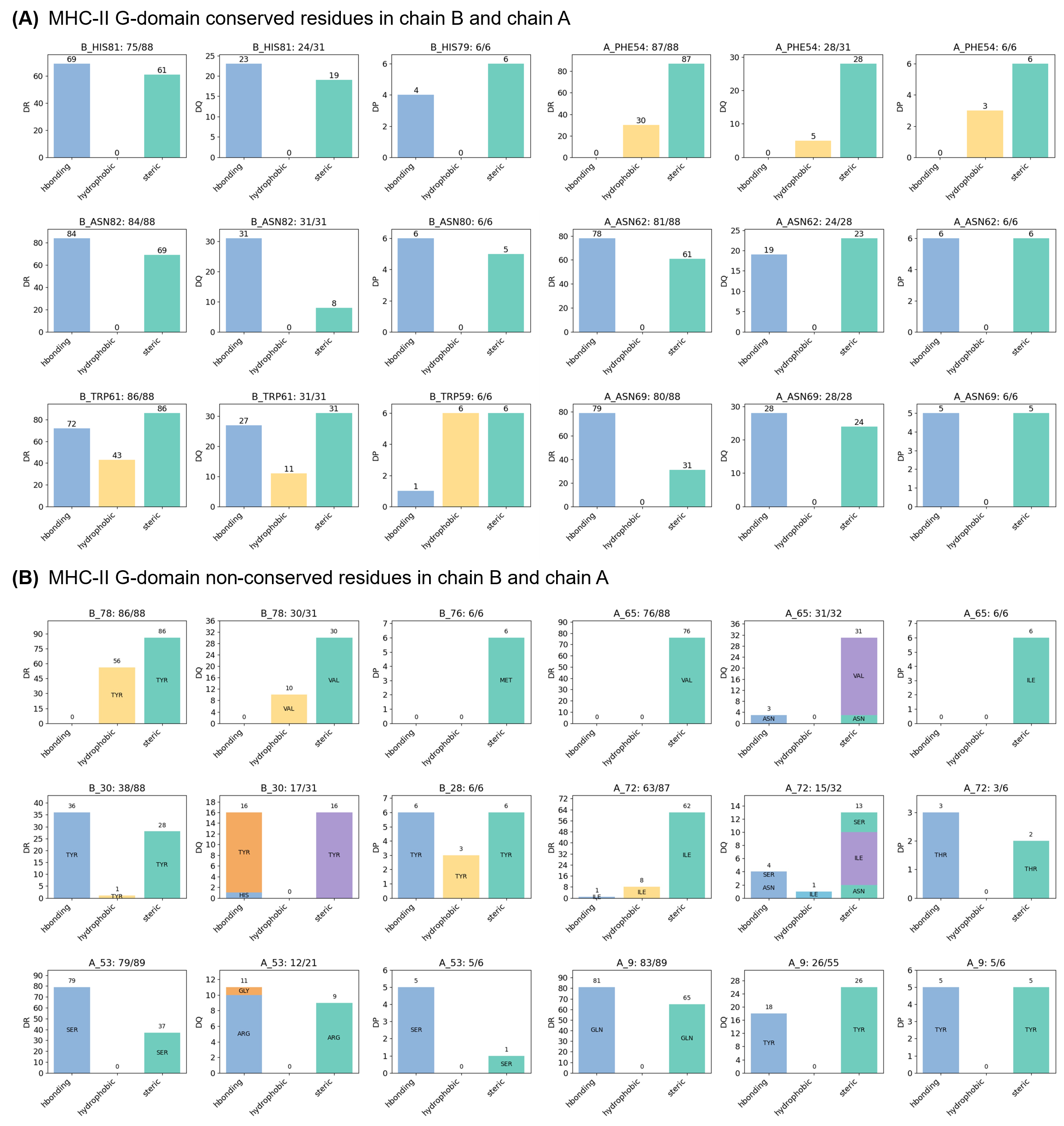


**Figure S3**. The detailed information on the interactions of the (A) conserved and (B) non-conserved residues on the G-domain. For example, ‘B_HIS81:75/88, B_HIS81: 24/31, B_HIS79: 6/6’ means the residue HIS81/79 is a conserved residue at the same positions in the DR, DQ, and DP-encoded MHC-II G-beta domains. For DR/DQ encoded MHC-II, HIS is numbered as 81, but for DP-encoded MHC-II, HIS is numbered as 79. ‘75/88’ means 75 DR-encoded MHC-II have this residue with total energy < -0.7 kcal/mol compared to 88 DR-encoded MHC-II with this residue in their sequences without missing (The total number of DR-encoded MHC-II is 89). The x-axis shows the types of interactions. The y-axis indicates the number of MHC-II that have this residue to form different types of interactions.


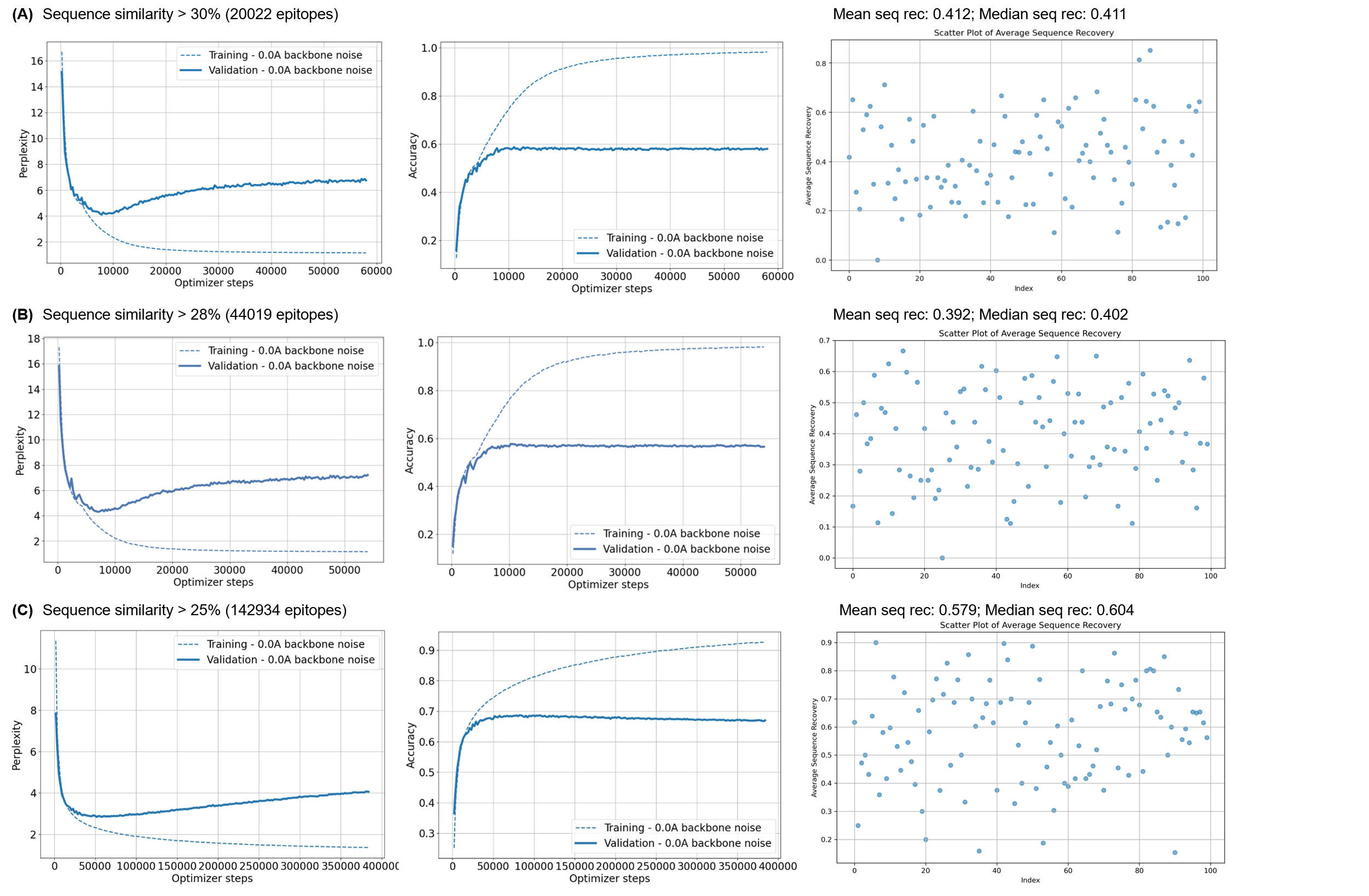


**Figure S4**. The simple ProteinMPNN model pre-training results. (A) The dataset which contains 20022 epitopes with sequence similarity larger than 30%. Training/Validation perplexity of the final epoch: 1.156/6.756; Training/Validation accuracy of the final epoch: 0.982/0.580. Mean sequence recovery: 0.412; Median sequence recovery: 0.411. (B) The dataset which contains 44019 epitopes with sequence similarity larger than 28%. Training/Validation perplexity of the final epoch: 1.156/7.218. Training/Validation accuracy of the final epoch: 0.982/0.565. Mean sequence recovery: 0.382; Median sequence recovery: 0.402. (C) The dataset which contains 142934 epitopes with sequence similarity larger than 25%. Training/Validation perplexity of the final epoch: 1.364/4.063; Training/Validation accuracy of the final epoch: 0.927/0/670. Mean sequence recovery: 0.579; Median sequence recovery: 0.604.


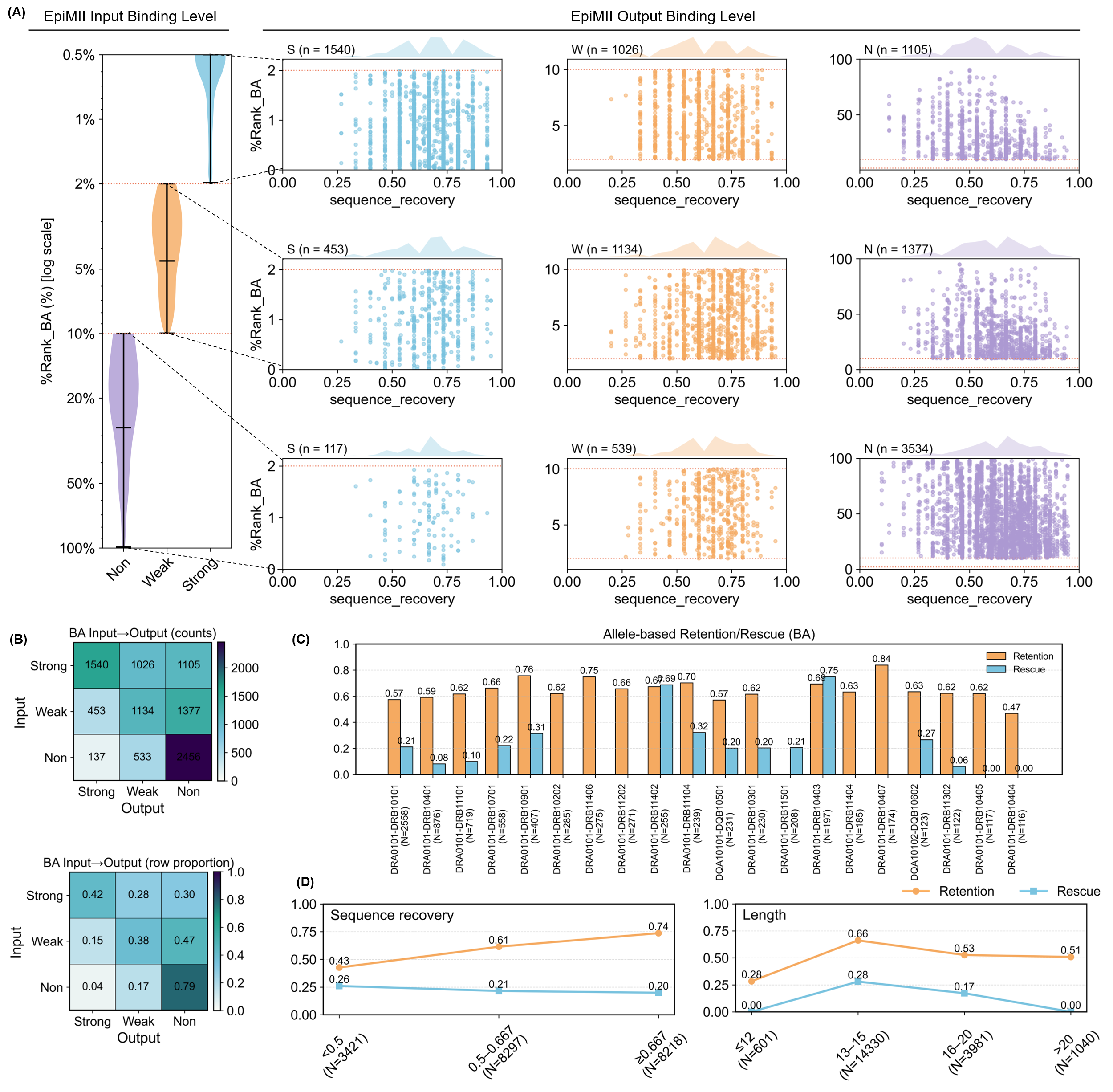


**Figure S5**. The evaluation of whether EpiMII preserves MHC-II binding using %Rank_BA as the primary endpoint. (A) The overview of Input→Output flows (counts). The left panel is the input bin, which contains 1771 T cell positive epitopes that are divided into non-binder (%Rank_BA < 2), weak binder (2 ≤ %Rank_BA < 10), and strong binder (%Rank_BA ≤ 10) as predicted by NetMHCIIpan-4.3. The right panel shows the outputs from EpiMII with sequence recovery per sequence and NetMHCIIpan4.3-predicted %Rank_BA score. The NetMHCIIpan-4.3 predicted positive-binding epitopes in (A) and predicted negative-binding epitopes in the T cell negative set are used for analysis in B, C, and D. (B) The heatmap of the counts and row-normalized proportions. (C) Allele-level Retention and Rescue (top 20 MHC-II types by sample size, sample size N≥30). The y-axis shows the proportion. Group means are at the top of each bar. (D) Left: Retention/Rescue versus sequence recovery of the designed output (bins: <0.5, 0.5–0.667, ≥0.667). The y-axis shows the proportion. Right: Retention/Rescue versus peptide length (≤12, 13–15, 16–20, >20 aa).


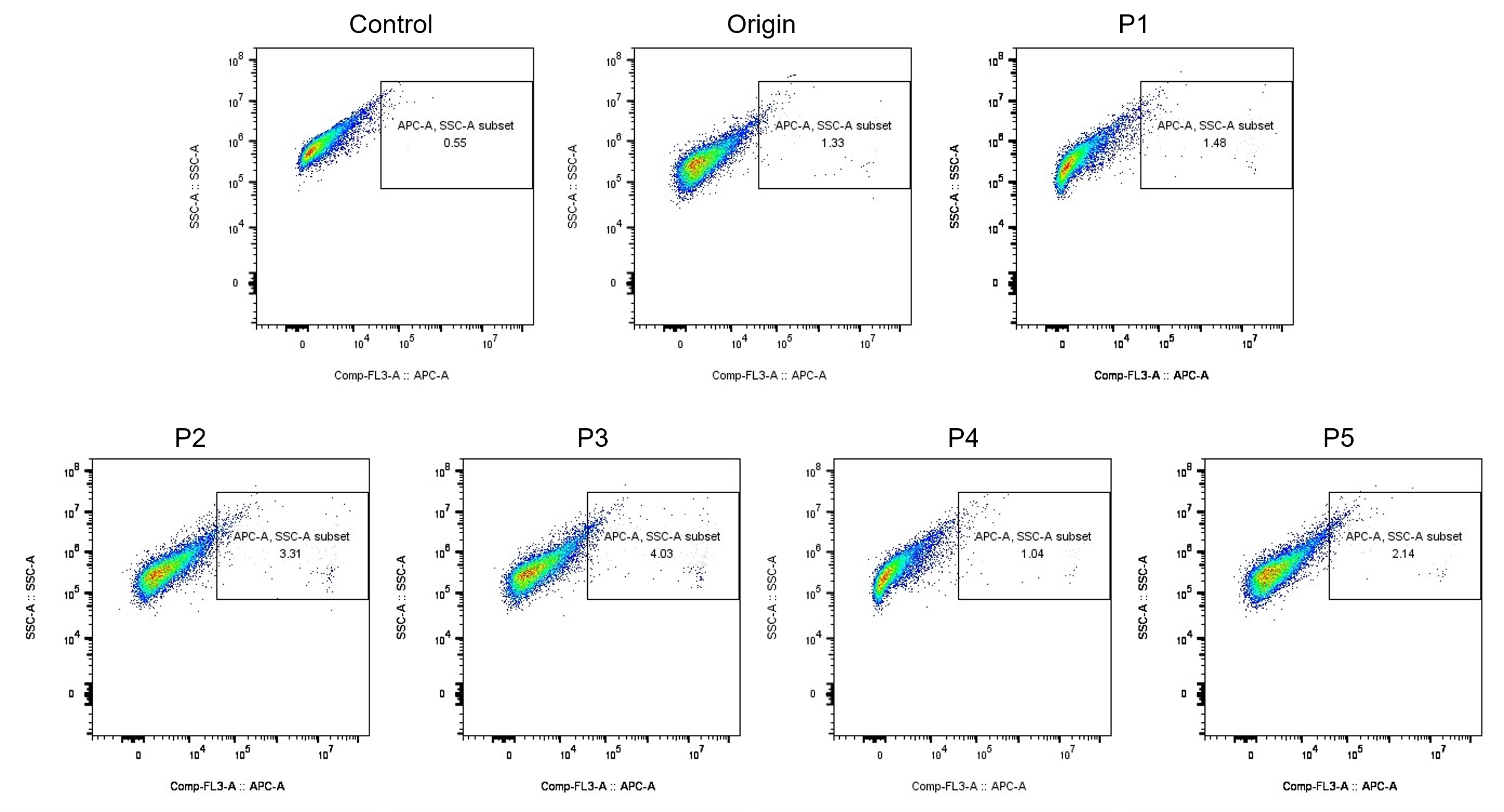


**Figure S6**. Secretion of IL-4 by CD4^+^ T cells in each group (IL-4 detected by APC).


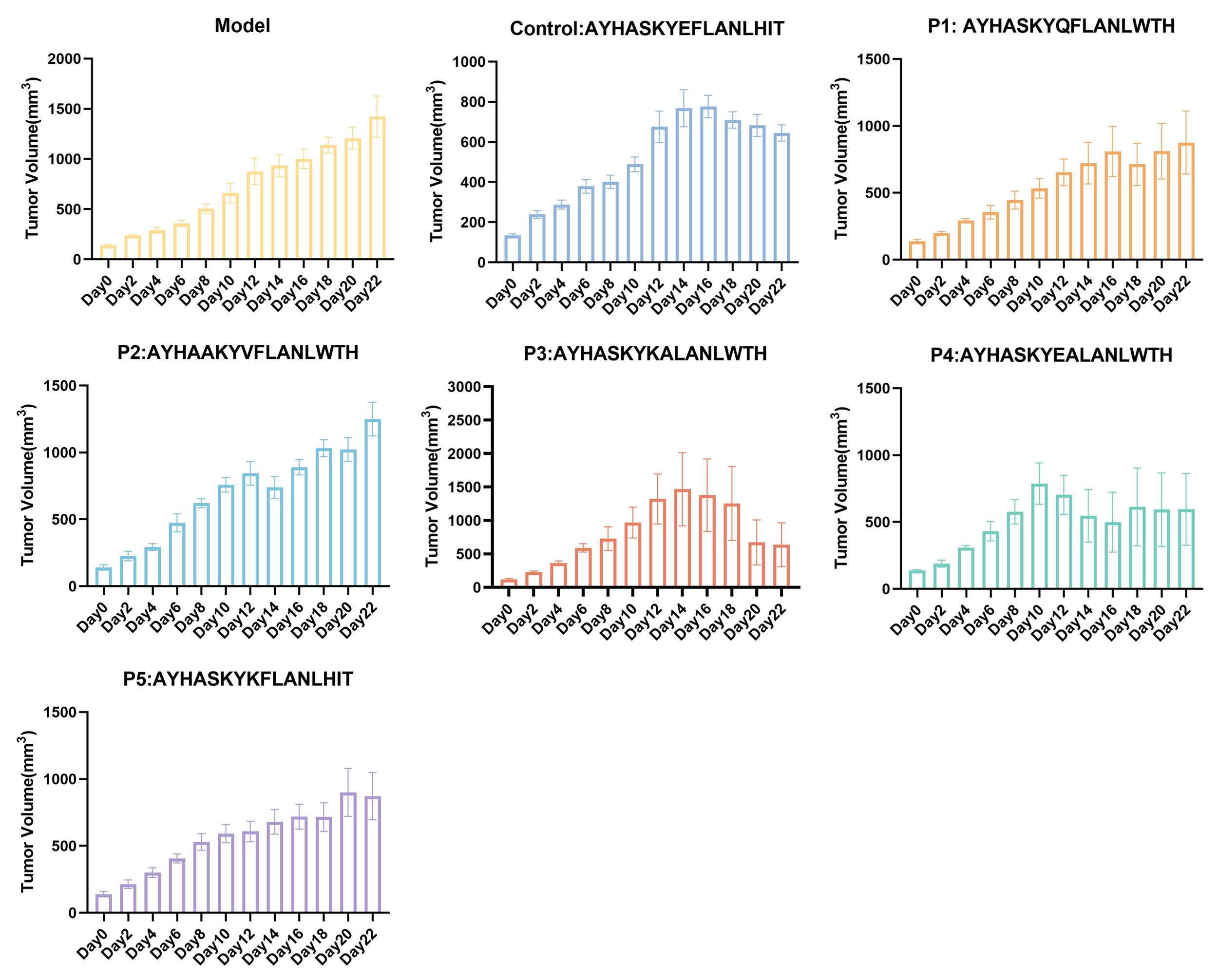


**Figure S7**. Tumor growth of mice in each group in 0-22 days. The error bar is shown as SEM.


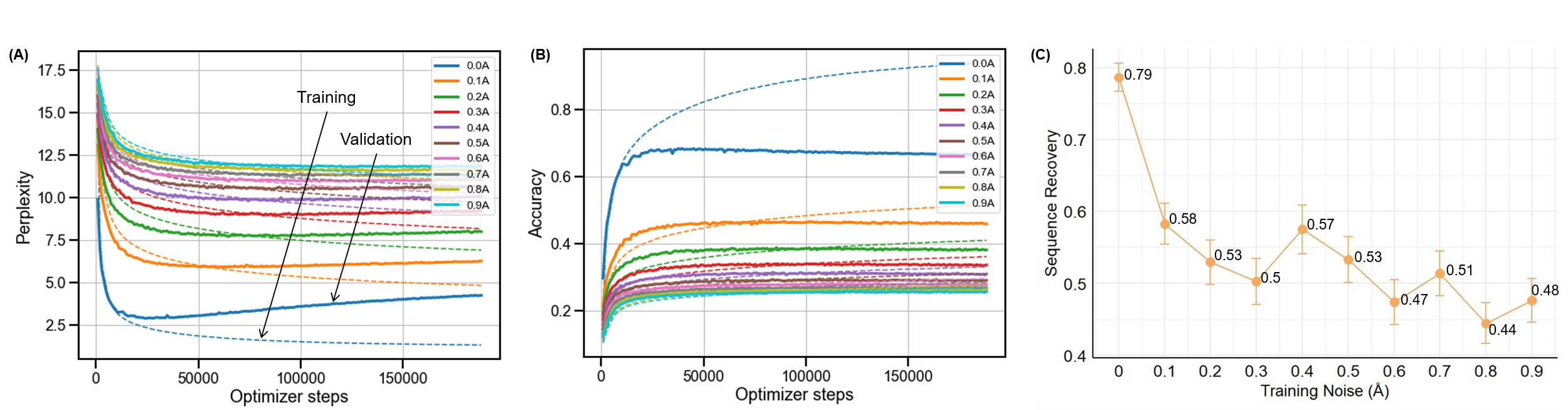


**Figure S8**. The training and validation performance of EpiMII with different backbone noises, ranging from 0 to 0.9 Å, is shown in (A) perplexity and (B) accuracy. Training results are shown as dash lines, while validation results are shown as solid lines. (C) The output sequence recovery of models with different training backbone noises using the last epoch as model weight and 103 modeled epitopes as input.


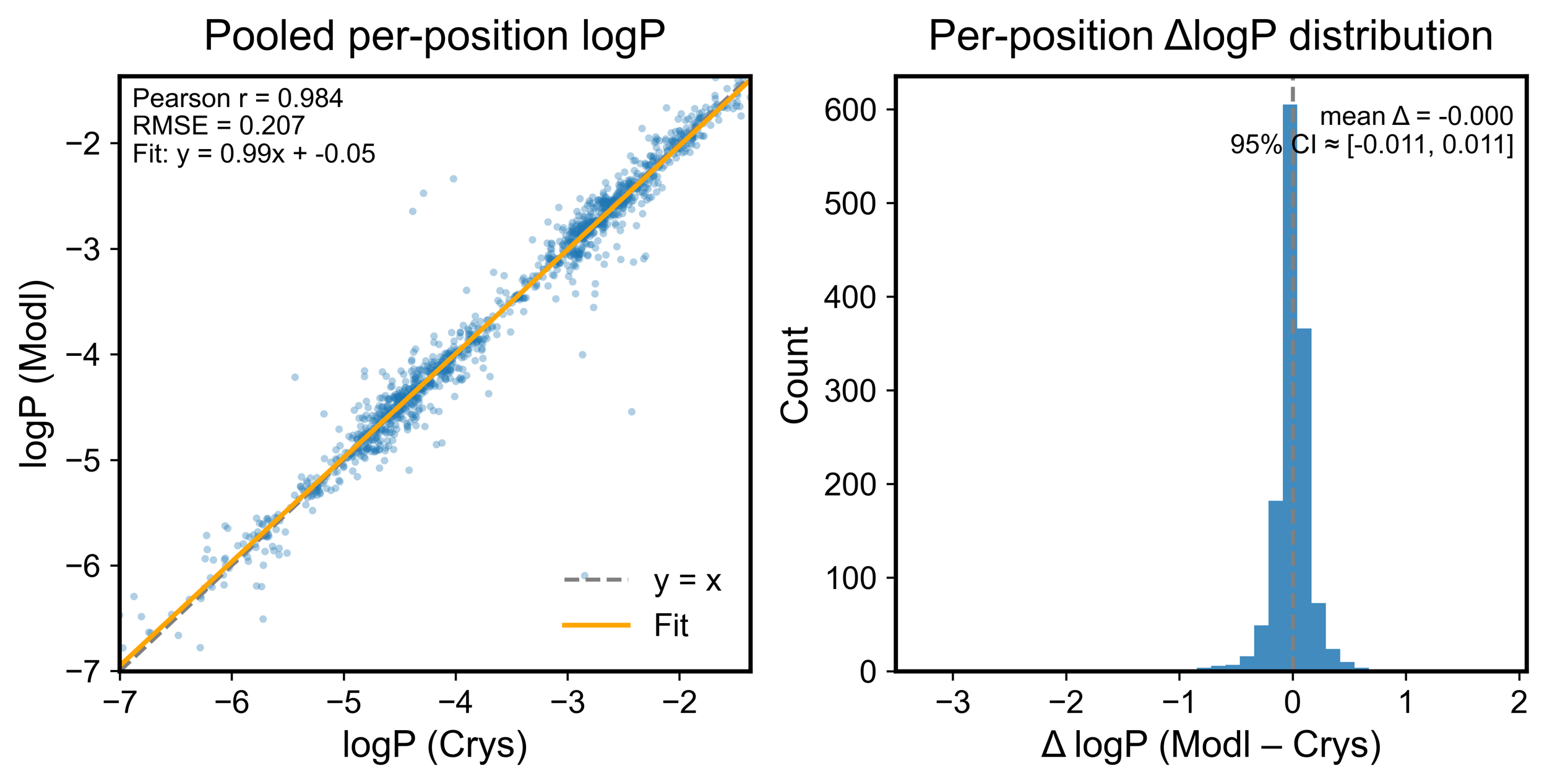


**Figure S9**. Comparison of decoder-derived per-position logP scores using crystal (Crys) versus MODELLER-generated (Modl) structures. The left panel is the scatter plot of pooled per-position logP scores for 103 epitope pairs, with identity line (grey dashed) and linear fit (orange) shown; Pearson’s r and RMSE indicate near-perfect concordance. The right panel is the histogram of per-position ΔlogP (Modl − Crys) values. The mean ΔlogP(Modl-Crys) and its approximate 95% confidence interval are annotated.


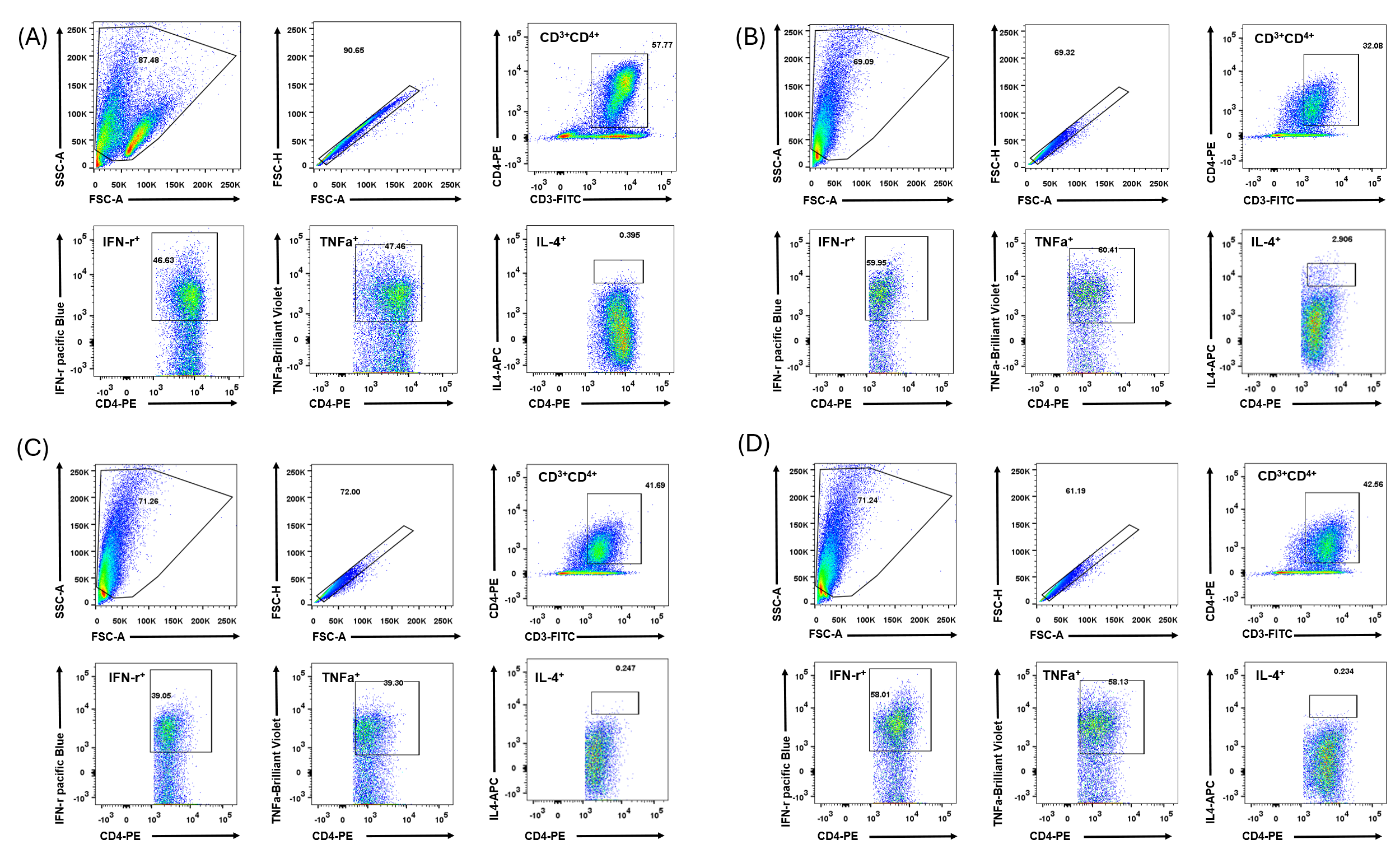


**Figure S10**. In vitro validation of WT and MUT (Origin) peptides. (A) Negative control group (without PMA or BFA): Following long-term stimulation with 50 IU/mL IL-2, the frequencies of IFN-γ⁺ and TNF-α⁺ cells in PBMCs were significantly increased, while IL-4⁺ cells were barely detectable. These findings are consistent with previously reported results. (B) Positive control group (with PMA and BFA added prior to flow cytometry analysis): Following long-term stimulation with 50 IU/mL IL-2 and WT-peptide, and after addition of PMA (25 ng/mL) and Brefeldin A (5 μg/mL) before analysis, the frequencies of IFN-γ⁺ and TNF-α⁺ cells in PBMCs were significantly higher than those in the negative control group. A slight increase in IL-4⁺ cells was also observed, accounting for 2.906%. (C) Following long-term stimulation of PBMCs with WT-peptide, the frequency of IFN-γ⁺ cells was 39.05%, and that of TNF-α⁺ cells was 39.30%. In contrast to the WT-peptide-stimulated group, IL-4⁺ cells were barely detectable. (D) Following long-term stimulation of PBMCs with MUT-peptide, the frequency of IFN-γ⁺ cells was 58.01%, and that of TNF-α⁺ cells was 58.13%, indicating a marked increase compared to the WT-peptide-stimulated group. IL-4⁺ cells remained barely detectable.


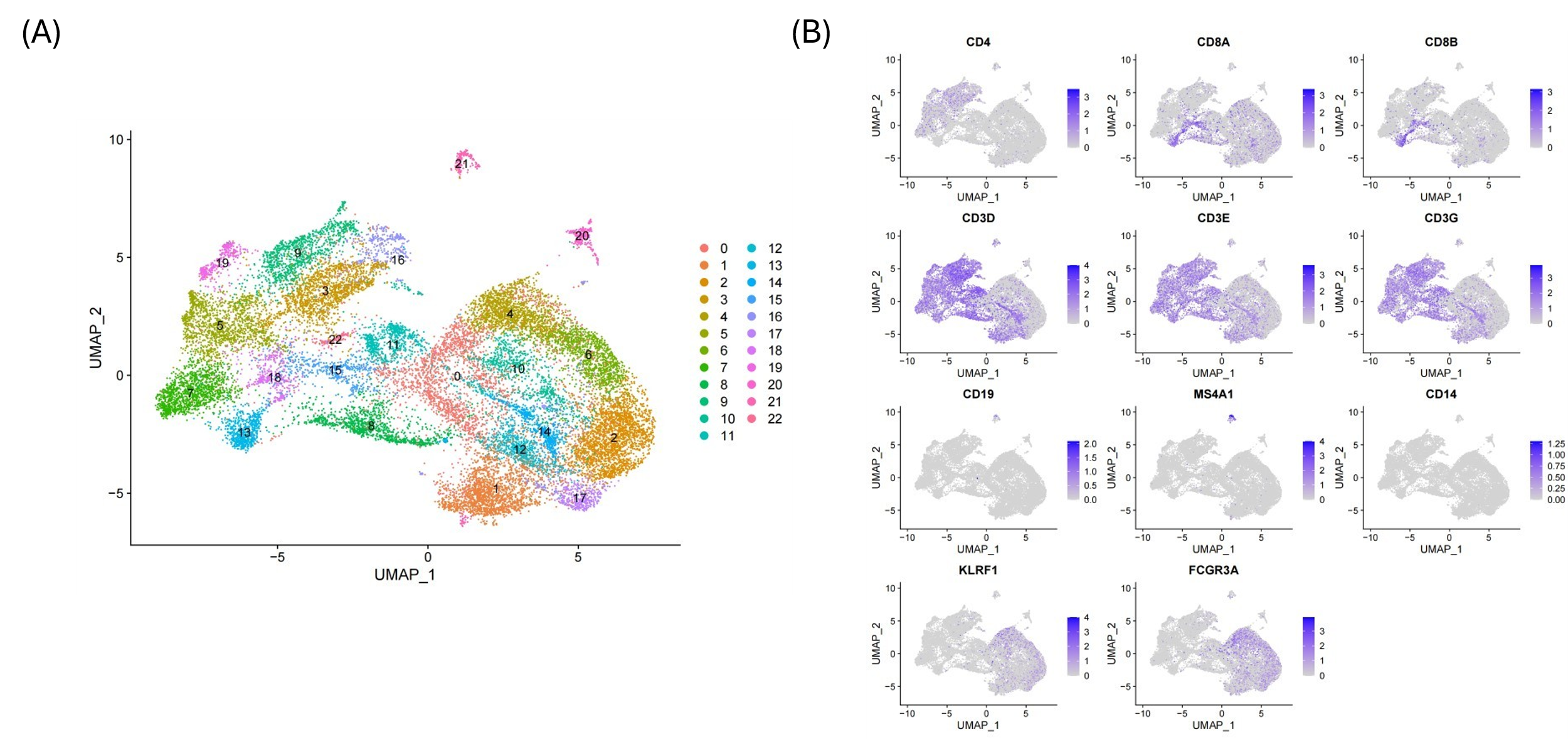


**Figure S11**. (A) All cell clusterings in PBMCs. The CD4-positive cell clusterings include six clusters: 3, 5, 7, 9, 16, and 19. (B) Expression patterns of marker genes in cell clusterings. This figure shows the expression of CD4 (T cell marker), CD8A (T cell marker), CD8B (T cell marker), CD3D (T cell marker), CD3E (T cell marker), CD3G (T cell marker), CD19 (B cell marker), MS4A1 (B cell marker), CD14 (monocyte/macrophage marker), KLRF1 (NK cell marker), and FCGR3A (NK cell & macrophage marker).


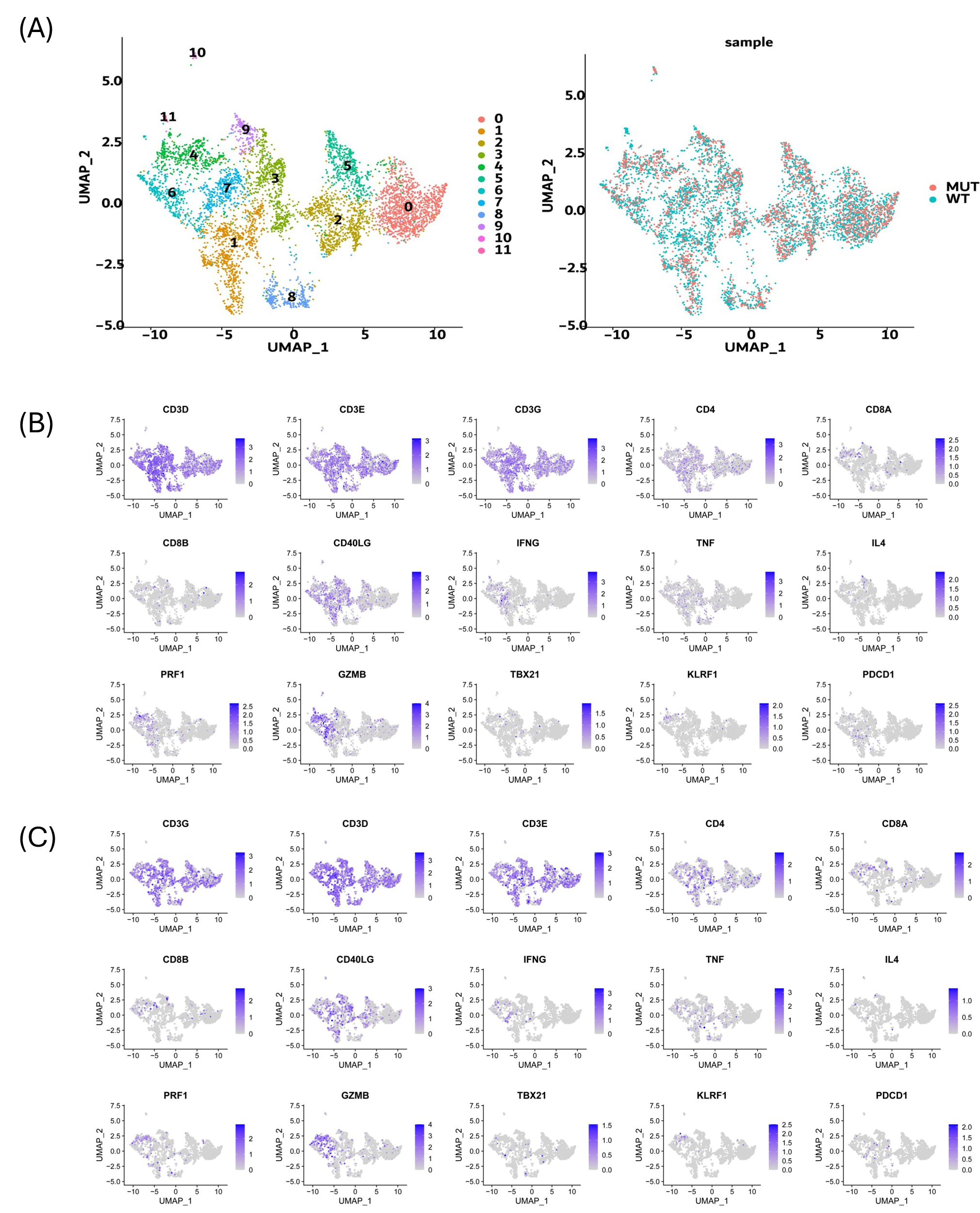


**Figure S12**. (A) Cell clustering of MUT-peptide-stimulated group and WT-peptide-stimulated group. The cell clustering patterns of the MUT-peptide-stimulated group and the WT-peptide-stimulated group are consistent, demonstrating that they originate from the same individual. (B) Expression of genes associated with T cells (CD3D+, CD3E+, CD3G+, CD4+, CD8A-, CD8B-, CD4+), activated T cells (CD40L+, IFNG+, TNF+, IL4+, PRF1+, GZMB+, TBX21+), and exhaustion caused by long-term culture (KLRF1+, PDCD1+) in the MUT-peptide-stimulated group. (C) Expression of genes associated with T cells (CD3D+, CD3E+, CD3G+, CD4+, CD8A-, CD8B-, CD4+), activated T cells (CD40L+, IFNG+, TNF+, IL4+, PRF1+, GZMB+, TBX21+), and exhaustion caused by long-term culture (KLRF1+, PDCD1+) in the WT-peptide-stimulated group.


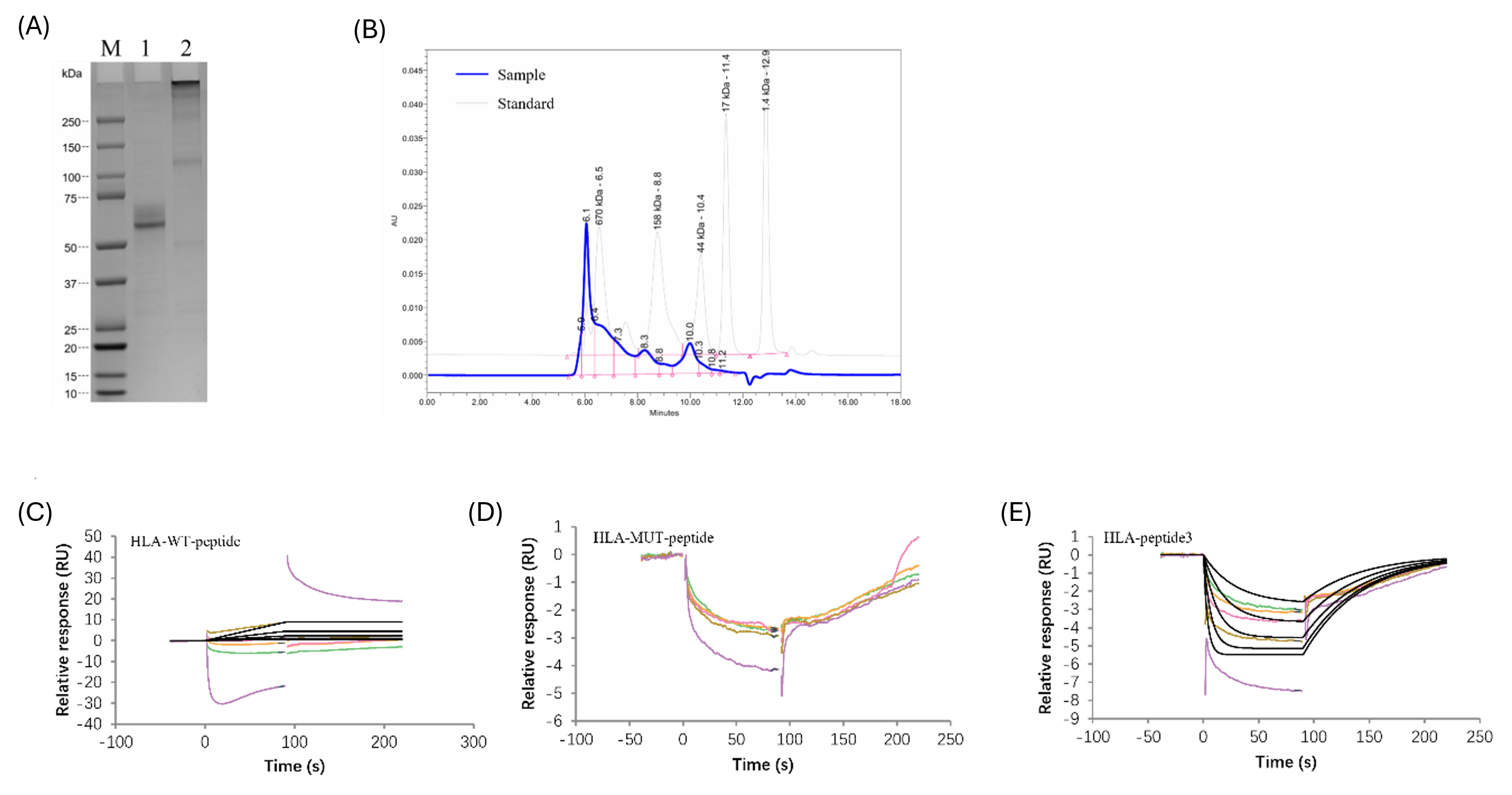


**Figure S13**. Identification of the TCR and affinity characterization of mimotope peptides. (A) SDS-PAGE identification of TCR. Where M is Marker, lane 1 was Reducing SDS-PAGE, and lane 2 was Non-reducing SDS-PAGE. There were only a few heterodimers of the TCR. (B) SEC-HPLC plot of TCR. The TCR was mostly aggregated. (C) SPR affinity test of HLA-WT-peptide to TCR. (D) SPR affinity test of HLA-MUT-peptide and TCR. (E) SPR affinity test of HLA-peptide3(P4) with TCR. The KD value could not be fitted to the above data.

The code for running MCCS scoring technique:

docker pull stcmz/mccs

docker run --rm -it -v "$(pwd):/data" stcmz/mccs

cd /data

chimera --nogui --script ~/incompleteSideChains.py 1aqd_MHC_II.pdb # Fix protein side chains.

vega 1aqd_MHC_II.pdb -o 1aqd_MHC_II.pdbqt -f VINA -c Gasteiger -p VINA -l GEN -r APOLAR -w # Convert MHC-II to PDBQT format.

vega 1aqd_epitope.pdb -o 1aqd_epitope.pdbqt -f VINA -c Gasteiger -p VINA -l GEN -r APOLAR -w # Convert epitope to PDBQT format.

pdbqtf $(grep --include=*.pdbqt -rl '?') -a # Fix problematic PDBQT files.

jdock -r 1aqd_MHC_II.pdbqt -l 1aqd_epitope.pdbqt -o output_MHCII -spa # Score the residues on the MHC-II binding groove.

jdock -r 1aqd_epitope.pdbqt -l 1aqd_MHC_II.pdbqt -o output_epitope -spa # Score the residues on the Epitope.

**Supplementary Tables**

**Table S1**. The ranking of the most popular residues* on MHC-II binding groove with high energy contributions to the binding at each position calculated by MCCS as a supplementary of Figure S2F. *For example, 107 ‘HIS81’ on beta chains of 133 MHC-II strongly interact with the epitope residues at position one, and ‘HIS81’ is the most popular residue with which epitopes interact at this position that is shared by 107 MHC-II, including DR, DP, and DQ. 90 SER53 in HLA-DR or HLA-DP encoded G-alpha chains out of 133 MHC-II strongly interact with the epitope residues at position two. At the same position as SER53, ARG52 in HLA-DQ encoded G-alpha chains only have 22 complexes in total (out of 133). Thus, the ARG52 does not appear in the top 10 popular residues of position 2.

**Table S2**. The comparison of RMSD of the epitope structures modeled/predicted by Modeller, RF2, AF2 and AF3.

| Protein | RMSD-Modeller | RMSD-RF2 | RMSD-AF2 | RMSD-AF3 |
| --- | --- | --- | --- | --- |
| 1aqd | 0.185979 | 5.618262 | 6.825874 | 7.49762 |
| 1bx2 | 0.209999 | 3.107634 | 7.413674 | 7.17539 |
| 1dlh | 0.402396 | 5.00735 | 2.367344 | 7.217168 |
| 1fv1 | 2.7719 | 3.157023 | 8.762321 | 11.21175 |
| 1fyt | 0.256167 | 3.708356 | 2.389387 | 7.08383 |
| 1h15 | 0.124642 | 4.891128 | 8.212379 | 8.08903 |
| 1hqr | 2.306683 | 5.544256 | 5.286612 | 6.443105 |
| 1hxy | 0.095933 | 3.568596 | 2.30262 | 7.178131 |
| 1j8h | 0.568528 | 3.247761 | 2.492006 | 7.098818 |
| 1jk8 | 0.140275 | 7.134694 | 7.175263 | 7.646028 |
| 1jwm | 0.368312 | 4.869128 | 2.331051 | 7.042154 |
| 1jws | 0.293946 | 4.885292 | 2.306039 | 7.087716 |
| 1jwu | 0.263832 | 4.480951 | 2.333447 | 7.032641 |
| 1kg0 | 0.255376 | 3.621991 | 2.288856 | 7.13322 |
| 1klg | 0.194389 | 4.979249 | 3.108725 | 8.659452 |
| 1klu | 0.281456 | 4.825514 | 2.426484 | 13.17553 |
| 1lo5 | 0.457394 | 4.131426 | 2.268888 | 7.111189 |
| 1r5i | 0.377569 | 4.566039 | 2.695035 | 7.083488 |
| 1s9v | 0.137471 | 3.121579 | 1.963096 | 1.901417 |
| 1sje | 0.159568 | 4.814055 | 2.914406 | 6.698822 |
| 1sjh | 1.611107 | 7.071011 | 2.637045 | 7.121159 |
| 1t5w | 0.175792 | 3.646556 | 2.433337 | 7.314319 |
| 1t5x | 0.268742 | 6.755722 | 2.327486 | 7.327592 |
| 1uvq | 0.174232 | 8.561465 | 2.09102 | 7.451432 |
| 1ymm | 1.126642 | 2.980112 | 7.717927 | 7.161184 |
| 1zgl | 1.952302 | 3.659951 | 7.390831 | 8.298331 |
| 2g9h | 0.357484 | 4.288024 | 2.466566 | 7.072738 |
| 2iam | 0.449877 | 4.221358 | 2.722189 | 8.993349 |
| 2ian | 0.471913 | 5.071945 | 3.017722 | 12.27278 |
| 2icw | 0.370394 | 2.675802 | 2.674626 | 7.087987 |
| 2nna | 0.151366 | 2.682457 | 2.660636 | 2.412754 |
| 2oje | 0.343982 | 2.951778 | 2.695947 | 7.037703 |
| 2q6w | 0.220703 | 4.306757 | 1.557984 | 6.817453 |
| 2seb | 0.154659 | 1.889441 | 2.031221 | 6.377065 |
| 2xn9 | 0.313487 | 3.224238 | 2.424323 | 6.889258 |
| 3c5j | 0.137956 | 4.530612 | 8.261694 | 6.929771 |
| 3pl6 | 1.759788 | 11.1896 | 7.84217 | 8.060941 |
| 3s4s | 0.266712 | 4.367556 | 2.305901 | 7.032 |
| 3s5l | 0.269834 | 3.182812 | 2.428766 | 6.963082 |
| 3wex | 0.185738 | 5.027005 | 2.199799 | 2.3814 |
| 4c56 | 0.410535 | 5.050604 | 2.34337 | 7.139302 |
| 4e41 | 0.33681 | 3.330436 | 3.173441 | 8.341903 |
| 4fqx | 0.174778 | 2.985103 | 1.257271 | 1.165533 |
| 4gbx | 1.400374 | 6.063892 | 1.327377 | 6.22908 |
| 4gg6 | 0.461004 | 3.042202 | 2.520302 | 2.871821 |
| 4h1l | 0.246717 | 6.19121 | 1.473504 | 3.983451 |
| 4h25 | 0.181721 | 5.096059 | 4.259409 | 8.601008 |
| 4h26 | 1.971331 | 8.113893 | 1.628064 | 3.314446 |
| 4i5b | 0.204029 | 6.446402 | 1.379902 | 6.909415 |
| 4is6 | 0.137935 | 6.278128 | 3.652652 | 7.359462 |
| 4may | 2.396289 | 6.924302 | 7.268713 | 7.252786 |
| 4mdj | 0.114664 | 9.05239 | 0.966581 | 5.776282 |
| 4ov5 | 0.406667 | 6.510583 | 7.533945 | 7.493715 |
| 4ozf | 2.687459 | 2.257969 | 1.810695 | 3.08757 |
| 4ozg | 0.401959 | 1.919023 | 1.741479 | 2.401679 |
| 4ozh | 0.336772 | 2.53702 | 2.016623 | 1.962085 |
| 4ozi | 0.505608 | 3.226993 | 1.630589 | 1.806385 |
| 4p4k | 2.207042 | 5.064146 | 6.983349 | 6.334688 |
| 4p4r | 2.114128 | 6.444559 | 7.162475 | 6.446213 |
| 4p57 | 0.23741 | 6.828715 | 7.975353 | 8.126889 |
| 4p5k | 0.263953 | 7.776086 | 3.264848 | 7.862395 |
| 4p5m | 0.26568 | 6.022577 | 2.162135 | 8.135264 |
| 4y19 | 0.984961 | 6.664767 | 2.470159 | 7.307184 |
| 4y1a | 0.140668 | 6.821829 | 2.493221 | 7.974181 |
| 4z7u | 3.477167 | 2.875228 | 2.717875 | 4.503829 |
| 4z7v | 0.509378 | 3.295281 | 2.552632 | 2.923472 |
| 4z7w | 2.634653 | 2.187194 | 2.672361 | 3.359954 |
| 5ks9 | 3.349783 | 4.2007 | 2.670831 | 4.965213 |
| 5ksa | 0.61673 | 4.2789 | 2.432858 | 2.734763 |
| 5ksb | 2.315518 | 3.448184 | 2.180261 | 2.754206 |
| 5lax | 0.249454 | 6.356277 | 2.343439 | 7.138725 |
| 5ni9 | 0.148398 | 8.152593 | 7.253471 | 7.769891 |
| 5ujt | 0.378529 | 6.688486 | 1.572214 | 7.485831 |
| 5v4m | 0.10301 | 4.634462 | 2.485747 | 6.558744 |
| 5v4n | 0.116078 | 5.115614 | 6.408973 | 6.432277 |
| 6atf | 0.109942 | 6.289464 | 2.450579 | 7.413313 |
| 6biy | 0.139339 | 7.252109 | 7.822804 | 7.74727 |
| 6cpl | 0.173528 | 7.775503 | 8.753879 | 8.516582 |
| 6cpn | 1.501229 | 5.761908 | 7.56821 | 7.659748 |
| 6cpo | 1.328208 | 6.111344 | 8.302152 | 7.937728 |
| 6cqj | 1.330428 | 6.447186 | 7.589583 | 7.657064 |
| 6cql | 1.516946 | 6.647014 | 7.797343 | 7.608296 |
| 6cqn | 1.499898 | 6.592392 | 7.831405 | 7.617397 |
| 6cqq | 1.452857 | 6.371414 | 7.879473 | 7.365623 |
| 6cqr | 1.538332 | 6.003054 | 7.960569 | 7.656935 |
| 6dfx | 0.303503 | 8.435847 | 1.666138 | 7.767692 |
| 6dig | 0.162853 | 5.783126 | 7.005927 | 7.196171 |
| 6hby | 0.22823 | 8.927827 | 7.974462 | 8.112255 |
| 6mff | 0.166937 | 2.527684 | 1.355838 | 2.013114 |
| 6mfg | 0.215673 | 1.318735 | 1.157913 | 1.669637 |
| 6nix | 0.127912 | 9.253143 | 2.551211 | 11.35604 |
| 6px6 | 0.528095 | 4.290643 | 2.54164 | 9.575077 |
| 6py2 | 0.14072 | 4.579253 | 1.902588 | 9.049367 |
| 6u3m | 0.176215 | 3.798581 | 1.000861 | 1.441091 |
| 6u3n | 0.270997 | 1.920321 | 1.24244 | 1.34194 |
| 6u3o | 0.145119 | 5.546123 | 2.211792 | 3.755308 |
| 6xc9 | 0.565366 | 7.561249 | 3.047067 | 8.004852 |
| 6xco | 3.944997 | 7.86391 | 2.27256 | 9.799211 |
| 6xcp | 0.266232 | 7.23752 | 2.900763 | 7.958385 |
| 6xp6 | 0.656266 | 3.728769 | 1.764498 | 3.694968 |
| 7kei | 0.121903 | 9.112164 | 5.941873 | 2.195462 |
| 7n19 | 0.321839 | 5.492351 | 1.626137 | 10.53598 |
| ave | 0.71 | 5.16 | 3.76 | 6.55 |

**Table S3**. The detail information of 133 co-crystalized MHC-II epitopes on PDB. (* indicates the modified residues, such as CIR) The ‘102 Benchmark’ column records whether the epitopes listed are also included in the 102 epitopes for comparing performance among MODELLER, RF2, and AF2 (AF3). The last column records which co-crystalized epitopes are chosen to be the 55 templates of MODELLER, shown as True in that row.

| peptide seq | PDB | antigen seq | cut range | length | antigen name | MHC-II allele | 102 Benchmark | MODELLER-tempate |
| --- | --- | --- | --- | --- | --- | --- | --- | --- |
| VGSDWRFLRGYHQYA | 1aqd | IMYGCDVGSDGRFLRGYRQDAYDGKDYIAL | 127-141 | 13 | P04439 | MH2 HLA-DRA*0101_HLA-DRB1*0101 | TRUE | TRUE |
| ENPVVHFFKNIVTPR | 1bx2 | HGRTQDENPVVHFFKNIVTPRTPPPSQGKG | 217-231 | 15 | P02686 | MH2 HLA-DRA*0101_HLA-DRB1*1501 | TRUE | TRUE |
| **RAM*SL* | 1d5m |  |  | 9 | no info | MH2 HLA-DRA*0101_HLA-DRB1*0401 |  |  |
| **R*** | 1d5x |  |  | 6 | no info |  |  |  |
| **RA*SL* | 1d5z |  |  | 8 | no info | MH2 HLA-DRA*0101_HLA-DRB1*0401 |  |  |
| **R*MAS** | 1d6e |  |  | 9 | no info | MH2 HLA-DRA*0101_HLA-DRB1*0401 |  |  |
| PKYVKQNTLKLAT | 1dlh | DKPFQNVNKITYGACPKYVKQNTLKLATGMRNVPEKQT | 306-318 | 13 | P04664 | MH2 HLA-DRA*0101_HLA-DRB1*0101 | TRUE |  |
| NPVVHFFKNIVTPRTPPPSQ | 1fv1 | HGRTQDENPVVHFFKNIVTPRTPPPSQGKGRGLSLSRFSW | 218-237 | 20 | P02686 | MH2 HLA-DRA*0101_HLA-DRB5*0101 | TRUE | TRUE |
| PKYVKQNTLKLAT | 1fyt | DKPFQNVNKITYGACPKYVKQNTLKLATGMRNVP | 322-334 | 13 | P03437 | MH2 HLA-DRA*0101_HLA-DRB1*0101 | TRUE |  |
| GGVYHFVKKHVHES | 1h15 | YESFRLTGGVYHFVKKHVHESFLASLLTSW | 628-641 | 14 | P03198 | MH2 HLA-DRA*0101_HLA-DRB5*0101 | TRUE | TRUE |
| VHFFKNIVTPRTP | 1hqr | HGRTQDENPVVHFFKNIVTPRTPPPSQGKG | 221-233 | 13 | P02686 | MH2 HLA-DRA*0101_HLA-DRB5*0101 | TRUE | TRUE |
| PKYVKQNTLKLAT | 1hxy | ITYGACPKYVKQNTLKLATGMRNVPEKQIR | 327-339 | 13 | Q03909 | MH2 HLA-DRA*0101_HLA-DRB1*0101 | TRUE | TRUE |
| PKYVKQNTLKLAT | 1j8h | QNVNKITYGACPKYVKQNTLKLATGMRNVP | 322-334 | 13 | P03437 | MH2 HLA-DRA*0101_HLA-DRB1*0401 | TRUE |  |
| LVEALYLVCGERGG | 1jk8 | CGSHLVEALYLVCGERGFFYTPKTRREAED | 35-47 | 13 | P01308 | MH2 HLA-DQA1*0302_HLA-DQB1*0302 | TRUE | TRUE |
| PKYVKQNTLKLAT | 1jwm | QNVNKITYGACPKYVKQNTLKLATGMRNVP | 322-334 | 13 | P03437 | MH2 HLA-DRA*0101_HLA-DRB1*0101 | TRUE |  |
| PKYVKQNTLKLAT | 1jws | QNVNKITYGACPKYVKQNTLKLATGMRNVP | 322-334 | 13 | P03437 | MH2 HLA-DRA*0101_HLA-DRB1*0101 | TRUE |  |
| PKYVKQNTLKLAT | 1jwu | QNVNKITYGACPKYVKQNTLKLATGMRNVP | 322-334 | 13 | P03437 | MH2 HLA-DRA*0101_HLA-DRB1*0101 | TRUE |  |
| PKYVKQNTLKLAT | 1kg0 | QNVNKITYGACPKYVKQNTLKLATGMRNVP | 322-334 | 13 | P03437 | MH2 HLA-DRA*0101_HLA-DRB1*0101 | TRUE |  |
| GELIGILNAAKVPAD | 1klg | SLGELIGTLNAAKVPADTEVVCAPPTAYID | 23-37 | 15 | P60174 | MH2 HLA-DRA*0101_HLA-DRB1*0101 | TRUE | TRUE |
| GELIGTLNAAKVPAD | 1klu | SLGELIGTLNAAKVPADTEVVCAPPTAYID | 23-37 | 15 | P60174 | MH2 HLA-DRA*0101_HLA-DRB1*0101 | TRUE |  |
| PKYVKQNTLKLAT | 1lo5 | QNVNKITYGACPKYVKQNTLKLATGMRNVP | 322-334 | 13 | P03437 | MH2 HLA-DRA*0101_HLA-DRB1*0101 | TRUE |  |
| *FVKQNA*AL | 1pyw | CPKYVKQNTLKLATGMRNVP | 324-332 | 10 | P03437 | MH2 HLA-DRA*0101_HLA-DRB1*0101 |  |  |
| PKYVKQNTLKLAT | 1r5i | TYGACPKYVKQNTLKLATGMRNVP | 306-318 | 13 | P11133 |  | TRUE |  |
| LQPFPQPELPY | 1s9v | SQLPYLQLQPFPQPQLPYSQPQPFRPQQPY | 78-88 | 11 | P02863 | MH2 HLA-DQA1*0501_HLA-DQB1*0201 | TRUE | TRUE |
| AAAAAAAAAAAAA | 1seb | no |  | 13 |  | MH2 HLA-DRA*0101_HLA-DRB1*0101 |  |  |
| PEVIPMFSALSEGATP | 1sje | EEKAFSPEVIPMFSALSEGATPQDLNTMLN | 167-182 | 16 | P12495 | MH2 HLA-DRA*0101_HLA-DRB1*0101 | TRUE | TRUE |
| PEVIPMFSALSEG | 1sjh | EEKAFSPEVIPMFSALSEGATPQDLNTMLN | 167-179 | 13 | P12495 | MH2 HLA-DRA*0101_HLA-DRB1*0101 | TRUE |  |
| AAYSDQATPLLLSPR | 1t5w | SLSTTPLLLSPRVNMINTAI | 455-462 | 5 | P27705 | MH2 HLA-DRA*0101_HLA-DRB1*0101 | TRUE | TRUE |
| AAYSDQATPLLLSPR | 1t5x | SLSTTPLLLSPRVNMINTAI | 455-462 | 15 | P27705 | MH2 HLA-DRA*0101_HLA-DRB1*0101 | TRUE |  |
| EGRDSMNLPSTKVSWAAVGGGGSLVPRGSGGGG | 1uvq | MNLPSTKVSWAAVTLLLLLL | 13-Jan | 33 | O43612 | MH2 HLA-DQA1*0102_HLA-DQB1*0602 | TRUE | TRUE |
| ENPVVHFFKNIVTPRGGSGGGGG | 1ymm | HGRTQDENPVVHFFKNIVTPRTPPPSQGKGRGLSLS | 217-239 | 23 | P02686 | MH2 HLA-DRA*0101_HLA-DRB1*1501 | TRUE |  |
| VHFFKNIVTPRTP | 1zgl | HGRTQDENPVVHFFKNIVTPRTPPPSQGKG | 221-233 | 13 | P02686 | MH2 HLA-DRA*0101_HLA-DRB5*0101 | TRUE |  |
| PKYVKQNTLKLAT | 2g9h | TYGACPKYVKQNTLKLATGMRNVPEKQT | 306-318 | 13 | P04664 | MH2 HLA-DRA*0101_HLA-DRB1*0101 | TRUE |  |
| GELIGILNAAKVPAD | 2iam | SLGELIGTLNAAKVPADTEVVCAPPTAYID | 23-37 | 15 | P60174 | MH2 HLA-DRA*0101_HLA-DRB1*0101 | TRUE |  |
| GELIGTLNAAKVPAD | 2ian | SLGELIGTLNAAKVPADTEVVCAPPTAYID | 23-37 | 15 | P60174 | MH2 HLA-DRA*0101_HLA-DRB1*0101 | TRUE |  |
| PKYVKQNTLKLAT | 2icw |  |  | 13 | no info | MH2 HLA-DRA*0101_HLA-DRB1*0101 | TRUE |  |
| *PK*VKQNTLKLAT | 2ipk | NKITYGACPKYVKQNTLKLATGMRNVPEKQ |  | 14 | A8CDU0 | MH2 HLA-DRA_HLA-DRB1-1 |  |  |
| QQYPSGEGSFQPSQENPQ | 2nna | PQQQYPSGQGSFQPSQQNPQAQGSVQPQQL | 243-260 | 18 | P18573 | MH2 HLA-DQA1*0301_HLA-DQB1*0302 | TRUE | TRUE |
| PKYVKQNTLKLAT | 2oje | VNRITYGACPKYVKQNTLKLATGMRNVPEK | 260-272 | 13 | Q91LS8 | MH2 HLA-DRA*0101_HLA-DRB1*0101 | TRUE |  |
| AWRSDEALPLGS | 2q6w | QCLAVSPMCAWCSDEALPLGSPRCDLKENL | 50-61 | 12 | P05106 | MH2 HLA-DRA*0101_HLA-DRB1*0101 | TRUE | TRUE |
| AYMRADAAAGGA | 2seb | KGPDPLQYMRADQAAGGLRQHDAEVDATLK | 1238-1247 | 12 | P02458 | MH2 HLA-DRA*0101_HLA-DRB1*0401 | TRUE | TRUE |
| PKYVKQNTLKLAT | 2xn9 | ITYGACPKYVKQNTLKLATGMRNVPEKQIR | 327-339 | 13 | Q03909 | MH2 HLA-DRA*0101_HLA-DRB1*0101 | TRUE |  |
| QVIILNHPGQISA | 3c5j | SDPPQEAAQFTSQVIILNHPGQISAGYSPV | 343-355 | 13 | Q05639 | MH2 HLA-DRA*0101_HLA-DRB3*0301 | TRUE | TRUE |
| APPAYEKL*AEQSPP | 3l6f | KNCEPVVPNAPPAYEKLSAEQSPPPYSP | 100-114 | 15 | Q16655 | MH2 HLA-DRA*0101_HLA-DRB1*0101 |  |  |
| NPVVHFFKNIVTPR | 3pl6 |  |  |  | no info | MH2 HLA-DQA1*0102_HLA-DQB1*0501 | TRUE |  |
| PKYVKQNTLKLAT | 3s4s | QNVNKITYGACPKYVKQNTLKLATGMRNVP | 322-334 | 13 | P03437 | MH2 HLA-DRA*0101_HLA-DRB1*0101 | TRUE |  |
| PKYVKQNTLKLAT | 3s5l | QNVNKITYGACPKYVKQNTLKLATGMRNVP | 322-334 | 13 | P03437 | MH2 HLA-DRA*0101_HLA-DRB1*0101 | TRUE |  |
| KVTVAFNQF | 3wex |  | 214-222 | 9 | no info | MH2 HLA-DPA1*0202_HLA-DPB1*0501 | TRUE | TRUE |
| PKYVKQNTLKLAT | 4c56 | QNVNKITYGACPKYVKQNTLKLATGMRNVP | 322-334 | 13 | P03437 | MH2 HLA-DRA*0101_HLA-DRB*0101 | TRUE |  |
| GELIGILNAAKVPAD | 4e41 | SLGELIGTLNAAKVPADTEVVCAPPTAYID | 23-37 | 15 | P60174 | MH2 HLA-DRA*0101_HLA-DBR1*0101 | TRUE |  |
| GKQNCLKLATK | 4fqx |  |  | 11 | no |  | TRUE | TRUE |
| GKQNCLKLAT | 4gbx |  |  | 10 | no |  | TRUE |  |
| QQYPSGEGSFQPSQENPQ | 4gg6 | PQQQYPSGQGSFQPSQQNPQAQGSVQPQQL | 243-260 | 18 | P18573 | MH2 HLA-DQA1*0301_HLA-DQB1*0302 | TRUE |  |
| QHIRCNIPKRISA | 4h1l |  |  | 13 | no | MH2 HLA-DRA*0101_HLA-DRB3*0301 | TRUE | TRUE |
| QHIRCNIPKRIGPSKVATLVPR | 4h25 |  |  | 22 | no | MH2 HLA-DRA*0101_HLA-DRB3*0301 | TRUE | TRUE |
| QWIRVNIPKRI | 4h26 |  |  | 11 | no |  | TRUE |  |
| VVKQNCLKLATK | 4i5b |  |  | 12 | no info | MH2 HLA-DRA1_HLA-DRB1 | TRUE | TRUE |
| WNRQLYPEWTEAQRLD | 4is6 | TKAWNRQLYPEWTEAQRLDCWRGGQVSLKV | 44-59 | 16 | P40967 | MH2 HLA-DRA*0101_HLA-DRB1*0401 | TRUE | TRUE |
| QLVHFVRDFAQL | 4may |  |  | 12 | no info | MH2 HLA-DQA1*0102_HLA-DQB1*0501 | TRUE | TRUE |
| SAVRL*SSVPGVR | 4mcy | YATRSSAVRLRSSVPGVRLLQDSVDFSLAD | 66-78 | 13 | P08670 | MH2 HLA-DRA*0101_HLA-DRB1-4 |  |  |
| GVYAT*SSAVRLR | 4mcz | LYASSPGGVYATRSSAVRLRSSVPGVRLL | 59-71 | 13 | P08670 | MH2 HLA-DRA*0101_HLA-DRB1-4 |  |  |
| GVYAT*SSAV*L* | 4md0 | LYASSPGGVYATRSSAVRLRSSVPGVRLL | 59-71 | 13 | P08670 | MH2 HLA-DRA*0101_HLA-DRB1-4 |  |  |
| ATEY*V*VNSAYQDK | 4md4 | EKEVVLLVATEGRVRVNSAYQDKVSLPNYP | 89-103 | 15 | P16112 | MH2 HLA-DRA*0101_HLA-DRB1-4 |  |  |
| SAVRL*SSVPGVR | 4md5 | YATRSSAVRLRSSVPGVRLLQDSVDFSLAD | 66-78 | 13 | P08670 | MH2 HLA-DRA*0101_HLA-DRB1-4 |  |  |
| SAVRL*SSVPGVR | 4mdi | YATRSSAVRLRSSVPGVRLLQDSVDFSLAD | 66-78 | 13 | P08670 | MH2 HLA-DRA*0101_HLA-DRB1-4 |  |  |
| SAVRLRSSVPGVR | 4mdj | YATRSSAVRLRSSVPGVRLLQDSVDFSLAD | 66-78 | 13 | P08670 | MH2 HLA-DRA*0101_HLA-DRB1 | TRUE | TRUE |
| GSDARFLRGYHLYA | 4ov5 | IMYGCDVGSDGRFLRGYRQDAYDGKDYIAL | 128-141 | 14 | P04439 | MH2 HLA-DRA*0101_HLA-DRB1*0101 | TRUE | TRUE |
| APQPELPYPQPGS | 4ozf | FPQPQLPYPQPQPFRPQQPY | 82-91 | 13 | P04722 | MH2 HLA-DQA1*0508_HLA-DQB1*0201 | TRUE |  |
| APQPELPYPQPGS | 4ozg | FPQPQLPYPQPQPFRPQQPY | 82-91 | 13 | P04722 | MH2 HLA-DQA1*0501_HLA-QB1*0201 | TRUE |  |
| APQPELPYPQPGS | 4ozh | FPQPQLPYPQPQPFRPQQPY | 82-91 | 13 | P04722 | MH2 HLA-DQA1*0501_HLA-QB1*0201 | TRUE | TRUE |
| QPFPQPELPYPGS | 4ozi | SQQPYLQLQPFPQPQLPYPQPQPFRPQQPY | 79-89 | 13 | P04722 | MH2 HLA-DQA1*0501_HLA-QB1*0201 | TRUE | TRUE |
| QAFWIDLFETIG | 4p4k |  |  | 13 | no info | MH2 HLA-DPA1*0103_HLA-DPB1 | TRUE |  |
| QAFWIDLFETIG | 4p4r |  |  | 13 | no info | MH2 HLA-DPA1*0103_HLA-DPB1 | TRUE |  |
| QAFWIDLFETIGGGSLV | 4p57 |  |  | 17 | no info | MH2 HLA-DPA1*0103_HLA-DPB1 | TRUE | TRUE |
| NKFDTQLFHTITGGS | 4p5k |  |  | 15 | no info | MH2 HLA-DPA1*0103_HLA-DPB1*2602 | TRUE | TRUE |
| QAYDGKDYIALKG | 4p5m |  |  | 13 | no info | MH2 HLA-DPA1*0103_HLA-DPB1 | TRUE | TRUE |
| GSLQPLALEGSLQKRG | 4y19 | GPGAGSLQPLALEGSLQKRGIVEQCCTSIC | 75-90 | 16 | P01308 | MH2 HLA-DRA1_HLA-DRB1-4 | TRUE |  |
| GSLQPLALEGSLQKRG | 4y1a | GPGAGSLQPLALEGSLQKRGIVEQCCTSIC | 75-90 | 16 | P01308 | MH2 HLA-DRA1_HLA-DRB1-4 | TRUE | TRUE |
| APSGEGSFQPSQENPQGS | 4z7u | PQQQYPSGQGSFQPSQQNPQAQGSVQPQQL | 246-260 | 18 | P18573 | MH2 HLA-DQA1*0301_HLA-DQB1*0302 | TRUE | TRUE |
| APSGEGSFQPSQENPQGS | 4z7v | PQQQYPSGQGSFQPSQQNPQAQGSVQPQQL | 246-260 | 18 | P18573 | MH2 HLA-DQA1*0301_HLA-DQB1*0302 | TRUE |  |
| APSGEGSFQPSQENPQGS | 4z7w | PQQQYPSGQGSFQPSQQNPQAQGSVQPQQL | 246-260 | 18 | P18573 | MH2 HLA-DQA1*0301_HLA-DQB1*0302 | TRUE |  |
| TSKGLF*AAVPSGAS | 5jlz | EVDLFTSKGLFRAAVPSGASTGIYEALELR | 26-40 | 15 | P06733 | MH2 HLA-DRA*0101_HLA-DRB1*0401 |  |  |
| APSGEGSFQPSQENPQ | 5ks9 | PQQQYPSGQGSFQPSQQNPQAQGSVQPQQL | 246-260 | 16 | P18573 | MH2 HLA-DQA1*0301_HLA-DQB1*0302 | TRUE |  |
| QPQQSFPEQEA | 5ksa | SQPQQQFPQPQQPQQSFPQQQPPFIQPSLQ | 132-141 | 11 | P08079 | MH2 HLA-DQA1*0501_HLA-DQB1*0302 | TRUE | TRUE |
| GPQQSFPEQEA | 5ksb | SQPQQQFPQPQQPQQSFPQQQPPFIQPSLQ | 133-141 | 11 | P08079 | MH2 HLA-DQA1*0501_HLA-DQB1*0302 | TRUE |  |
| TSKGLFRAAVPSGAS | 5lax | EVDLFTSKGLFRAAVPSGASTGIYEALELR | 26-40 | 15 | P06733 | MH2 HLA-DRA*0101_HLA-DRB1-4 | TRUE | TRUE |
| KRIAKAVNEKSCNCL | 5ni9 | TVTNPKRIAKAVNEKSCNCLLLKVNQIGSV | 326-340 | 15 | P06733 | MH2 HLA-DRA1_HLA-DRB1*04:01 | TRUE | TRUE |
| K*IAKAVNEKSCNCL | 5nig | TVTNPKRIAKAVNEKSCNCLLLKVNQIGSV | 326-340 | 15 | P06733 | MH2 HLA-DRB1*04:01 |  |  |
| GVEELYLVAGEEGCGG | 5ujt |  |  | 16 | no info | MH2 HLA-DQA1*0301_HLA-DQB1 | TRUE | TRUE |
| GWISLWKGFSF | 5v4m |  |  | 11 | no info | MH2 HLA-DRA1_HLA-DRB1*1501 | TRUE | TRUE |
| WISLWKGFSFGS | 5v4n |  |  | 12 | no info | MH2 HLA-DRA1_HLA-DRB1*0101 | TRUE | TRUE |
| GVYATRSSAVRLR | 6atf | SLYASSPGGVYATRSSAVRLRSSVPGVRLL | 59-71 | 13 | P08670 | MH2 HLA-DRA*0101_HLA-DRA*0102 | TRUE | TRUE |
| GVYAT*SSAVRLR | 6ati | LYASSPGGVYATRSSAVRLRSSVPGVRLL | 59-71 | 13 | P08670 | MH2 HLA-DRA*0101_HLA-DRA*0102 |  |  |
| GGYRA*PAKAAT | 6atz | PAPPPISGGGYRARPAKAAATQKKVERKAP | 69-79 | 12 | P02675 | MH2 HLA-DRA*0101_HLA-DRB1*1402 |  |  |
| GGY*A*PAKAAAT | 6bij | PAPPPISGGGYRARPAKAAATQKKVERKAP | 69-81 | 13 | P02675 | MH2 HLA-DRA1_HLA-DRB1-4 |  |  |
| GGYRA*PAKAAAT | 6bil | PAPPPISGGGYRARPAKAAATQKKVERKAP | 69-81 | 13 | P02675 | MH2 HLA-DRA1_HLA-DRB1-4 |  |  |
| QYM*ADQAAGGLR | 6bin | KGPDPLQYMRADQAAGGLRQHDAEVDATLK | 1237-1249 | 13 | P02458 | MH2 HLA-DRA1_HLA-DRB1-4 |  |  |
| SSLNL*ETNLDSL | 6bir | ISLPLPNFSSLNLRETNLDSLPLVDTHSKR | 419-431 | 13 | P08670 | MH2 HLA-DRA1_HLA-DRB1*0405 |  |  |
| ETVCP*TTQQSPE | 6biv | ISLPLPNFSSLNLRETNLDSLPLVDTHSKR | 419-431 | 13 | P08670 | MH2 HLA-DRA*0101_HLA-DRB1*0401 |  |  |
| ETVCP*TTQQSPE | 6bix | VKETVCPRTTQQSPEDCDFK | 83-95 | 13 | P49913 | MH2 HLA-DRA*0101_HLA-DRB1*0401 |  |  |
| DIFERIASEASRL | 6biy | MSIMNSFVTDIFERIASEASRLAHYSKRST | 70-82 | 13 | Q96A08 | MH2 HLA-DRA1_HLA-DRB1*0405 | TRUE | TRUE |
| NDIFE*IASEAS*LA | 6biz | GIMNSFVNDIFERIASEASRLAHYNKRSTI | 68-82 | 15 | Q8N257 | MH2 HLA-DRB1_HLA-DRB1*0405 |  |  |
| FRDYVDRFYKTLRAEQASQE | 6cpl | SILDIRQGPKEPFRDYVDRFYKTLRAEQASQEVKNWM | 293-312 | 20 | P04585 | MH2 HLA-DRA*0101_HLA-DRB1-11 | TRUE | TRUE |
| RFYKTLRAEQASQ | 6cpn | EPFRDYVDRFYKTLRAEQASQEVKNWM | 299-311 | 13 | P04585 | MH2 HLA-DRA*0101_HLA-DRB1-11 | TRUE | TRUE |
| RFYKTLRAEQASQ | 6cpo | EPFRDYVDRFYKTLRAEQASQEVKNWM | 299-311 | 13 | P04585 | MH2 HLA-DR15 | TRUE |  |
| RFYKTLRAEQASQ | 6cqj | EPFRDYVDRFYKTLRAEQASQEVKNWM | 299-311 | 13 | P04585 | MH2 HLA-DRA1_HLA-DRB1-1 | TRUE |  |
| RFYKTLRAEQASQ | 6cql | EPFRDYVDRFYKTLRAEQASQEVKNWM | 299-311 | 13 | P04585 | MH2 HLA-DRA1_HLA-DRB1-11 | TRUE |  |
| RFYKTLRAEQASQ | 6cqn | EPFRDYVDRFYKTLRAEQASQEVKNWM | 299-311 | 13 | P04585 | MH2 HLA-DRA1_HLA-DRB1-11 | TRUE |  |
| RFYKTLRAEQASQ | 6cqq | EPFRDYVDRFYKTLRAEQASQEVKNWM | 299-311 | 13 | P04585 | MH2 HLA-DR15 | TRUE |  |
| RFYKTLRAEQASQ | 6cqr | EPFRDYVDRFYKTLRAEQASQEVKNWM | 299-311 | 13 | P04585 | MH2 HLA-DR1 | TRUE |  |
| VEELYLVAGEEGCGGGGSL | 6dfx |  |  | 19 | no info | MH2 HLA-DQA1*0301 | TRUE | TRUE |
| AGNHAAGILTLGK | 6dig |  |  | 13 | no info | MH2 HLA-DQA1*0102_HLA-DQB1*0602 | TRUE | TRUE |
| ARRPPLAELAALNLSGSRL | 6hby | NQLAVLPAGAFARRPPLAELAALNLSGSRLDEVRAGA | 112-130 | 19 | Q13641 | MH2 HLA-DRA*0101_HLA-DRB1-1 | TRUE | TRUE |
| QPFPQPEQPFP | 6mff |  |  | 11 | no info | MH2 HLA-DQ2 | TRUE | TRUE |
| QPFPQPELPYP | 6mfg |  |  | 11 | no info | MH2 HLA-DQ2 | TRUE |  |
| GIAGFKGEQGPKGEP | 6nix | GPQGATGPLGPKGQTGEPGIAGFKGEQGPKGEPGPAGPQG | 459-473 | 15 | P02458 | MH2 HLA-DRA*0101_HLA-DRB1-4 | TRUE | TRUE |
| APFSEQEQPVLG | 6px6 | PILPQLPFSQQQQPVLPQQSPFSQQQLVLP | 77-86 | 12 | P16315 | MH2 HLA-DQA1_HLA-DQB1 | TRUE |  |
| APFSEQEQPVLG | 6py2 | PILPQLPFSQQQQPVLPQQSPFSQQQLVLP | 77-86 | 12 | P16315 | MH2 HLA-DQA1*0201_HLA-DQB1 | TRUE | TRUE |
| AQPMPMPELPYPGSGGSIEGR | 6u3m |  |  | 21 | no info | MH2 HLA-DQA1*0501 | TRUE | TRUE |
| APMPMPELPYPGSGGSIEGR | 6u3n |  |  | 20 | no info | MH2 HLA-DQA1*0501_HLA-DQB1*0201 | TRUE | TRUE |
| AVVQSELPYPEGSGGSIEGR | 6u3o |  |  | 20 | no info | MH2 HLA-DQA1*0501_HLA-QB1*0201 | TRUE | TRUE |
| GGY*A*PAKAAAT | 6v0y | PAPPPISGGGYRARPAKAAATQKKVERKAP | 69-81 | 13 | P02675 | MH2 HLA-DRA*0101_HLA-DRB1-4 |  |  |
| GGYRA*PAKAAAT | 6v13 | PAPPPISGGGYRARPAKAAATQKKVERKAP | 69-81 | 13 | P02675 | MH2 HLA-DRA*0101_HLA-DRB1-4 |  |  |
| GGY*A*PAKAAAT | 6v15 | PAPPPISGGGYRARPAKAAATQKKVERKAP | 69-81 | 13 | P02675 | MH2 HLA-DRA*0101_HLA-DRB1-4 |  |  |
| GGYRA*PAKAAAT | 6v18 | PAPPPISGGGYRARPAKAAATQKKVERKAP | 69-81 | 13 | P02675 | MH2 HLA-DRA*0101_HLA-DRB1-4 |  |  |
| GGY*A*PAKAAAT | 6v19 | PAPPPISGGGYRARPAKAAATQKKVERKAP | 69-81 | 13 | P02675 | MH2 HLA-DRA*0101_HLA-DRB1-4 |  |  |
| GGYRA*PAKAAAT | 6v1a | PAPPPISGGGYRARPAKAAATQKKVERKAP | 69-81 | 13 | P02675 | MH2 HLA-DRA*0101_HLA-DRB1-4 |  |  |
| GQVELGGGNAVEVCKGS | 6xc9 |  |  | 17 | no info | MH2 HLA-DQA1*0301_HLA-DQB1 | TRUE |  |
| GQVELGGGNAVEVCK | 6xco |  |  | 15 | no info | MH2 HLA-DQA1*0301_HLA-DQB1 | TRUE |  |
| GQVELGGGNAVEVCKGS | 6xcp |  |  | 17 | no info | MH2 HLA-DQA1*0301_HLA-DQB1 | TRUE | TRUE |
| AAPQPELPYPQPGSGGSIEGRGGSGA | 6xp6 |  |  | 26 | no info | MH2 HLA-DQA1*0501_HLA-DQB1 | TRUE | TRUE |
| AMERNAGSGIIISDGGGGSLVPRGS | 7kei |  |  | 25 | no info | MH2 HLA-DQA1*0102_HLA-DQB1*0602 | TRUE | TRUE |
| GGIGSDNKVTRRGG | 7n19 |  |  | 14 | no info | MH2 HLA-DRA1_HLA-DRB1 | TRUE | TRUE |

**Table S4**. Designed neoantigen candidates of hepatocellular carcinoma using EpiMII followed by in silico evaluation and final candidates’ selection.

**Table S5**. The total 66 HLA class II alleles frequencies cover around 99% of the population worldwide*. *The top 21 HLA-DRB1 alleles cover more than 95.54% (from DRB1*1501 to DRB1*1601), and the top 31 cover more than 99.14% of the population. The top 4 HLA-DQA1 alleles cover more than 94.9% (from DQA1*0501 to DQA1*0101), and the top 6 alleles cover more than 99.55% of the population. The top 7 HLA-DQB1 alleles cover more than 94.8% (from DQB1*0301 to DQB1*0402), and the top 16 alleles cover more than 98.5%. The top 2 HLA-DPA1 alleles cover more than 95.37% and the top three alleles cover more than 99.7% of the population. The top 6 HLA-DPB1 alleles cover more than 96.0% (from DPB1*0401 to DPB1*0301) and the top 9 alleles cover more than 99.0% of the population. Year is the year the allele was first described in the literature. CWD demonstrated that the allele is considered common and well-documented. Freq is the overall average frequency across all population samples. Region is the number of world regions in which the allele appears.

| Locus | Allele | Year | CWD | Freq | Regions |
| --- | --- | --- | --- | --- | --- |
| DRB1 | DRB1*1501 | 1987 | CWD | 0.07864 | 11 |
|  | DRB1*0701 | 1986 | CWD | 0.06986 | 11 |
|  | DRB1*0301 | 1984 | CWD | 0.0676 | 11 |
|  | DRB1*1101 | 1986 | CWD | 0.05945 | 10 |
|  | DRB1*0901 | 1986 | CWD | 0.0545 | 10 |
|  | DRB1*0405 | 1986 | CWD | 0.04776 | 11 |
|  | DRB1*1502 | 1986 | CWD | 0.04507 | 11 |
|  | DRB1*0101 | 1985 | CWD | 0.04123 | 11 |
|  | DRB1*0803 | 1989 | CWD | 0.03864 | 10 |
|  | DRB1*1302 | 1988 | CWD | 0.03746 | 10 |
|  | DRB1*1401 | 1986 | CWD | 0.03218 | 11 |
|  | DRB1*1301 | 1986 | CWD | 0.03152 | 11 |
|  | DRB1*0401 | 1985 | CWD | 0.02896 | 11 |
|  | DRB1*1202 | 1989 | CWD | 0.02896 | 8 |
|  | DRB1*1602 | 1988 | CWD | 0.0285 | 11 |
|  | DRB1*1201 | 1989 | CWD | 0.02712 | 11 |
|  | DRB1*0403 | 1985 | CWD | 0.02659 | 11 |
|  | DRB1*0802 | 1989 | CWD | 0.02104 | 10 |
|  | DRB1*0404 | 1985 | CWD | 0.01795 | 11 |
|  | DRB1*1104 | 1986 | CWD | 0.0178 | 10 |
|  | DRB1*1601 | 1986 | CWD | 0.01656 | 9 |
|  | DRB1*1402 | 1989 | CWD | 0.01649 | 10 |
|  | DRB1*0407 | 1990 | CWD | 0.01536 | 10 |
|  | DRB1*1001 | 1984 | CWD | 0.01284 | 11 |
|  | DRB1*0406 | 1987 | CWD | 0.01194 | 9 |
|  | DRB1*0102 | 1988 | CWD | 0.01161 | 8 |
|  | DRB1*0411 | 1991 | CWD | 0.00966 | 6 |
|  | DRB1*1503 | 1991 | CWD | 0.00877 | 10 |
|  | DRB1*0801 | 1987 | CWD | 0.00875 | 10 |
|  | DRB1*1303 | 1990 | CWD | 0.00784 | 10 |
|  | DRB1*1405 | 1990 | CWD | 0.00759 | 4 |
|  | DRB1*0402 | 1986 | CWD | 0.00742 | 9 |
| DQA1 | DQA1*0501 | 1984 |  | 0.25958 | 11 |
|  | DQA1*0301 | 1982 |  | 0.21576 | 11 |
|  | DQA1*0102 | 1984 |  | 0.17286 | 11 |
|  | DQA1*0101 | 1987 |  | 0.12682 | 11 |
|  | DQA1*0201 | 1983 |  | 0.0884 | 11 |
|  | DQA1*0103 | 1987 |  | 0.06956 | 11 |
| DQB1 | DQB1*0301 | 1987 | CWD | 0.22376 | 11 |
|  | DQB1*0201 | 1984 | CWD | 0.15003 | 11 |
|  | DQB1*0302 | 1983 | CWD | 0.11539 | 11 |
|  | DQB1*0501 | 1985 | CWD | 0.09307 | 11 |
|  | DQB1*0602 | 1987 | CWD | 0.08062 | 11 |
|  | DQB1*0303 | 1987 | CWD | 0.05764 | 11 |
|  | DQB1*0402 | 1987 | CWD | 0.05259 | 11 |
|  | DQB1*0502 | 1987 | CWD | 0.05146 | 11 |
|  | DQB1*0601 | 1987 | CWD | 0.04898 | 11 |
|  | DQB1*0503 | 1987 | CWD | 0.03481 | 11 |
|  | DQB1*0603 | 1987 | CWD | 0.0315 | 11 |
|  | DQB1*0604 | 1987 | CWD | 0.02503 | 9 |
|  | DQB1*0401 | 1986 | CWD | 0.01928 | 10 |
|  | DQB1*0605 | 1989 | CWD | 0.00912 | 10 |
|  | DQB1*0304 | 1992 | CWD | 0.00238 | 7 |
|  | DQB1*0305 | 1993 | CWD | 0.00072 | 6 |
| DPA1 | DPA1*0103 | 1984 |  | 0.5831 | 9 |
|  | DPA1*0201 | 1985 |  | 0.20172 | 9 |
|  | DPA1*0202 | 1992 |  | 0.16164 | 9 |
| DPB1 | DPB1*0401 | 1984 | CWD | 0.23267 | 11 |
|  | DPB1*0402 | 1988 | CWD | 0.18989 | 10 |
|  | DPB1*0201 | 1983 | CWD | 0.12895 | 11 |
|  | DPB1*0501 | 1988 | CWD | 0.11547 | 11 |
|  | DPB1*0101 | 1988 | CWD | 0.06816 | 11 |
|  | DPB1*0301 | 1984 | CWD | 0.06572 | 11 |
|  | DPB1*1401 | 1989 | CWD | 0.04065 | 10 |
|  | DPB1*0902 | 1989 | CWD | 0.038 | 10 |
|  | DPB1*1701 | 1989 | CWD | 0.02228 | 9 |

**Table S6**. The analysis of the length of G-alpha and G-beta for different types of MHC-II using 11 entries collected from the IMGT database.

| IMGT number | MHC-II types | G-alpha range | G-beta range |
| --- | --- | --- | --- |
| 1bx2 | DRA*0101_DRB1*1501 | Jan-83 | Jan-92 |
| 1dlh | DRA*0101_DRB1*0101 | Jan-82 | Jan-92 |
| 2seb | DRA*0101_DRB1*0401 | Jan-84 | Jan-93 |
| 3c5j | DRA*0101_DRB3*0301 | Jan-82 | Jan-91 |
| 4mcy | DRA*0101_DRB*04 | Jan-82 | Jan-91 |
| 1jk8 | DQA1*0302_DQB1*0302 | Jan-84 | Jan-93 |
| 1s9v | DQA1*0501_DQB1*0201 | Jan-84 | Jan-93 |
| 5ksb | DQA1*0501_DQB1*0302 | Jan-84 | Jan-93 |
| 4-May | DQA1*0102_DQB1*0501 | Jan-84 | Jan-92 |
| 3wex | DPA1*0202_DPB1*0501 | Apr-84 | Jun-92 |
| 4p4k | DPA1*0103_DPB1 | Jan-83 | Jun-92 |

**Table S7**. The number of epitopes that bind to different types of HLA class II alleles encoded MHC-II*. *For example, DPB1*0401;15;181’ means there are 181 15-mer epitopes on the IEDB that are reported to bind to DPB1*0401 encoded MHC-II, no matter which HLA allele encoded for alpha chains. Because DQB1*0601 and DRB1*0301have no reported 15-mer epitope on the IEDB, we deleted these two when we measure the correlation.

| DxB alleles | Length | Counts of binding epitopes |
| --- | --- | --- |
| DPB1*0401 | 15 | 181 |
| DPB1*0402 | 15 | 179 |
| DPB1*0201 | 15 | 218 |
| DPB1*0501 | 15 | 117 |
| DPB1*0101 | 15 | 155 |
| DPB1*0301 | 15 | 110 |
| DPB1*1401 | 15 | 2137 |
| DPB1*1701 | 15 | 1236 |
| DQB1*0201 | 15 | 1462 |
| DQB1*0301 | 15 | 1418 |
| DQB1*0302 | 15 | 2393 |
| DQB1*0303 | 15 | 430 |
| DQB1*0401 | 15 | 5 |
| DQB1*0402 | 15 | 632 |
| DQB1*0501 | 15 | 1134 |
| DQB1*0502 | 15 | 135 |
| DQB1*0503 | 15 | 9 |
| DQB1*0601 | 15 | 0 |
| DQB1*0602 | 15 | 1195 |
| DQB1*0603 | 15 | 126 |
| DQB1*0604 | 15 | 14 |
| DRB1*0101 | 15 | 1246 |
| DRB1*0102 | 15 | 883 |
| DRB1*0301 | 15 | 0 |
| DRB1*0401 | 15 | 5784 |
| DRB1*0402 | 15 | 3699 |
| DRB1*0403 | 15 | 91 |
| DRB1*0404 | 15 | 2749 |
| DRB1*0405 | 15 | 2357 |
| DRB1*0406 | 15 | 5 |
| DRB1*0407 | 15 | 102 |
| DRB1*0411 | 15 | 4 |
| DRB1*0701 | 15 | 706 |
| DRB1*0801 | 15 | 703 |
| DRB1*0802 | 15 | 1532 |
| DRB1*0803 | 15 | 375 |
| DRB1*0901 | 15 | 691 |
| DRB1*1001 | 15 | 54 |
| DRB1*1101 | 15 | 596 |
| DRB1*1104 | 15 | 74 |
| DRB1*1201 | 15 | 1212 |
| DRB1*1202 | 15 | 93 |
| DRB1*1301 | 15 | 625 |
| DRB1*1302 | 15 | 2 |
| DRB1*1303 | 15 | 619 |
| DRB1*1401 | 15 | 253 |
| DRB1*1402 | 15 | 91 |
| DRB1*1405 | 15 | 333 |
| DRB1*1501 | 15 | 4414 |
| DRB1*1502 | 15 | 65 |
| DRB1*1503 | 15 | 19 |
| DRB1*1601 | 15 | 103 |
| DRB1*1602 | 15 | 180 |

**Table S8**. Detailed TCR information of T cell clones with clone count ≥5 after WT-peptide stimulation.

| Clone ID | TRA_V  gene | TRA_J  gene | TRA_CDR3_AA | TRA_C gene | TRB_V  gene | TRB_D  gene | TRB_J  gene | TRB_CDR3_AA | TRB_C  gene | Clone  size | Clone percentage (%) |
| --- | --- | --- | --- | --- | --- | --- | --- | --- | --- | --- | --- |
| Clone 1 | TRAV8-3 | TRAJ39 | CAVGDNAGNMLTF | TRAC | TRBV20-1 | TRBD1*01 F | TRBJ2-1 | CSASQRDRGRNEQFF | TRBC2 | 7 | 4.76 |
| Clone 2 | TRAV8-4 | TRAJ44 | CAVSITGTASKLTF | TRAC | TRBV6-1 | FTRBD2*02 F | TRBJ2-1 | CASRTLRRGRYNEQFF | TRBC2 | 7 | 4.76 |
| Clone 3 | TRAV29/DV5 | TRAJ28 | CAARGAGSYQLTF | TRAC | TRBV10-2 | TRBD1*01 F | TRBJ1-1 | CASRAGGSTEAFF | TRBC1 | 5 | 3.4 |

**Table S9**. Detailed TCR information of T cell clones with clone count ≥5 after MUT-peptide stimulation.

| Clone ID | TRA_V  gene | TRA_J  gene | TRA_CDR3_AA | TRA_C gene | TRB_V  gene | TRB_D  gene | TRB_J  gene | TRB_CDR3_AA | TRB_C  gene | Clone  size | Clone percentage (%) |
| --- | --- | --- | --- | --- | --- | --- | --- | --- | --- | --- | --- |
| Clone 1 | TRAV8-3 | TRAJ39 | CAVGDNAGNMLTF | TRAC | TRBV20-1 | TRBD1*01 F | TRBJ2-1 | CSASQRDRGRNEQFF | TRBC2 | 36 | 10.75 |
| Clone 2 | TRAV22 | TRAJ13 | CAVAGGYQKVTF | TRAC | TRBV30 | TRBD1*01 F | TRBJ1-6 | CAWSVEGKGSPLHF | TRBC1 | 25 | 7.46 |
| Clone 3 | TRAV17 | TRAJ40 | CATVPSGTYKYIF | TRAC | TRBV27 | TRBD1*01 F | TRBJ1-1 | CASAIRVGPSGVEAFF | TRBC1 | 11 | 3.28 |
| Clone 4 | TRAV12-1 | TRAJ12 | CVVNEKDSSYKLIF | TRAC | TRBV6-1 | TRBD1*01 F | TRBJ1-3 | CASSENGASSGNTIYF | TRBC1 | 10 | 2.99 |
| Clone 5 | TRAV29/DV5 | TRAJ28 | CAARGAGSYQLTF | TRAC | TRBV10-2 | TRBD1*01 F | TRBJ1-1 | CASRAGGSTEAFF | TRBC1 | 10 | 2.99 |
| Clone 6 | TRAV16 | TRAJ39 | CALNNAGNMLTF | TRAC | TRBV19 | TRBD2*01 F | TRBJ2-4 | CASSIGGDIQYF | TRBC2 | 9 | 2.69 |
| Clone 7 | TRAV12-2 | TRAJ45 | CAVTLYSGGGADGLTF | TRAC | TRBV19 | TRBD2*01 F | TRBJ2-5 | CASSPASEQETQYF | TRBC2 | 8 | 2.39 |
| Clone 8 | TRAV38-2/DV8 | TRAJ43 | CAYRSARNNNDMRF | TRAC | TRBV7-9 | TRBD1*01 F | TRBJ1-1 | CASSLRLNTEAFF | TRBC1 | 6 | 1.79 |
| Clone 9 | TRAV13-1 | TRAJ18 | CAAIRRGSTLGRLYF | TRAC | TRBV28 | TRBD1*01 F | TRBJ2-7 | CASSLWTGRNEQYF | TRBC2 | 5 | 1.49 |
| Clone 10 | TRAV8-4 | TRAJ23 | CAVSAPYNQGGKLIF | TRAC | TRBV20-1 | TRBD1*01 F | TRBJ2-3 | CSARDPGASTDTQYF | TRBC2 | 5 | 1.49 |

**Table S10**. TCR sequence information of shared T cell clones after WT-peptide and MUT-peptide stimulation.

| Clone | TRA_V gene | TRA_Amino Acids | TRA_Nucleotides | TRB_V gene | TRB_Amino Acids | TRB_Nucleotides |
| --- | --- | --- | --- | --- | --- | --- |
| Clone 1 | TRAV8-3 | AQSVTQPDIHITVSEGASLELRCNYSYGATPYLFWYVQSPGQGLQLLLKYFSGDTLVQGIKGFEAEFKRSQSSFNLRKPSVHWSDAAEYFCAVGDNAGNMLTFGGGTRLMVKP | GCCCAGTCAGTGACCCAGCCTGACATCCACATCACTGTCTCTGAAGGAGCCTCACTGGAGTTGAGATGTAACTATTCCTATGGGGCAACACCTTATCTCTTCTGGTATGTCCAGTCCCCCGGCCAAGGCCTCCAGCTGCTCCTGAAGTACTTTTCAGGAGACACTCTGGTTCAAGGCATTAAAGGCTTTGAGGCTGAATTTAAGAGGAGTCAATCTTCCTTCAACCTGAGGAAACCCTCTGTGCATTGGAGTGATGCTGCTGAGTACTTCTGTGCTGTGGGGGATAATGCAGGCAACATGCTCACCTTTGGAGGGGGAACAAGGTTAATGGTCAAACCCC | TRBV20-1 | GAVVSQHPSRVICKSGTSVKIECRSLDFQATTMFWYRQFPKQSLMLMATSNEGSKATYEQGVEKDKFLINHASLTLSTLTVTSAHPEDSSFYICSASQRDRGRNEQFFGPGTRLTVL | GGTGCTGTCGTCTCTCAACATCCGAGCAGGGTTATCTGTAAGAGTGGAACCTCTGTGAAGATCGAGTGCCGTTCCCTGGACTTTCAGGCCACAACTATGTTTTGGTATCGTCAGTTCCCGAAACAGAGTCTCATGCTGATGGCAACTTCCAATGAGGGCTCCAAGGCCACATACGAGCAAGGCGTCGAGAAGGACAAGTTTCTCATCAACCATGCAAGCCTGACCTTGTCCACTCTGACAGTGACCAGTGCCCATCCTGAAGACAGCAGCTTCTACATCTGCAGTGCTAGTCAACGGGACAGGGGAAGGAATGAGCAGTTCTTCGGGCCAGGGACACGGCTCACCGTGCTAG |
| Clone 2 | TRAV29/DV5 | DQQVKQNSPSLSVQEGRISILNCDYTNSMFDYFLWYKKYPAEGPTFLISISSIKDKNEDGRFTVFLNKSAKHLSLHIVPSQPGDSAVYFCAARGAGSYQLTFGKGTKLSVIP | GACCAGCAAGTTAAGCAAAATTCACCATCCCTGAGCGTCCAGGAAGGAAGAATTTCTATTCTGAACTGTGACTATACTAACAGCATGTTTGATTATTTCCTATGGTACAAAAAATACCCTGCTGAAGGTCCTACATTCCTGATATCTATAAGTTCCATTAAGGATAAAAATGAAGATGGAAGATTCACTGTCTTCTTAAACAAAAGTGCCAAGCACCTCTCTCTGCACATTGTGCCCTCCCAGCCTGGAGACTCTGCAGTGTACTTCTGTGCAGCAAGGGGGGCTGGGAGTTACCAACTCACTTTCGGGAAGGGGACCAAACTCTCGGTCATACCAA | TRBV10-2 | DAGITQSPRYKITETGRQVTLMCHQTWSHSYMFWYRQDLGHGLRLIYYSAAADITDKGEVPDGYVVSRSKTENFPLTLESATRSQTSVYFCASRAGGSTEAFFGQGTRLTVV | GATGCTGGAATCACCCAGAGCCCAAGATACAAGATCACAGAGACAGGAAGGCAGGTGACCTTGATGTGTCACCAGACTTGGAGCCACAGCTATATGTTCTGGTATCGACAAGACCTGGGACATGGGCTGAGGCTGATCTATTACTCAGCAGCTGCTGATATTACAGATAAAGGAGAAGTCCCCGATGGCTATGTTGTCTCCAGATCCAAGACAGAGAATTTCCCCCTCACTCTGGAGTCAGCTACCCGCTCCCAGACATCTGTGTATTTCTGCGCCAGCAGAGCAGGGGGTAGCACTGAAGCTTTCTTTGGACAAGGCACCAGACTCACAGTTGTAG |

**Table S11.** Detailed TCR Information for T Cell Clones Following P4 Peptide Stimulation.

| Clone ID | TRA_V  gene | TRA_J  gene | TRA_CDR3_AA | TRA_C gene | TRB_V  gene | TRB_D  gene | TRB_J  gene | TRB_CDR3_AA | TRB_C  gene | clone  _size |
| --- | --- | --- | --- | --- | --- | --- | --- | --- | --- | --- |
| Clone 1 | TRAV8-3 | TRAJ39 | CAVGDNAGNMLTF | TRAC | TRBV20-1 | TRBD1 | TRBJ2-1 | CSASQRDRGRNEQFF | TRBC2 | 6 |
